# Quaternary structure of *Artemia *haemoglobin II: analysis of T and C polymer alignment and interpolymer interface

**DOI:** 10.1186/1472-6807-7-26

**Published:** 2007-04-18

**Authors:** David T Chyou, Vincent L Rawle, Clive NA Trotman

**Affiliations:** 1 Department of Biochemistry, University of Otago, Dunedin, New Zealand

## Abstract

**Background:**

The brine shrimp *Artemia *expresses four different types of haemoglobin subunits namely C1, C2, T1 and T2. Two of these four subunits dimerize in different combinations to produce the three isoforms of the heterodimeric *Artemia *haemoglobin: HbI (C1 and C2), HbII (C1 and T2) and HbIII (T1 and T2). Previous biochemical, biophysical and computational analyses demonstrate that the T and C polymers are rings of nine concatenated globin domains, which are covalently joined by interdomain linkers. Two such rings stacked coaxially give the functional molecule. This research aimed to construct a quaternary structural model of *Artemia *HbII that shows the interpolymer interface and domain-domain alignment, using the MS3D (mass spectrometry for three dimensional analysis) approach. This involved introducing chemical crosslinks between the two polymers, cleaving with trypsin and analyzing the resulting products by mass spectrometry. This was followed by computational analysis of the mass spectrometry data using the program SearchXlinks to identify putatively crosslinked peptides.

**Results:**

Six putative EGS (ethylene glycol bis [succinimidylsuccinate]) crosslinked tryptic peptides were identified. All of them support a model in which the EF helices of all domains are in contact along the interpolymer surface, and Domain 1 of the T-polymer aligns with Domain 1 of the C-polymer. Any two adjacent interpolymer domain pairs contact through the early Helix H and early Helix A. The orientation of domains is different from the subunit proposed model proposed previously by this group. Crosslinking with GMBS (N- [γ-maleimidobutyryloxy]succinimide ester) was also performed, and the results show good agreement with this model.

**Conclusion:**

The interpolymer EF-contact allows the hydrophobic E and F helices to be buried in the interface and therefore allow the complex to solubilize readily to facilitate efficient oxygen transport. Furthermore the EF-contact is a common contact in cooperative haemoglobins and thus the model is consistent with the cooperative behaviour of *Artemia *HbII.

## Background

The brine shrimp *Artemia *can survive extreme environmental conditions, such as 1.5 M salinity and fluctuating oxygen concentrations. *Artemia *responds to environmental changes by switching between the two modes of reproduction: ovoviviparity and oviparity, in an oxygen concentration dependent manner. It has a high concentration of haemoglobin in the haemolymph, which also responds to the environment by changes in isoform content. The proposed quaternary structure of *Artemia *haemoglobin (two nine-domain polymer rings stacked coaxially) is unique among invertebrates (except for the related genus *Parartemia*). However, the structure has never been solved conclusively.

The brine shrimp *Artemia *expresses four different types of haemoglobin subunits namely C1, C2, T1 and T2. Two of these four subunits dimerize in different combinations to produce the three isoforms of the heterodimeric *Artemia *haemoglobin: HbI (C1 and C2), HbII (C1 and T2) and HbIII (T1 and T2). Each polymer consists of nine globin domains arranged in a ring and has a molecular weight of approximately 160 kDa. The mature molecule consists of two polymer rings stacked coaxially and has a molecular weight of approximately 320 kDa. Analysis of translated cDNA sequence, and partial trypsinolysis, confirmed that each polymer is made of nine concatenated globin domains, joined together via flexible linkers [1]. The orientation between each domain within each polymer was predicted from structural analysis of domain linkers [2], indicating a uniform, ring-like structure, characteristic of a repetitive linear concatenation of globin-like domains.

A linker of typically fourteen residues covalently joins each globin domain within each polymer. The inter-domain linkers contain the consensus sequence VDPVTGL [2]. Comparisons with pre-A leader sequences of globins from other organisms have led to the hypothetical extended and turn-associated linker structure [2]. The amino acid sequences of all *Artemia *inter-domain linkers were demonstrated to resemble closely the pre-A leader sequence of the *Petromyzon marinus *haemoglobin V [2] which has a known 3D structure. The predicted T and C polymer inter-domain linker structures, together with the highly conserved 3D structure of globin-family proteins, suggest a circular polymer structure.

The EM image obtained by Wood *et al*. (1981) [3] is consistent with the *ab initio *models of concatenated globin polymers, and with two coaxially stacked polymer rings. The measured sedimentation coefficient and frictional drag coefficient also correlate to this proposed quaternary structure [4]. The *Artemia *haemoglobins rarely dissociate into subunits in the absence of detergents even at low concentration. Dissociation would be disadvantageous to *Artemia*, as it would cause excretory loss of haemoglobin. Research by Moens *et al*. (1984) [5] revealed that when HbII was partially digested by subtilisin and analyzed by electrophoresis, bands with molecular weights in multiples of approximately 17,000 Da were formed. The partially digested sample can still bind oxygen non-cooperatively. The result further suggested that the subunits were polymers of myoglobin-like domains joined together by less folded loop-like structures that can be readily digested by proteases under partial proteolysis conditions. Intact *Artemia *haemoglobin cooperativity depends on the degree of oxygen saturation, temperature and pH [6,7].

The exact polymer interfaces and the domain-domain alignments in each *Artemia *haemoglobin isoform remain open questions to date. Previous attempts to crystalize the *Artemia *HbII have all been unsuccessful, possibly because of polymorphism in the primary sequence. It has been hypothesized previously that the E and F helices of both polymers were in contact based on the presence of surface-exposing hydrophobic residues [1].

The aim of this research was to find the quarternary structure of *Artemia *HbII, with both the polymer interfaces and domain-domain alignment identified. The technique of MS3D (mass spectrometry for three-dimensional analysis) [8-11] was utilized to achieve the aim. Crosslinks were introduced between the subunits of the native HbII molecule. The molecule was then cleaved with trypsin, and the resulting peptides isolated via reverse-phase high-performance liquid chromatography (RP-HPLC). The RP-HPLC fractions collected were then subjected to ESI-MS and significant peak were subjected to ESI-MS zoom scan to determine their charges. After charge determination a program called SearchXlinks (a program developed by Welfing *et al*., 2001) [12] was used to identify putatively crosslinked peaks. These peaks were then subjected to tandem mass spectrometry (MS/MS) to confirm the identity of the peptide. Haemoglobin II was chosen because it is made of one T-type (T2) and one C-type (C1) polymer, which share only 88% sequence identity and therefore have readily distinguishable sequences.

## Results and discussion

### Synthesis of EGS-crosslinked tryptic HbII fragments

Because the EGS/trypsin combination had not been used in previous research involving interface mapping by MS3D analysis, it was decided to characterize the digest by electrophoresis. Crosslinked proteolytic fragments can be distinguished on gels as banding differences between the crosslinked, trypsin treated sample and the non-crosslinked, trypsin treated sample.

**Figure 1:**
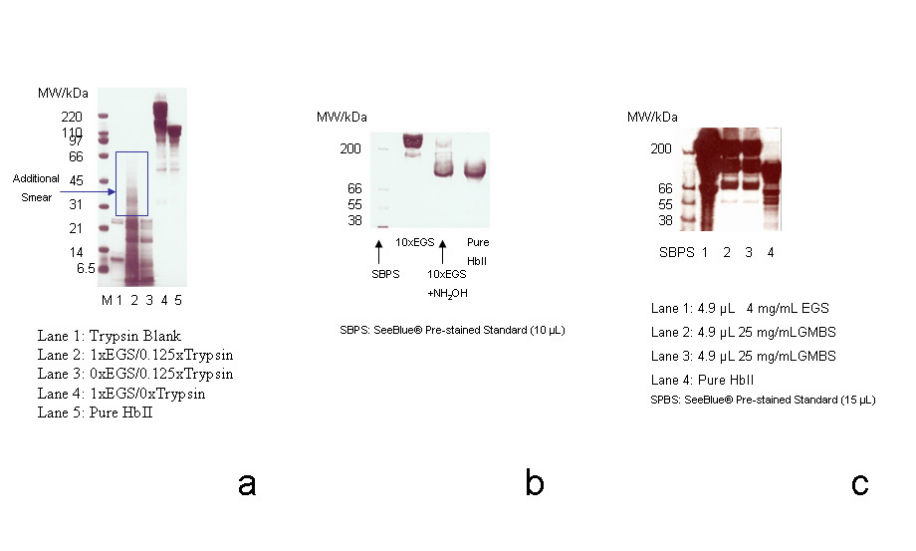
**(a) **Partial trypsinolysis of EGS-crosslinked HbII. HbII was crosslinked using 4.7 μL of 1 mg/mL EGS (prepared in DMSO) for one minutes at room temperature. Trypsinolysis was done using 20 μL 0.125x trypsin stock (2.5 mg/mL prepared in 25 mM HEPES pH7) for 3.5 hours at37°C. Each lane contains 20 μL of samples. **(b) **The effect of 1 M hydroxylamine-HCl on EGS-crosslinked HbII. The 2 M stock was prepared in sodium phosphate buffer, pH 8.5, and equal volume was added to the EGS-crosslinked HbII sample. Samples were incubated under 37°C for 6 hours with shaking. Each lane contains 35 μL of samples unless otherwise stated. **(c) **Crosslinking HbII using GMBS.

Based on the EGS+/Trypsin- sample (Figure 1a, Lane 4), compared with the EGS-/Trypsin- (unmodified and undigested HbII sample, Figure 1a, Lane 5), the 230 kDa band was interpreted as crosslinked HbII complex, and the 140 kDa band as individual HbII subunits. Although these molecular weight do not exactly coincide with the actual molecular weights of the HbII molecule and isolated subuits (320 kDa and 160 kDa respectively), crosslinking is known to alter migration characteristics of *Artemia *haemoglobins [3]. Pure HbII (Figure 1a, Lane 5) was observed in this experiment, as a band of approximately 140 kDa, comparable with the subunit molecular weight found by Moens and Kondo (1978) [13].

As shown in Figure 1a, a smear is present in the EGS+/Trypsin+ sample (Lane 2), above 25 kDa that is absent in the EGS-/Typsin+ sample (Lane 3). This smear is presumed to be the crosslinked material.

### EGS decrosslinking

One of the two replicates of EGS-crosslinked HbII was decrosslinked by treating it with hydroxylamine-HCl (Figure 1b). A major band with molecular weight of approximately 140 kDa was observed. The 140 kDa band is consistent with a single HbII subunit (Figure 1a, Lane 4). This result indicates that EGS-crosslinked HbII complexes were effectively decrosslinked by this method.

### GMBS crosslinking and the significance of the result

GMBS is a cysteine to lysine crosslinker with a spacer arm of 6.8 Å. Only four cysteine residues occur in HbII at position 2 of the A helices of domains C1, C2, T1 and T2. Figure 1c shows that inter-polymer crosslinking with GMBS was successful. This indicates that the A helices of the domains in HbII must be near the interface of the two polymer. This is consistent with the result obtained from EGS-crosslinking and mass spectrometry.

### Reverse-phase high-performance liquid chromatography fractionation

**Figure 2:**
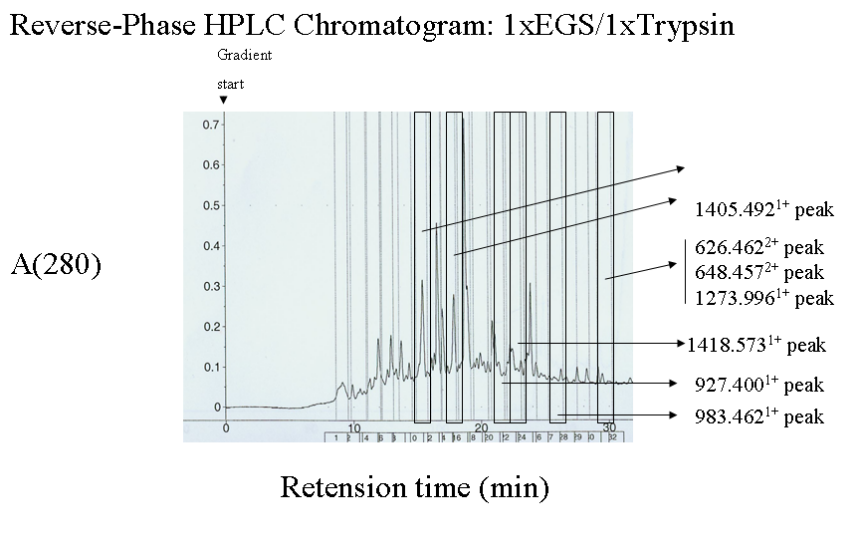
Reverse-phase high-performance liquid chromatography (RP-HPLC) chromatogram of completely trypsinolyzed HbII complex. ESI-MS peaks subjected to ESI-MS zoom scan and tandem MS analyses and their corresponding RP-HPLC fractions were indicated on this Figure. Charge-states of peaks (inferred based on the ESI-MS zoom scan data) were indicated as superscripts (see also Figure 3b inset).

**Figure 3:**
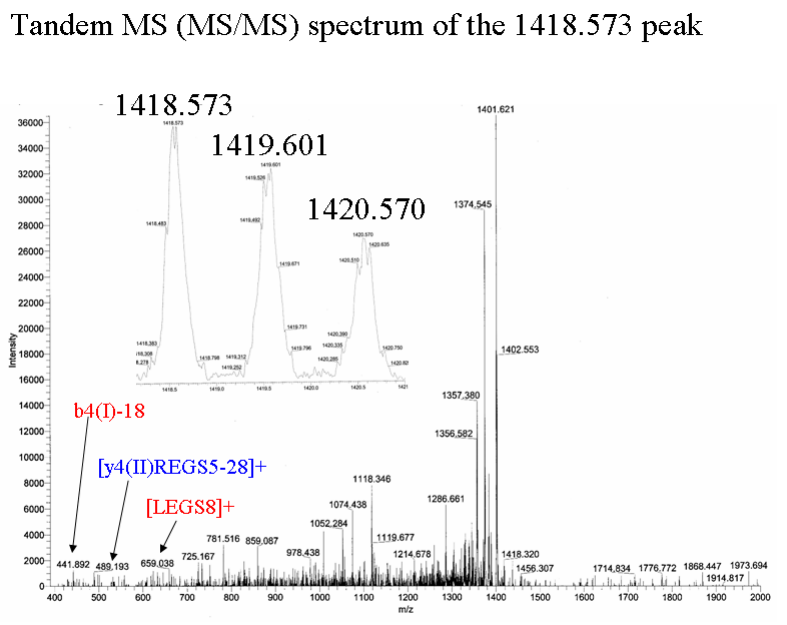
ESI-MS zoom scan results of the 1418.573 peak (inset) and corresponding tandem MS spectra; this spectrum is partially annotated. See Figure 4 for the enlarged version of this spectrum and further annotation.

Having characterized the EGS-crosslinked tryptic fragments by electrophoresis, the next step was to repeat the EGS+/Trypsin+ experiment but with complete trypsinolysis followed by RP-HPLC fractionation. The RP-HPLC chromatogram is presented in Figure 2. Putatively crosslinked peaks identified and subjected to tandem MS analysis and their corresponding RP-HPLC fractions are as indicated in Figure 2. Charge states of the peaks are labeled as the superscript above the m/z ratios of the peaks (Figure 2, see also Figure 3 inset).

### Identification of crosslinked lysine pairs: searchXlinks scoring and tandem mass spectra annotations

Setting a SearchXlinks score threshold of 20.0 [See Additional file 1], the three putative peptide species with the highest scores identified [See Additional file 2] were: GFK-GFK (927.400^1+ ^peak), HAISVTTK-EAIK (1542.509^1+ ^peak) and ASWNK-ATIKR (1418.573^1+ ^peak) (Table 1). When the SearchXlinks score threshold was reduced to the minimum (1.5) [See Additional file 3] and the peptide assignment receiving the best score was taken [See Additional file 4], five more putative peptide species were identified (Table 1). FindPept analysis again showed that none the peaks showed correlation to trypsin autolytic fragments. Tandem MS spectra of the 1418.573 peak and the zoom scan were shown as Figure 3. Red labels indicate peaks recognized by SearchXlinks during the scoring processes. In all cases, many peaks remained unannotated after SearchXlinks scoring.

**Figure 4:**
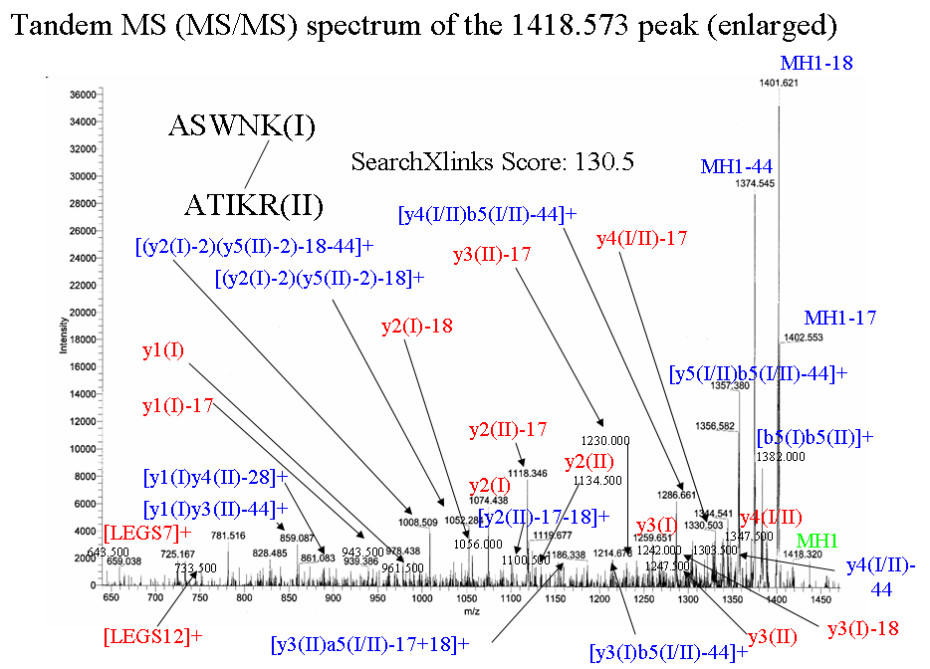
The enlarged version of the tandem MS spectra of the 1418.573 peak. The putative peptide species are those which received the best SearchXlinks score for a given m/z ratio, and which had a score above 20. The structure of the putative peptide species and the score received is indicated. The "|" sign joins crosslinked lysines. Fragment ions recognized by SearchXlinks during the scoring process were labelled red. Tandem MS precursor ion peak were labelled green, and the remaining identified peaks were labelled blue.

Reconfirmations (performed manually) were done to four of the putative crosslinked peptide assignments including assignment ASWNK-ATIKR, by checking whether any peaks unaccounted for by SearchXlinks PSD scoring function could be identified. Internal ions formed by receiving at least two b and y-type cleavages, and fragment ions formed from complicated fragmentation pathway were identified (blue labels) (Figure 3 and 4).

Ions formed from these complicated fragmentation pathways, (b and y-type fragment ions losing NH_3 _or H_2_O, or gaining H_2_O in the absence of the prerequisite amino acids) were identified. Fragment ions of types b/y-CO_2_, y-CO and y+H_2_O were also identified (Figure 4). Formations of all these ions are possible according to Schilling et al. (2003) [10]. The program MS2Assign [10] considers b or y-ions losing NH_3_, H_2_O, CO or CO_2_, or gaining of H_2_O in sequence-independent, and ion-type-independent manners.

The immonium y-ion is 2 Da lighter than the conventional, ammonium-type y-ion, hence it is referred to as the y-2 ion. Formation of such y-ions had been theorized by Biemann *et al*. (1992) [14]. Immonium-type y-ions were observed in the 927.400, 1542.509 and 1418.573 tandem mass spectra also.

It was noticed, on the 1418.573 tandem MS spectra (Figure 4) for instance, that peaks identified to be the non-polypeptide-characteristic internal ion peaks or peaks resulting from other complicated fragmentation pathways occupy a significant fraction, although the polypeptide-characteristic b/y-type ion peaks are present. It is therefore important to check whether the polypeptide-characteristic b/y-type ion matches are statistically significant.

The statistical significance of the b and y-type ion matching result can be evaluated according to Chen *et al*. (2001) [8]. Briefly, The probability that a random peak matched to a peak on the theoretical spectrum *by chance *(θ) can be expressed as:

θ=τr.
 MathType@MTEF@5@5@+=feaafiart1ev1aaatCvAUfKttLearuWrP9MDH5MBPbIqV92AaeXatLxBI9gBaebbnrfifHhDYfgasaacH8akY=wiFfYdH8Gipec8Eeeu0xXdbba9frFj0=OqFfea0dXdd9vqai=hGuQ8kuc9pgc9s8qqaq=dirpe0xb9q8qiLsFr0=vr0=vr0dc8meaabaqaciaacaGaaeqabaqabeGadaaakeaaiiGacqWF4oqCcqGH9aqpdaWcaaqaaiab=r8a0bqaaiabdkhaYbaacqGGUaGlaaa@3390@

Here *τ *is the sum of matching range of the theoretical spectrum, and r is the observing range of m/z charge ratio. Suppose that there are n random peaks, the probability that at least × of them matches, P(n, x, *θ*), can be expressed as:

P(n,x,θ)=1−∑k=0x−1(nk)θk(1−θ)n−k.
 MathType@MTEF@5@5@+=feaafiart1ev1aaatCvAUfKttLearuWrP9MDH5MBPbIqV92AaeXatLxBI9gBaebbnrfifHhDYfgasaacH8akY=wiFfYdH8Gipec8Eeeu0xXdbba9frFj0=OqFfea0dXdd9vqai=hGuQ8kuc9pgc9s8qqaq=dirpe0xb9q8qiLsFr0=vr0=vr0dc8meaabaqaciaacaGaaeqabaqabeGadaaakeaacqWGqbaucqGGOaakcqWGUbGBcqGGSaalcqWG4baEcqGGSaaliiGacqWF4oqCcqGGPaqkcqGH9aqpcqaIXaqmcqGHsisldaaeWbqaamaabmaabaqbaeqabiqaaaqaaiabd6gaUbqaaiabdUgaRbaaaiaawIcacaGLPaaaaSqaaiabdUgaRjabg2da9iabicdaWaqaaiabdIha4jabgkHiTiabigdaXaqdcqGHris5aOGae8hUde3aaWbaaSqabeaacqWGRbWAaaGccqGGOaakcqaIXaqmcqGHsislcqWF4oqCcqGGPaqkdaahaaWcbeqaaiabd6gaUjabgkHiTiabdUgaRbaakiabc6caUaaa@5367@

Hence the significance score, S, can be defined as:

*S *= -ln[*P*(*n*, *x*, *θ*)].

Statistical significances of the 927.400 and 1418.573 b/y-type ion matching results were measured (Table 2) using equations (1), (2) and (3). The theoretical tandem MS spectra of EGS-crosslinks GFK-GFK and ASWNK-ATIKR were also calculated. The theoretical tandem MS spectra contain only the polypeptide-characteristic b and y-ion, and the following b and y-ion derivatives b-H_2_O, b_n-1_+H_2_O, b-NH_3_, y-H_2_O and y-NH_3_, subject to the presence of prerequisite amino acids as defined by the developer of SearchXlinks. Both matching results are significant.

All crosslinked tryptic peptides identified suggest that trypsin can cleave after NHS-ester (*N*-hydroxylsuccinimide ester) crosslinker crosslinked lysine. This seems bizarre, as NHS-ester crosslinked lysine carries no (positive) charge. However, it had been reported that tryptic fragments of such type may be possible [8,9]. The crosslinker used by them, DSG (disuccinimidyl glutarate), is also an NHS-ester crosslinker as EGS. Therefore, it was decided not to reject any EGS-crosslinked tryptic peptides identified in which they were formed by receiving tryptic cleavage after EGS-crosslinked lysine because EGS is an NHS-ester crosslinker.

### Model construction

**Figure 5:**
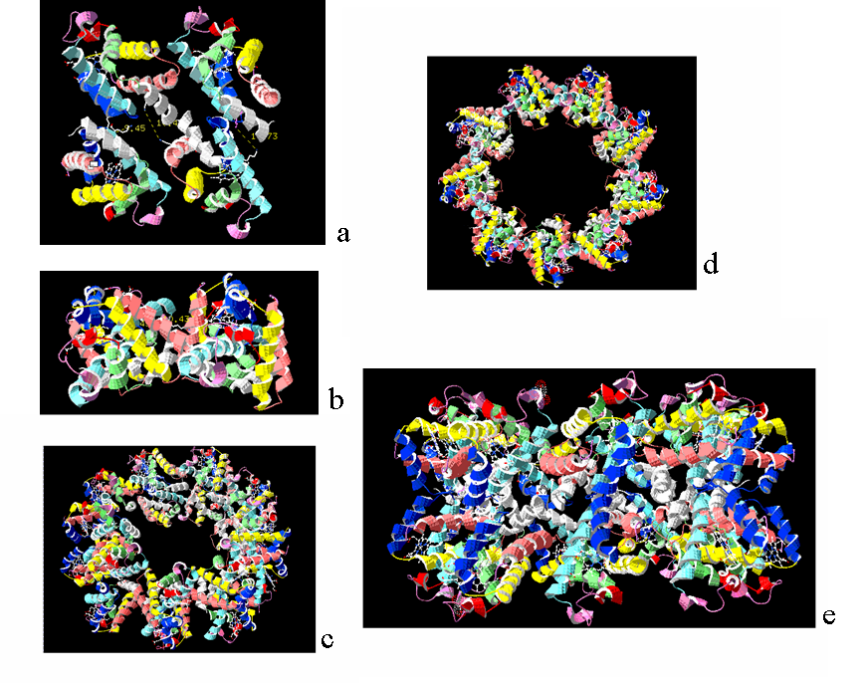
The front **(a) **and top **(b) **views of a section of the proposed EF: EF Domain 1: Domain 1 model of *Artemia *HbII complex. The EF: EF Domain 1: Domain 1 model of *Artemia *HbII complex is shown in full in **(c) **(front view), **(d) **(top view) and **(e) **(side view). The model was constructed using the software SPDBV37. Colour Codes: Helix A, grey; AB loop and helix B; green; Helix C, red; CD loop and helix D, purple; DE loop and helix E, cyan; EF loop and Helix F, blue; FG loop and helix G, yellow; GH loop and helix H; salmon.

**Figure 6:**
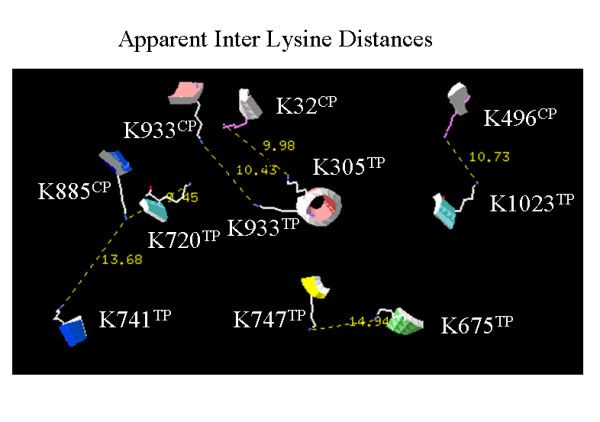
Inter-lysine (or lysine-cysteine) distances (in Å) residues which were experimentally shown to be crosslinked. The TP and CP superscripts indicate T or C polymer residues. Note that this figure is the same as Figure 5a except that only crosslinked residues are shown.

A model of the HbII quaternary structure was made using all crosslinked lysine pairs identified, by forcing the EF corners of two *Chironomus *globin III domains in contact to make a domain pair (Figure 5a). Then nine such domain pairs were rotated 40° to form a ring with the GH loop pointing into the centre of the ring (Figure 5a and 5b). The H helices of two adjacent domains in one polymer will mark an angle of approximately 40° (Figure 5b). Two adjacent domain pairs contact through Helix H (Figure 6). Therefore, if Domain 1 of one polymer is interacting with Domain 1 of the other polymer, late Helix E of Domain T5 can be recruited to close proximity to early Helix F of Domain C6 and *vice versa*. Mid Helix H of both T6 and C6 can also be recruited to close proximity.

The apparent inter-lysine distance between K933^TP ^and K933^CP^, K720^TP ^and K885^CP^, K305^TP ^and K32^CP ^and K1023^TP ^and K496^CP ^were measured as 10.43 Å, 9.45 Å, 9.60Å and 10.73 Å respectively (Figure 6), and all are comparable with the in-solution crossbridge span of EGS (9.8 ± 2.5 Å) [15]. Here K933^CP ^was considered as position 14 of Helix H while it is actually at position 13, and K1023^TP ^was considered as position 16 of Helix E while it is actually at position 14. The latter variation is crucial since positions 14 and 15 of Helix E are both hydrophobic phenylalanines on the model *Chironomus *Hb structure. In both case, the minor variations are tolerable since *Artemia *HbII domains are unlikely to have exactly the same structure as *Chironomus *domains although they share the globin fold. Position 14 of Helix H is a polar, surface-exposed threonine on the *Chironomus *Hb structure used for modelling, and therefore such change does not contradict with the hydrophilic property of lysine. Other lysines K933^TP^, K720^TP^, K885^CP^, K305^>TP^, K32^CP ^and K496^CP ^were all considered as residues at exact positions on the model.

Inter-lysine distances between K885^CP ^and K741^TP^, and between K747^TP ^and K675^TP ^(or between K747^CP ^and K675^CP^) are both within the maximum EGS crossbridge span of 16 Å, although they both are beyond the range of the in-solution crossbridge span of EGS (9.8 ± 2.5 Å) (Figure 6). At this stage, all crosslinked lysine pairs identified so far (6 in total, Table 1) were accounted for.

There are crosslinks in which we cannot judge whether it is inter-polymer or intra-polymer by sequence alone. In those cases we judged whether such crosslinked peptides are inter- or intra-polymer crosslinks by finding out in which instance the two crosslinked lysines can be placed within 16 Å (the maximum crossbridge length of EGS), given that the domains are uniformly orientated in each polymer.

The domain orientations in this model differ from the orientations suggested by Trotman *et al*. (1994) [2]. According to this model the GH loops of domains point into the tangential direction with respect to a ring-like polymer. This would not be a surprise since the subunit model proposed by Trotman was based on the predicted structure of interdomain linkers using lamprey Hb Pre-A leader structure as a model and not a true linker. However, the similar primary structure of the *Artemia *interdomain linkers, plus the presence of a conformationally-restricted proline at a conserved position may well be an indication that all interdomain linkers have similar tertiary structures, and this in turn hints at a repetitive arrangement of domains. Therefore, the uniformly arranged domains assumption features in the previous model (Figure 5a and 5b).

As a further scrutiny, the apparent cysteine-lysine distance between C178^TP ^(2^nd ^residue of Domain T2, modeled as the 2^nd ^residue of Helix A on the *Chironomus *Hb structure) and K28^TP ^(5^th ^residue of Domain C1, modelled as 5^th ^residue of Helix A on the *Chironomus *Hb structure) is measured as 6.66 Å (Figure 6) which is comparable with the maximum GMBS crossbridge span (6.8 Å). Therefore the model shown as Figure 6a and 6b agrees with the GMBS crosslinking results, although it is possible that the crosslink was formed between a different cysteine-lysine pair, or more than one cysteine-lysine pairs are crosslinked by GMBS simutaneously.

Taking the 2-domain-pair model shown in Figures 5a and 5b, adding one more identical domain pair and forcing this domain pair to interact with its adjacent domain pair in the same fashion as the two domain-pairs shown as Figures 5a and 5b gives a domain-pair triplet. The three domain pairs lie on the circumference of a circle and define a 2/3*π *arc. The 3 domain pairs arrange uniformly and repetitively. Duplicating this domain-pair triplet three times and joining them into a circle gives a ring of 9 domain pairs (Figure 5c, 5d and 5e).

### MS3D analysis using cysteine-lysine crosslinker GMBS: scrutinizing the Domain 1: Domain 1, EF: EF model

**Figure 7:**
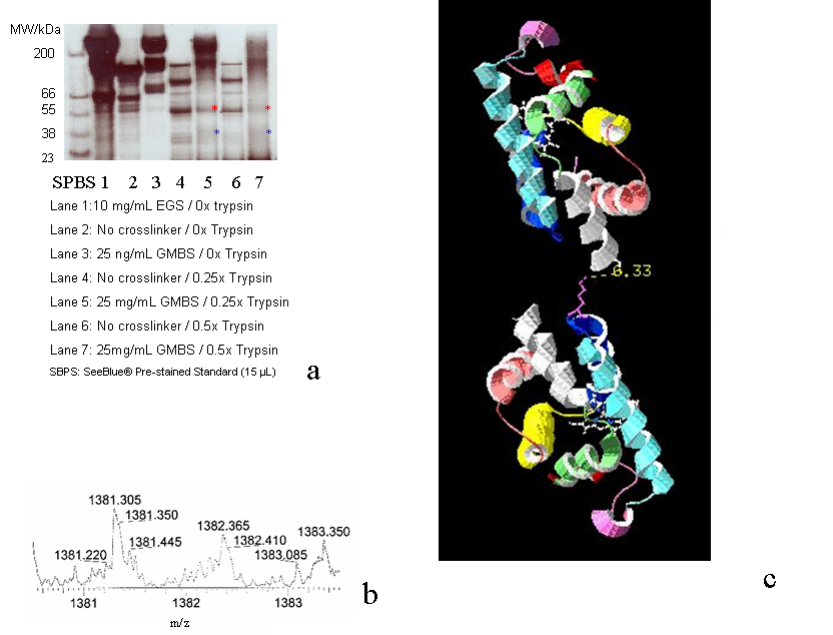
**(a) **Partial trypsinolysis of EGS-crosslinked HbII using 1.5-hour incubation time, 30 μL of samples were loaded on each lane unless otherwise mentioned. **(b) **The only genuine crosslinked peaks, the 1381.305^1+ ^peak, as zoom scan. **(c) **Orientation of Domain T1 and C1 which allows C25^CP ^and K106^TP ^to be positioned in a distance comparable to the expected 6.8Å (maximum GMBS crossbridge span).

HbII samples were crosslinked with GMBS and subjected to partial trypsinolysis under 37°C for 1.5 hours (Figure 7a). Additional smear in the trypsin+/GMBS+ lanes can be observed (Figure 7a, Lanes 5 and 7, blue star) by comparing both lanes with the corresponding GMBS- lanes (Figure 7a, Lanes 4 and 6 respectively), which can be GMBS-crosslinked HbII partial tryptic fragments. Bands marked with a red star (Figure 7a, Lanes 5 and 7) may be few Daltons heavier than the 55 kDa bands in Lanes 4 and 6 (Figure 7a). These bands may be GMBS-crosslinked HbII partial tryptic fragments, but it is also possible that the small migration difference may be simply due to experimental error.

HbII samples were crosslinked with same amount of GMBS and subjected to complete trypsinolysis under 37°C for 24 hours using 20 μL of 1× trypsin stock. The digestion mixture was fractionated by RP-HPLC. Fractions collected were subjected to ESI-MS and all (not just a selection) significant peaks were subjected to ESI-MS zoom scan to determine their charge states. After SearchXlinks analysis [See Additional file 5], only one peak, the 1381.305^1+ ^peak (Figure 7b), was a genuine crosslinked peak that was unequivocally assigned to GMBS-crosslink AVK-GILCSD(g-deco-K) (g-deco, GMBS-decorated) [See Additional file 6]. Findpept analysis showed that the 1381.305^1+ ^peak is not a trypsin autolytic product peak. There are three other putative crosslinked peaks, but all of them showed possible correlations to uncrosslinked HbII tryptic fragments with or without GMBS decoration. Therefore, only one pair of crosslinked residue can be conclusively identified: C25 of C-polymer and K106 of T-polymer.

**Figure 8:**
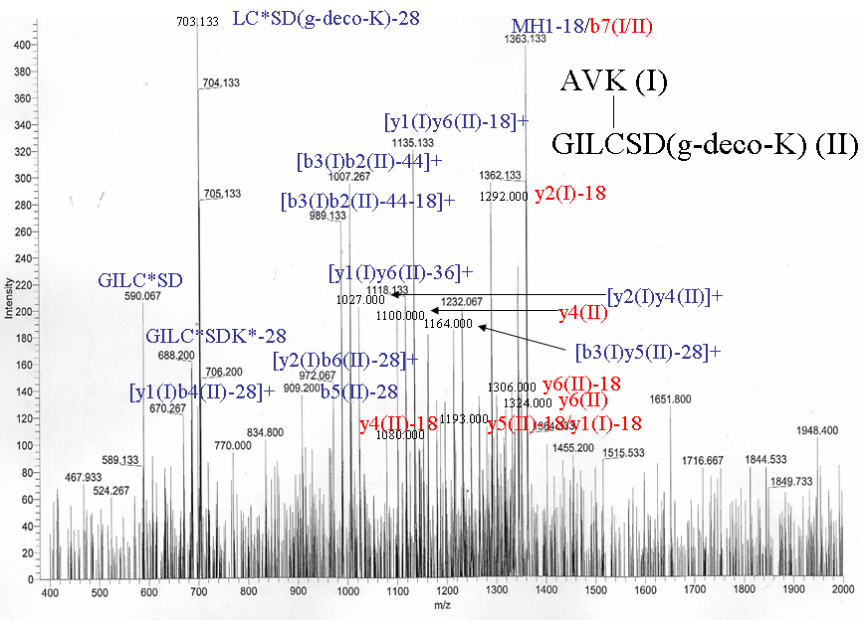
The tandem MS spectrum of the 1381.305^1+ ^peak. Fragment ions recognized by SearchXlinks during the scoring process were labelled red, and the remaining identified peaks were labelled blue.

The 1381.305 peak was then subjected to MS/MS (Figure 8), the GMBS-crosslink AVK-GILCSD(g-deco-K) received a score of 24.0 which is greater than the 20.0 threshold, and the following polypeptide-characteristic b/y-type ions were identified: b7(I/II), y1(I)-H_2_O, y2(I)-H_2_O, y4(II), y4(II)-H_2_O, y5(II)-H_2_O, y6(II) and y6(II)-H_2_O, here y5(II)-H_2_O and y1(I)-H_2_O ions have same molecular weight (1192 Da). Using Equation 2, the probability that these polypeptide-characteristic ions were matched by chance is only 0.0015 and hence the matching is significant (Table 2). A significant number of peaks remaining unaccounted after SearchXlinks scoring were identified, and most of them are internal ions with or without additional losses of H_2_O, CO or CO_2_. Ions carrying reverted modified residues were also identified (nomenclatures with star signs, Figure 8). Receiving a b/y-type cleavage at the sidechain-joining peptide bond can regenerate GMBS-modified lysine sidechain. GMBS-modified cysteine sidechains can be regenerated also according to Welfing *et al*. (2001), by a cleavage at the RS-R' bond of the sidechain-joining thioether group.

Putting all together, GMBS crosslinked residues were identified to be C25^CP ^and K106^TP^, rather than C178^TP ^and K32^CP ^as previously guessed. Nevertheless, both residues are on Domain 1 of T and C-polymer and hence agree with the Domain 1: Domain 1 alignment. The domains can be docked in a similar fashion as shown in Figure 4 while still having C25^CP ^and K106^TP ^positioned at a distance comparable with the maximum crossbridge span of GMBS (6.8 Å) (Figure 7c). Hence, the Domain 1: Domain 1, EF: EF model was further substantiated by the GMBS MS3D results.

## Conclusion

The aim of this research was to investigate the quaternary structure of *Artemia *HbII, and elucidate the details of domain-domain alignment and the interpolymer interface. The MS3D approach was employed. In total, six putative EGS-crosslinked tryptic peptides (Table 1), and 1 GMBS-crosslinked peptide (Figure 8) were identified through the MS3D experiments. Four of the seven crosslinked peptides mentioned above exceeded a threshold score of 20.0, while others appeared only when the minimum threshold score (1.5) was applied (Table 1). The identification of fragment ions carrying portions of EGS crossbridge (such as the [LEGS8]+ ions identified by SearchXlinks; Figure 3) provided further evidence of crossbridges.

Using the *Chironomus *globin III X-ray structure (1ECD) as a model, and applying the identified crosslinked lysine pairs showed that by having two globin domains in contact through EF corner, nine such domain pairs could be arranged into a ring with any two adjacent domain pairs contacting through the Helix H (Figure 5a and 5b). Domains T1 and C1 were then forced to align, which produced inter-lysine distances in four of the six crosslinked lysine pairs that were comparable with the in-solution crossbridge span of EGS (9.8 ± 2.5 Å). The other two were within the maximum crossbridge span of 16 Å.

The assumption can be made that the domains in each polymer are oriented in the same fashion and hence the two-domain-pair model shown as Figure 5a and 5b represents a section of the HbII complex. The model is consistent with the low resolution EM image of *Artemia *HbII [3] that suggests the molecule consists of two coaxially stacked ring-like globin polymers (Figure 5a, b, c, d and 5e).

The diameter: height ratio of the EF: EF, Domain 1: Domain 1 model is about 2.0 according to Figure 4e, which is comparable to the diameter: height ratio of 1.7 found by Wood *et al*. (1981) [3].

This EF: EF Domain 1: Domain 1 model further agrees with the GMBS crosslinking results since K106^TP ^and C25^CP ^can be positioned at a distance comparable with the maximum GMBS crossbridge span (6.8 Å) (Figure 7c) while having the domain orientations comparable to the model shown as Figure 5a and 5b.

This model suggests also that the two polymers are stacked in a symmetrical fashion, and therefore explains the dimeric nature of the complex. Having the two polymers stacked asymmetrically (for example, by having the EF helical regions contact with the GH helical regions), would likely mean that polymers would stack infinitely, since T and C polymers both have almost identical tertiary structure and primary structure. However there was no experimental evidence suggesting that T and C polymers can be stacked in this way.

Most importantly, having the EF helical region of each domain located at the interface explains the previous finding from sequence alignment studies that surface-exposed hydrophobic residues were observed most frequently at Helix E [1]. In order to solubilize the complex readily to facilitate efficient oxygen transport in an aqueous environment, these hydrophobic residues need to be buried.

Despite the diverse quaternary and primary structures of invertebrate haemoglobins, only one form of oligomeric assemblage, the EF-dimer (or EF-contacted single-domain subunits), has been found in more than one phylum. Such EF-contacts are commonly observed in many cooperative invertebrate types of haemoglobin also [16]. Therefore, the observed EF-contact between the T and C polymers may well explain the cooperative behavior of *Artemia *HbII. Although many cooperative types of haemoglobin contain EF-contact between subunits, the cooperativity mechanisms are various [16]. Therefore, exactly how the EF-contacting between the T and C polymer contributes to the cooperativity of *Artemia *HbII remains an open question.

## Methods

### EGS crosslinking

Crosslinking reactions were performed with 375 μg HbII, suspended in 25 mM HEPES, pH7 to a final volume of 48 μL. EGS (ethylene glycol *bis *[succinimidyl-succinate]) (4.7 μL of 1mg/mL EGS in DMSO) was added. The reaction was allowed to continue for 1 minute before quenching with 2.7 μL of 1 M Tris, (pH 7.5). The samples were incubated at room temperature for a further 15 minutes, before centrifuging for 10 minutes to remove non-solubilized material. Supernatants were transferred to a new microcentrifuge tube.

### GMBS crosslinking

Crosslinking reactions were performed with 1 mg of HbII, suspended in 25 mM HEPES, pH 7.5 to a final volume of 195 μL. GMBS, (4.9 μL of 25 mg/mL in DMSO) was added to HbII samples. The reaction was allowed to proceed for 1 hour at room temperature before quenching with 50 μL of 1 M Tris, (pH 7.5) and 50 μL of 1M cysteine. The tubes were incubated at room temperature for a further 15 minutes after quenching. Crosslinked HbII samples were precipitated with 40% (w/v) ammonium sulphate and resuspended in 55.4 μL of 25 mM HEPES, pH 7.5.

### Trypsinolysis

Trypsin was made to a concentration of 20 mg/mL by addition to 25 mM HEPES pH 7. Trypsin stocks of various concentrations (20 μL) were added directly to the crosslinked haemoglobin II sample and then incubated for 3.5 or 24 hours at 37°C. The reaction was quenched by adding 5 μL of 10 mg/mL PMSF. For partial trypsinolysis, 2.5 mg/mL trypsin was used and the incubation time was 3.5 hours. For complete trypsinolysis 20 mg/mL trypsin was used and the incubation time was 24 hours.

### EGS decrosslinking

EGS decrosslinking was done by adding an equal volume of 2 M hydroxylamine-HCl (NH_2_OH.HCl), prepared in 200 mM sodium phosphate buffer pH 8.5 to EGS-crosslinked HbII samples followed by incubation at 37°C for 6 hours with shaking. The efficiency of decrosslinking was tested using undigested crosslinked HbII samples and analyzed by SDS-PAGE using the NuPAGE system.

### Gel electrophoresis

The Invitrogen NuPAGE system was used with the NuPAGE Novex 4-12% Bis-Tris Gel (10 well), and the SeeBlue Plus2 prestained standard. Sample preparation and electrophoresis conditions were as recommended by the manufacturer.

### Reverse-phase high-performance liquid chromatography

The organic solvent (solution B) used for RP-HPLC was 80% acetonitrile, 0.085% trifluoroacetic acid. The aqueous solution (solution A) used for RP-HPLC was 2% acetonitrile and 0.1% trifluoroacetic acid. Peptides were separated by a gradient of 100% solution A to 50% solution B over a period of 40 minutes at 1 mL per minute. Complete trypsinolysis was carried out as stated above. The digest was then made up to 600 μL in solution A. Fractions were collected and reduced to ^2^30 μL in a Speedvac and subject to mass spectrometry (see below).

### Mass spectrometry

Samples were submitted to the Protein Microchemistry Facility (PMF), Department of Biochemistry, University of Otago. ESI-MS (electrospray ionization mass spectrometry) was undertaken using a Thermoquest, Finnigan, LCQ Deca Electrospray Ion Trap Mass Spectrometer operating in positive ion mode. A selection of significant ESI-MS peaks observed was subjected to ESI-MS zoom scan analysis (for charge state determination). Then a selection of putatively crosslinked peaks were subjected to tandem MS (MS/MS) analysis (see below for more details). Tandem MS spectra were generated using 35 units of collision energy. Results were returned in the form of printouts of undeconvoluted MS/MS spectra.

### Mass spectrometry data analysis: identification of crosslinked lysine pairs

After the ESI-MS zoom scan analysis, peaks were analyzed once with SearchXlinks Version 3.0.10 [12] with the PSD scoring function turned off, to identify all possible peptide assignments of each peak given their charge-states, to see if any peaks showed possible correlation to crosslinked tryptic peptides of HbII. Findpept analyses were also performed on the same set of peaks, checking to see if there was possible correlation to trypsin autolytic products. Peaks showing possible correlation to crosslinked tryptic peptides of HbII but no possible correlation to trypsin autolytic products would then be subjected to tandem MS analysis and a second round of SearchXlink analysis to score each of the peptide assignments. This was done by matching peaks of the experimental tandem MS spectrum to the theoretical tandem MS spectrum of each peptide assignment, using the PSD scoring function of SearchXlinks. The matching error used was 2.0 Da. A monoisotopic mass type (default) was used for the PSD scoring function of SearchXlinks. Currently, tandem MS peak lists for SearchXlinks have to be generated manually. All tandem MS peaks distinguishable from background noise were considered. It is important to ensure that peaks show no possible correlation to trypsin autolytic products because the trypsin used in this research is autolytic, and the PSD (post source decay) scoring function of SearchXlinks can only handle one protein or protein complex at a time.

Currently, the PSD scoring function of SearchXlinks calculates theoretical tandem MS spectra containing the low energy, polypeptide-characteristic b and y-type ions (ions formed by receiving cleavage at a CO-NH bond), and possible fragment ions predicted to be concomitantly formed under low collision energy by receiving a cleavage at the crossbridge. The score provides an indication of how close the observed tandem MS spectrum resembles the tandem MS signature of a possible peptide assignment compared with other possible peptide assignments of the same m/z ratio. The SearchXlink scoring function (mathematical) was designed in such a way that b and y-type ion matches were weighted higher than fragment ions formed by receiving a cleavage at the crossbridge. For consecutive b and y-type ion matches, weight increases exponentially after each successful consecutive b and y-type ion match. Therefore the score reflects mainly the tandem MS signatures of polypeptides. Fragment ions due to crossbridge fragmentation (which are predicted to be concomitantly formed with b and y-type ions) have only small contributions to the score, as the formation of such ions is currently conjectural. Those peptide or crosslinked peptide assignments receiving the best SearchXlinks scores were considered as the putative peptide species represented by each peak.

After scoring each peptide assignment using the PSD scoring function of SearchXlinks, the peptide assignments receiving the best scores compared with all others of the same m/z ratio were considered as recommended by the program developers. The putative peptide species identified after SearchXlink scoring were then re-confirmed by checking to see whether any peaks, especially the dominating peaks, remaining unannotated after scoring using the PSD scoring function of SearchXlinks could at least be identified. Those unannotated peaks were likely to represent the non-polypeptide-characteristic internal ions receiving at least 2 b/y-type cleavages at peptide groups, although ions formed from more complicated fragmentation pathways may also be possibilities.

### Molecular modelling

Since *Artemia *and *Chironomus *are both arthropods, the X-ray structure of *Chironomus *globin III (PDB: 1ECD) was used to model the globin domains of *Artemia *HbII. Domains within T and C polymers were assumed to be repetitively arranged into a ring. Each polymer has nine globin domains. The inter-lysine distances determined were used to define the interploymer-contacting surface, subject to the constraints that domains within T and C polymers are repetitively arranged into a ring and both polymers are identical. Modeling was done using the program Swiss PDB Viewer 3.7 (SPDBV 3.7), by stacking together four *Chironomus *globins to model two domain pairs. Orientations of the four-globin structures were adjusted to best fit all identified inter-lysine distances, under the constraints that domains within T and C polymers are repetitively arranged into a ring and both polymers are identical.

Unless otherwise stated, residues on the structure 1ECD in which they can be mapped to the *exact *position as the crosslinked lysines identified were ''mutated'' *in silico *to lysine (on the basis of residue number of each secondary structures, for examples, position 3 of Helix A, position 2 of FG loop, etc.). The inter-lysine distance between any 2 lysines identified to be crosslinked were taken as distances between the nitrogen atoms of the -NH_2 _group.

## Competing interests

The author(s) declare that they have no competing interests.

## Authors' contributions

VLR developed cross-linking techniques; DTC applied cross-linking techniques, did the computing and analysis and mostly wrote the paper; CNAT conceived of the study, and participated in its design and coordination. All authors read and approved the final manuscript.

## Supplementary Material

Additional file 1 The SearchXlinks parameter files used for scoring EGS/trypsinolysis MS data with PSD-filter on, cut-off score = 20.0 Click here for file

Additional file 2 Raw SeachXlinks scoring outputs corresponding to parameter file parameter_TPCP_20.txt (Additional file 2) Click here for file

Additional file 3 The SearchXlinks parameter files used for scoring EGS/trypsinolysis MS data with PSD-filter on, cut-off score = 1.5 Click here for file

Additional file 4 Raw SeachXlinks scoring outputs corresponding to parameter file parameter_TPCP.txt (Additional file 1) Click here for file

Additional file 5 The SearchXlinks parameter files used for scoring GMBS/trypsinolysis MS data with PSD-filter on, cut-off score = 20.0 Click here for file

Additional file 6 Raw SeachXlinks scoring outputs corresponding to parameter file parameter_TPCP_gmbs.txt (Additional file 3). Click here for file

## Figures and Tables

**Table 1:** Putative peptide or EGS-crosslinked peptides identified from complete trypsinolysis of EGS-crosslinked HbII.

**Mass/charge ratio (m/z)**	**Charge state**^a^	**Putative peptide species**					
		(Peptide assignment receiving best SearchXlinks score)					
		
		Peptide 1^b^	Polymer, Domain^c^	Peptide 2^b^	Polymer, Domain^c^	SXL Score	Note^d^
626.462^f^	+2	715-FSSIG**K**-720	TP, Domain 5	883-QL**K**-885	TP or CP, Domain 6	11.5	XLP
648.457	+2	738-EHI**K**R-742	TP or CP, Domain 5	883-QL**K**-885	TP or CP, Domain 6	6.0	XLP
927.400^e,f^	+1	931-GF**K**-933	TP, Domain 6	931-GF**K**-933	CP, Domain 6	21.0	XLP
983.462	+1	1138-IFTKVFTK-1145	TP, Domain 8	-	-	10.0	UmP
1273.996	+1	68-NIPASELASSER-79	TP, Domain 1	-	-	16.0	UmP
1405.492	+1	672-VFA**K**-675	TP or CP, Domain 5	742-RGLSR**K**-747	TP or CP, Domain 5	11.5	XLP
1418.573^e,f^	+1	301-ASWN**K**-305	TP or CP, Domain 2	29-ATI**K**R-33	TP or CP, Domain 1	130.5	XLP
1542.509^f^	+1	1016-HAISVTT**K**-1023	TP or CP, Domain 7	493-EAI**K**-496	CP, Domain 4	153.0	XLP

**Mass/charge ratio (m/z)**	**Charge state**^a^	**Mass/charge Ratio Expected (m/z)**	**Total Possible Peptide Assignment**				

626.462^f^	+2	^626.323^	^6^				
648.457	+2	^648.348^	^7^				
927.400^e,f^	+1	^927.446^	^1^				
983.462	+1	^983.593^	^5^				
1273.996	+1	^1273.639^	^17^				
1405.492	+1	^1405.780^	^20^				
1418.573^e,f^	+1	^1418.727^	^5^				
1542.509^f^	+1	^1541.806^	^38^				

^a ^Charge state of the peaks were inferred from the ESI-MS zoom scan data obtained. The inferred charged states were confirmed by consulting the PMF.

^b ^Crosslinked lysines were in **bold**.

^c ^TP, T polymer; CP, C polymer.

^d ^XLP, EGS-crosslinked peptides; UmP, unmodified peptide.

^e ^Statistical significances of b and y-type ion matchings were measured for these peaks (See also Table 2).

^f ^Further identifications of peaks remaining unannotated after SearchXlinks scoring were done for these peaks, and afterwards a majority of significant peaks were identified. [See Additional file 1, 2, 3, 4]

**Table 2:** Statistical significances of the 927.400, 1381.305 and 1418.573 b and y-type ion matching result.^a^

Peak	Parameter^b^					Result			IATC^f^
(m/z)	n	x	*τ*	r	*θ*	1- [P(n, x, *θ*)]	P(n, x, *θ*)	S	(AU)

927.400^1+^	67	4	16.0^c^	1750	0.00914286	0.99661194	0.00338806	5.68749793	250.0
1418.573^1+^	95	17	94.0^d^	1650	0.05696970	0.99997508	2.4920E-05	10.5998440	1000.0
1381.305^1+^	37	7	72.0^e^	1620	0.04444444	0.99849872	1.5013E-03	6.5014354	100.0

^a ^Statistical significances of b and y-type ion matchings were measured only for the 927.400^1+ ^and 1418.573^1+ ^peaks of EGS-crosslinking experiment, and the 1381.305^1+ ^peak of GMBS-crosslinking experiment.

^b ^Parameters were evaluated manually, see Equations (1), (2) and (3) for definitions of the symbols.

^c ^Sum of matching range based on the theoretical tandem MS spectrum of EGS-crosslink GFK-GFK. Ions result from a b/y-type cleavage at the crossbridge peptide bonds were not considered. Terminal b/y-type ions were accounted.

^d ^Sum of matching range based on the theoretical tandem MS spectrum of EGS-crosslink ASWNK-ATIKR. Ions result from a b/y-type cleavage at the crossbridge peptide bonds were not considered. Terminal b/y-type ions were accounted.

^e ^Sum of matching range based on the theoretical tandem MS spectrum of GMBS-crosslink AVK-GILCSD(g-deco-K). Ions result from a b/y-type cleavage at the crossbridge peptide bonds were not considered. Terminal b/y-type ions were accounted.

^f ^IATC, ion abundance threshold cut-off; AU, arbitrary unit. Peaks with abundances below the specified IATC were not accounted in this statistics. The IATC values are decided based on both the severity of background noise interference and the maximum observed ion abundance. [See Additional file 5, 6]
