# Supplementary material for: Quaternary structure of Artemia haemoglobin II: analysis of T and C polymer alignment and interpolymer interface
Source: BMC Struct Biol. 2007 Apr 18;7:26. doi: 10.1186/1472-6807-7-26 (PMC1865544; doi:10.1186/1472-6807-7-26)
Supplement: Additional file 4 — Raw SeachXlinks scoring outputs corresponding to parameter file parameter_TPCP.txt (Additional file 1) [file 1472-6807-7-26-S4.doc]

**Raw SearchXlinks scoring results (PSD filter on, cut-off score=1.5)**

The raw tandems MS scoring results of all 8 peaks at a cut-off SearchXlinks score of 20.0 were lodged here. Corresponding parameter file is "parameter_TPCP.txt".

sxl version 3.0.10 checked in on Wed, 20 Oct 2004 18:53:51 +0200

!! INFO this is an html document to be viewed with a browser. if this document

!! INFO is loaded from disk into the browser, its filename must have the

!! INFO extension .html or .htm. to remove html tags, save the document as a

!! INFO text file from within your browser.

!! INFO this html document provides tooltips, i.e., moving the mouse pointer

!! INFO over a cross-linker or a modification of a peptide isomer outline will

!! INFO make your browser display the type of the cross-link or modification.

!! INFO however, this does not work for all browsers.

contents

********

list of amino acid chains

digest

sorted list of assignments

peptide isomers

psd analysis

protein coverage

peak list

psd peak lists

list of amino acid chains

*************************

chain id protein

global local label fasta comment

-------------------------------------------------------------------------------

1 1 1 >TP

2 1 2 >CP

digest

******

legend

| cleavage site after this residue

k cleavage site after this residue prevented by a known link/modification

cleavage rule: trypsin (K,R)-(*\P)

cleavage sites:

number of potential cleavages = 309

number of prevented cleavages = 0

...............................................................................

chain 1 (chain 1 of protein 1)

10 20 30 40 50

AEISG ILCSD KATIK RTWAT VTDLP SFGRN VFLSV FAAKP EYKNL FVEFR

| | | | | |

60 70 80 90 100

NIPAS ELASS ERLLY HGGRV LSSID EAIAG IDTPD RAVKT LLALG ERHIS

| | | | |

110 120 130 140 150

RGTVR RHFEA FSYAF IDELK QRGVE SADLA AWRRG WDNIV NVLEA GLLRR

| | | | | || ||

160 170 180 190 200

QIDLE VTGLS CVDVA NIQES WSKVS GDLKT TGSVV FQRMI NGHPE YQQLF

| | |

210 220 230 240 250

RQFRD VDLDK LGESN SFVAH VFRVV AAFDG IIHEL DNNQF IVSTL KKLGE

| | | | ||

260 270 280 290 300

QHIAR GTDIS HFQNF RVTLL EYLKE NGMNG AQKAS WNKAF DAFEK YISMG

| | | | | |

310 320 330 340 350

LSSLK RVDPI TGLSG LEKNA ILSTW GKVRG NLQEV GKATF GKLFT AHPEY

| | | | | | |

360 370 380 390 400

QQMFR FSQGM PLASL VESPK FAAHT QRVVS ALDQT LLALN RPSDF VYMIK

| | | |

410 420 430 440 450

ELGLD HINRG TDRSH FENYQ VVFIE YLKET LGDSL DEFTV KSFNH VFEVI

| | | |

460 470 480 490 500

ISFLN EGLRQ ADIVD PVTHL TGRQK EMIKA SWSKA RTDLR SLGQE LFMRM

| | | | | | | |

510 520 530 540 550

FKAHP EYQTL FVNKG FADVP LVSLR EDERF ISHMA NVLGG FDTLL QNLDE

| | | |

560 570 580 590 600

SSYFI YSLRN LGDAH IQRKA GTQHF RSFEA ILIPY LQESQ GLDAA SVEAW

| || |

610 620 630 640 650

KKFFD VSIGV IAQGL KVATS EEADP VTGLY GKEIV ALRQA FAAVT PRNVE

|| | | | |

660 670 680 690 700

IGKRV FAKLF AAHPE YKNLF KKFEQ YSVEE LPSTD AFHYH ISLVM NRFSS

|| | | || |

710 720 730 740 750

IGKVI DDNVS FVYLL KKLGR EHIKR GLSRK QFDQF VELYI AEISS ELSDT

| || | || ||

760 770 780 790 800

GRNGL EKVLT FATGV IEQGL FQLGQ VDSNT LTALE KQSIQ DIWSN LRSTG

| | | |

810 820 830 840 850

LQDLA VKIFT RLFSA HPEYK LLFTG RFGNV DNINE NAPFK AHLHR VLSAF

| | | | | |

860 870 880 890 900

DIVIS TLDDS EHLIR QLKDL GLFHT RLGMT RSHFD NFATA FLSVA QDIAP

| | | |

910 920 930 940 950

NQLTV LGRES LNKGF KLMHG VIEEG LLQLE RINPI TGLSA REVAV VKQTW

| | | | | |

960 970 980 990 1000

NLVKP DLMGV GMRIF KSLFE AFPAY QAVFP KFSDV PLDKL EDTPA VGKHA

| | | | |

1010 1020 1030 1040 1050

ISVTT KLDEL IQTLD EPANL ALLAR QLGED HIVLR VNKPM FKSFG KVLVR

| | | | | |

1060 1070 1080 1090 1100

LLEND LGQRF SSFAS RSWHK AYDVI VEYIE EGLQQ SYKQD PVTGI TDAEK

| | | | |

1110 1120 1130 1140 1150

ALVQE SWDLL KPDLL GLGRK IFTKV FTKHP DYQIL FTRTG FGDTP LTKLD

|| | | | |

1160 1170 1180 1190 1200

DNPAF GTHII KVMRA FDHVI QILGK PKTLM AYLRS VGADH IARNV ERRHF

| | | | | ||

1210 1220 1230 1240 1250

QAFSN ALIPV MQHEL KAQLR PDAVA AWRKG LDRII GIIDQ GLIGL KEVNP

| || | |

1260 1270 1280 1290 1300

QNAFS AYDIQ AVQRT WALAK PDLMG KGAMV FKQLF TDHGY QPLFS NLAQY

| | |

1310 1320 1330 1340 1350

EITGL EGSPE LNTHA RNVMA QLDTL VGSLQ NSIEL GQSLA QLGKD HVPRK

| | ||

1360 1370 1380 1390 1400

VNRVH FKDFA EHFIP LMKAD LGDEF TPLAE SAWKK AFDVM IATIE QGQRA

| | | || |

1410

RRSVA TFLTN PVA

||

...............................................................................

chain 2 (chain 1 of protein 2)

10 20 30 40 50

AEVRG ILCSD KATIK RTWSI VNDLP SFGRN VFLSV FAAKP EYKNL FVEFR

| | | | | | |

60 70 80 90 100

NIPAS ELANS ERLLY HGGRV LASID EVISE IDSPD SAAKK LVALG ERHIT

| | || |

110 120 130 140 150

RGTVR RHFEA FSYAF IDELK QRGVA SADLA AWRKG WDSIV DILEA GLLKR

| | | | | || ||

160 170 180 190 200

QIDLE VTGLS CVDVA NVQES WATVS ANLKN TGSIL FQRLI NDHPE YQQLF

| |

210 220 230 240 250

RQFRD VELAK LGESN GFVAH VFRVV AAFDG IIKEL DNNPF IVSTL KRLGE

| | | | | ||

260 270 280 290 300

QHIAR GTDIS HFQNF RTTLL VYLNE NGMNQ AQEAS WNKAF DAIEK YISIG

| | | |

310 320 330 340 350

LKSLG RVDPI TGLSG LEKNA ILNTW GKVRG NLQEV GKATF GKLFA AHPEY

| | | | | | |

360 370 380 390 400

QQMFR FFQGV QLAEL VDSPK FAAHT QRVVS ALDQT LLALN RPSDF VYMIK

| | | |

410 420 430 440 450

ELGLD HINRG TDRSH FENYQ VVFVE YLKET LGDSV DEFTV KSFNH VFEVI

| | | |

460 470 480 490 500

INFLN EGLRQ ANVVD PVTHL TGRQK EAIKA SWSVA RTDLR FLGQE LFMRM

| | | | | | |

510 520 530 540 550

FNLNP EYQSL FVNKG FADVP LVSLR EDERF ISHMA NVLRG FDTLL QNLDD

| | | |

560 570 580 590 600

TSYFV YALRN LGDAH IQRKA GTEHF RSFEA ILIPY LQESQ GLDAA GVEAW

| || |

610 620 630 640 650

KIFFD VSIGV IAQGL KVASS EEADP VTGLY GKEVV ALRQA FAAIS PRNVE

| | | | |

660 670 680 690 700

IGKRV FAKLF TSHPE YKNLF KKFEQ YSVEE LPSTD AFDYH ISLVM NRFSA

|| | | || |

710 720 730 740 750

VGKVI DDNVS FVYLL KKLGR EHIKR GLSRK QFDQF VELYI AEISP ELSET

| || | || ||

760 770 780 790 800

GRSGL EKVLT FATGV IEQGL FQLGQ VDSKA LTALE KQSIQ DIWTS LRPTG

| | | |

810 820 830 840 850

LEELA VKMFT RLFAD HPEYK LLFTG RLGNV DNINE NAPFR AHLHR VLSAF

| | | | | |

860 870 880 890 900

DIVIT SLDNN ALLIR QLKDL GLFHT RLGMT RAHFD NFATA FFSVA EDIVP

| | | |

910 920 930 940 950

NLLTA LGRES LGKGF KLMVA VIEEG LLQLE RIDPI TGLSV REVEV VKQTW

| | | | | |

960 970 980 990 1000

NLVKP DLMGV GMRIF KSLFE KFPAY QAVFP KFSDV PLDKL EDIPA VGKHA

| | | | | |

1010 1020 1030 1040 1050

ISVTT KLDEL IQTLD EPANL ALLAR QLGED HIVLG VNKPM FKSFG EVLVR

| | | |

1060 1070 1080 1090 1100

LLEND LGQRF SNFAS KSWHR AYDVI VEYIE EGLQQ SYKQD PVTGI TDAEK

| | | | |

1110 1120 1130 1140 1150

VLVQR SWELL KPDLL GLGRK IFGVI FTKHP EYQIL FTRVG FGDTP LTQLD

| || | |

1160 1170 1180 1190 1200

NNPAF GEHII KVMRA FDYVI RNLGK PKTLL AYLKN VGADH IARNV ERRHF

| | | | | | ||

1210 1220 1230 1240 1250

QAFSE ALIPV MQREL KAQLK PEAVA AWRKG LDRII GVIDQ GLLGL KEVNP

| | || | |

1260 1270 1280 1290 1300

QIAFS AADIE AIQKT WALAK PDLMG KGASV FRQLF TDHGY QPLFS NLVEY

| | |

1310 1320 1330 1340 1350

EVTGL EGSPE LNTHA RNVMA QLDTL VGSLQ NSIEL GKSLN QLGKD HVPRK

| | | ||

1360 1370 1380 1390 1400

VNKVH FDDFA EHFVP LMKAN LGDEF TPLAE SAWKK AFNVM VATIE QGQRA

| | || |

1410

RRSIA TFLTN PVA

||

array of base peptides:

legend

bpid base peptide id

cid chain id

c number of connectors for optional links

km number of known modifications

!! INFO here, calculated masses refer to unprotonated base peptides and take

!! INFO into account known modifications.

bpid cid nterm cterm c km m(calc) aa-sequence

-------------------------------------------------------------------------------

1 1 1 11 2 0 1134.559 AEISGILCSD K

2 1 12 15 1 0 431.274 ATIK

3 1 16 16 0 0 174.112 R

4 1 17 29 0 0 1449.725 TWATVTDLPS FGR

5 1 30 43 2 0 1611.866 NVFLSVFAAK PEYK

6 1 44 50 0 0 923.486 NLFVEFR

7 1 51 62 0 0 1272.631 NIPASELASS ER

8 1 63 69 0 0 814.445 LLYHGGR

9 1 70 86 0 0 1770.900 VLSSIDEAIA GIDTPDR

10 1 87 89 1 0 316.211 AVK

11 1 90 97 0 0 871.513 TLLALGER

12 1 98 101 0 0 511.287 HISR

13 1 102 105 0 0 431.249 GTVR

14 1 106 106 0 0 174.112 R

15 1 107 120 1 0 1715.819 HFEAFSYAFI DELK

16 1 121 122 0 0 302.170 QR

17 1 123 133 0 0 1173.578 GVESADLAAW R

18 1 134 134 0 0 174.112 R

19 1 135 149 0 0 1667.899 GWDNIVNVLE AGLLR

20 1 150 150 0 0 174.112 R

21 1 151 173 1 0 2533.237 QIDLEVTGLS CVDVANIQES WSK

22 1 174 179 1 0 617.338 VSGDLK

23 1 180 188 0 0 993.524 TTGSVVFQR

24 1 189 201 0 0 1631.788 MINGHPEYQQ LFR

25 1 202 204 0 0 449.239 QFR

26 1 205 210 1 0 703.339 DVDLDK

27 1 211 223 0 0 1461.736 LGESNSFVAH VFR

28 1 224 246 1 0 2542.343 VVAAFDGIIH ELDNNQFIVS TLK

29 1 247 247 1 0 146.106 K

30 1 248 255 0 0 922.498 LGEQHIAR

31 1 256 266 0 0 1320.621 GTDISHFQNF R

32 1 267 274 1 0 977.580 VTLLEYLK

33 1 275 283 1 0 947.413 ENGMNGAQK

34 1 284 288 1 0 604.297 ASWNK

35 1 289 295 1 0 826.386 AFDAFEK

36 1 296 305 1 0 1097.579 YISMGLSSLK

37 1 306 306 0 0 174.112 R

38 1 307 318 1 0 1227.671 VDPITGLSGL EK

39 1 319 327 1 0 988.534 NAILSTWGK

40 1 328 329 0 0 273.180 VR

41 1 330 337 1 0 843.445 GNLQEVGK

42 1 338 342 1 0 522.280 ATFGK

43 1 343 355 0 0 1666.792 LFTAHPEYQQ MFR

44 1 356 370 1 0 1589.812 FSQGMPLASL VESPK

45 1 371 377 0 0 829.419 FAAHTQR

46 1 378 400 1 0 2592.398 VVSALDQTLL ALNRPSDFVY MIK

47 1 401 409 0 0 1065.557 ELGLDHINR

48 1 410 413 0 0 447.208 GTDR

49 1 414 428 1 0 1914.951 SHFENYQVVF IEYLK

50 1 429 441 1 0 1452.698 ETLGDSLDEF TVK

51 1 442 459 0 0 2120.105 SFNHVFEVII SFLNEGLR

52 1 460 473 0 0 1520.795 QADIVDPVTH LTGR

53 1 474 475 1 0 274.164 QK

54 1 476 479 1 0 519.273 EMIK

55 1 480 484 1 0 577.286 ASWSK

56 1 485 486 0 0 245.149 AR

57 1 487 490 0 0 503.270 TDLR

58 1 491 499 0 0 1079.543 SLGQELFMR

59 1 500 502 1 0 424.214 MFK

60 1 503 514 1 0 1445.730 AHPEYQTLFV NK

61 1 515 525 0 0 1172.655 GFADVPLVSL R

62 1 526 529 0 0 547.224 EDER

63 1 530 559 0 0 3449.696 FISHMANVLG GFDTLLQNLD ESSYFIYSLR

64 1 560 568 0 0 1022.526 NLGDAHIQR

65 1 569 569 1 0 146.106 K

66 1 570 576 0 0 815.404 AGTQHFR

67 1 577 601 1 0 2764.396 SFEAILIPYL QESQGLDAAS VEAWK

68 1 602 602 1 0 146.106 K

69 1 603 616 1 0 1492.829 FFDVSIGVIA QGLK

70 1 617 632 1 0 1635.799 VATSEEADPV TGLYGK

71 1 633 638 0 0 699.428 EIVALR

72 1 639 647 0 0 959.519 QAFAAVTPR

73 1 648 653 1 0 658.365 NVEIGK

74 1 654 654 0 0 174.112 R

75 1 655 658 1 0 463.279 VFAK

76 1 659 667 1 0 1074.550 LFAAHPEYK

77 1 668 671 1 0 520.301 NLFK

78 1 672 672 1 0 146.106 K

79 1 673 697 0 0 3011.412 FEQYSVEELP STDAFHYHIS LVMNR

80 1 698 703 1 0 637.343 FSSIGK

81 1 704 716 1 0 1523.823 VIDDNVSFVY LLK

82 1 717 717 1 0 146.106 K

83 1 718 720 0 0 344.217 LGR

84 1 721 724 1 0 525.291 EHIK

85 1 725 725 0 0 174.112 R

86 1 726 729 0 0 431.249 GLSR

87 1 730 730 1 0 146.106 K

88 1 731 752 0 0 2546.217 QFDQFVELYI AEISSELSDT GR

89 1 753 757 1 0 559.297 NGLEK

90 1 758 786 1 0 3091.644 VLTFATGVIE QGLFQLGQVD SNTLTALEK

91 1 787 797 0 0 1358.694 QSIQDIWSNL R

92 1 798 807 1 0 1030.566 STGLQDLAVK

93 1 808 811 0 0 535.312 IFTR

94 1 812 820 1 0 1090.545 LFSAHPEYK

95 1 821 826 0 0 705.417 LLFTGR

96 1 827 840 1 0 1577.747 FGNVDNINEN APFK

97 1 841 845 0 0 632.351 AHLHR

98 1 846 865 0 0 2242.184 VLSAFDIVIS TLDDSEHLIR

99 1 866 868 1 0 387.248 QLK

100 1 869 876 0 0 957.503 DLGLFHTR

101 1 877 881 0 0 576.305 LGMTR

102 1 882 908 0 0 2931.488 SHFDNFATAF LSVAQDIAPN QLTVLGR

103 1 909 913 1 0 589.307 ESLNK

104 1 914 916 1 0 350.195 GFK

105 1 917 931 0 0 1735.929 LMHGVIEEGL LQLER

106 1 932 941 0 0 1040.598 INPITGLSAR

107 1 942 947 1 0 643.390 EVAVVK

108 1 948 963 1 0 1843.943 QTWNLVKPDL MGVGMR

109 1 964 966 1 0 406.258 IFK

110 1 967 981 1 0 1713.876 SLFEAFPAYQ AVFPK

111 1 982 989 1 0 919.465 FSDVPLDK

112 1 990 998 1 0 928.486 LEDTPAVGK

113 1 999 1006 1 0 855.481 HAISVTTK

114 1 1007 1025 0 0 2107.152 LDELIQTLDE PANLALLAR

115 1 1026 1035 0 0 1178.641 QLGEDHIVLR

116 1 1036 1042 2 0 862.473 VNKPMFK

117 1 1043 1046 1 0 437.227 SFGK

118 1 1047 1050 0 0 485.333 VLVR

119 1 1051 1059 0 0 1056.556 LLENDLGQR

120 1 1060 1066 0 0 800.382 FSSFASR

121 1 1067 1070 1 0 556.276 SWHK

122 1 1071 1088 1 0 2146.047 AYDVIVEYIE EGLQQSYK

123 1 1089 1100 1 0 1272.620 QDPVTGITDA EK

124 1 1101 1119 1 0 2122.178 ALVQESWDLL KPDLLGLGR

125 1 1120 1120 1 0 146.106 K

126 1 1121 1124 1 0 507.306 IFTK

127 1 1125 1128 1 0 493.290 VFTK

128 1 1129 1138 0 0 1288.656 HPDYQILFTR

129 1 1139 1148 1 0 1035.524 TGFGDTPLTK

130 1 1149 1161 1 0 1439.741 LDDNPAFGTH IIK

131 1 1162 1164 0 0 404.221 VMR

132 1 1165 1177 2 0 1464.845 AFDHVIQILG KPK

133 1 1178 1184 0 0 866.468 TLMAYLR

134 1 1185 1193 0 0 924.478 SVGADHIAR

135 1 1194 1197 0 0 516.266 NVER

136 1 1198 1198 0 0 174.112 R

137 1 1199 1216 1 0 2109.083 HFQAFSNALI PVMQHELK

138 1 1217 1228 0 0 1352.731 AQLRPDAVAA WR

139 1 1229 1229 1 0 146.106 K

140 1 1230 1233 0 0 459.244 GLDR

141 1 1234 1246 1 0 1351.844 IIGIIDQGLI GLK

142 1 1247 1264 0 0 2048.991 EVNPQNAFSA YDIQAVQR

143 1 1265 1276 2 0 1329.711 TWALAKPDLM GK

144 1 1277 1282 1 0 651.341 GAMVFK

145 1 1283 1316 0 0 3845.864 QLFTDHGYQP LFSNLAQYEI TGLEGSPELN

THAR

146 1 1317 1344 1 0 2926.543 NVMAQLDTLV GSLQNSIELG QSLAQLGK

147 1 1345 1349 0 0 622.319 DHVPR

148 1 1350 1350 1 0 146.106 K

149 1 1351 1353 0 0 387.223 VNR

150 1 1354 1357 1 0 529.301 VHFK

151 1 1358 1368 1 0 1346.669 DFAEHFIPLM K

152 1 1369 1384 1 0 1748.826 ADLGDEFTPL AESAWK

153 1 1385 1385 1 0 146.106 K

154 1 1386 1399 0 0 1577.787 AFDVMIATIE QGQR

155 1 1400 1401 0 0 245.149 AR

156 1 1402 1402 0 0 174.112 R

157 1 1403 1413 0 0 1118.597 SVATFLTNPV A

158 2 1 4 1 0 473.260 AEVR

159 2 5 11 1 0 734.363 GILCSDK

160 2 12 15 1 0 431.274 ATIK

161 2 16 16 0 0 174.112 R

162 2 17 29 0 0 1490.752 TWSIVNDLPS FGR

163 2 30 43 2 0 1611.866 NVFLSVFAAK PEYK

164 2 44 50 0 0 923.486 NLFVEFR

165 2 51 62 0 0 1299.642 NIPASELANS ER

166 2 63 69 0 0 814.445 LLYHGGR

167 2 70 89 1 0 2058.037 VLASIDEVIS EIDSPDSAAK

168 2 90 90 1 0 146.106 K

169 2 91 97 0 0 756.449 LVALGER

170 2 98 101 0 0 525.302 HITR

171 2 102 105 0 0 431.249 GTVR

172 2 106 106 0 0 174.112 R

173 2 107 120 1 0 1715.819 HFEAFSYAFI DELK

174 2 121 122 0 0 302.170 QR

175 2 123 133 0 0 1115.572 GVASADLAAW R

176 2 134 134 1 0 146.106 K

177 2 135 149 1 0 1627.882 GWDSIVDILE AGLLK

178 2 150 150 0 0 174.112 R

179 2 151 179 1 0 3088.538 QIDLEVTGLS CVDVANVQES WATVSANLK

180 2 180 188 0 0 1034.551 NTGSILFQR

181 2 189 201 0 0 1671.837 LINDHPEYQQ LFR

182 2 202 204 0 0 449.239 QFR

183 2 205 210 1 0 673.365 DVELAK

184 2 211 223 0 0 1431.726 LGESNGFVAH VFR

185 2 224 233 1 0 1031.601 VVAAFDGIIK

186 2 234 246 1 0 1488.782 ELDNNPFIVS TLK

187 2 247 247 0 0 174.112 R

188 2 248 255 0 0 922.498 LGEQHIAR

189 2 256 266 0 0 1320.621 GTDISHFQNF R

190 2 267 288 1 0 2523.206 TTLLVYLNEN GMNQAQEASW NK

191 2 289 295 1 0 792.402 AFDAIEK

192 2 296 302 1 0 792.474 YISIGLK

193 2 303 306 0 0 431.249 SLGR

194 2 307 318 1 0 1227.671 VDPITGLSGL EK

195 2 319 327 1 0 1015.545 NAILNTWGK

196 2 328 329 0 0 273.180 VR

197 2 330 337 1 0 843.445 GNLQEVGK

198 2 338 342 1 0 522.280 ATFGK

199 2 343 355 0 0 1636.782 LFAAHPEYQQ MFR

200 2 356 370 1 0 1676.877 FFQGVQLAEL VDSPK

201 2 371 377 0 0 829.419 FAAHTQR

202 2 378 400 1 0 2592.398 VVSALDQTLL ALNRPSDFVY MIK

203 2 401 409 0 0 1065.557 ELGLDHINR

204 2 410 413 0 0 447.208 GTDR

205 2 414 428 1 0 1900.936 SHFENYQVVF VEYLK

206 2 429 441 1 0 1438.683 ETLGDSVDEF TVK

207 2 442 459 0 0 2147.116 SFNHVFEVII NFLNEGLR

208 2 460 473 0 0 1505.795 QANVVDPVTH LTGR

209 2 474 475 1 0 274.164 QK

210 2 476 479 1 0 459.269 EAIK

211 2 480 486 0 0 775.398 ASWSVAR

212 2 487 490 0 0 503.270 TDLR

213 2 491 499 0 0 1139.580 FLGQELFMR

214 2 500 514 1 0 1842.897 MFNLNPEYQS LFVNK

215 2 515 525 0 0 1172.655 GFADVPLVSL R

216 2 526 529 0 0 547.224 EDER

217 2 530 539 0 0 1186.628 FISHMANVLR

218 2 540 559 0 0 2350.148 GFDTLLQNLD DTSYFVYALR

219 2 560 568 0 0 1022.526 NLGDAHIQR

220 2 569 569 1 0 146.106 K

221 2 570 576 0 0 816.388 AGTEHFR

222 2 577 601 1 0 2734.385 SFEAILIPYL QESQGLDAAG VEAWK

223 2 602 616 1 0 1605.913 IFFDVSIGVI AQGLK

224 2 617 632 1 0 1621.783 VASSEEADPV TGLYGK

225 2 633 638 0 0 685.412 EVVALR

226 2 639 647 0 0 959.519 QAFAAISPR

227 2 648 653 1 0 658.365 NVEIGK

228 2 654 654 0 0 174.112 R

229 2 655 658 1 0 463.279 VFAK

230 2 659 667 1 0 1120.555 LFTSHPEYK

231 2 668 671 1 0 520.301 NLFK

232 2 672 672 1 0 146.106 K

233 2 673 697 0 0 2989.380 FEQYSVEELP STDAFDYHIS LVMNR

234 2 698 703 1 0 607.333 FSAVGK

235 2 704 716 1 0 1523.823 VIDDNVSFVY LLK

236 2 717 717 1 0 146.106 K

237 2 718 720 0 0 344.217 LGR

238 2 721 724 1 0 525.291 EHIK

239 2 725 725 0 0 174.112 R

240 2 726 729 0 0 431.249 GLSR

241 2 730 730 1 0 146.106 K

242 2 731 752 0 0 2570.254 QFDQFVELYI AEISPELSET GR

243 2 753 757 1 0 532.286 SGLEK

244 2 758 779 1 0 2349.258 VLTFATGVIE QGLFQLGQVD SK

245 2 780 786 1 0 744.438 ALTALEK

246 2 787 807 1 0 2383.274 QSIQDIWTSL RPTGLEELAV K

247 2 808 811 0 0 553.268 MFTR

248 2 812 820 1 0 1118.540 LFADHPEYK

249 2 821 826 0 0 705.417 LLFTGR

250 2 827 840 0 0 1571.769 LGNVDNINEN APFR

251 2 841 845 0 0 632.351 AHLHR

252 2 846 865 0 0 2186.231 VLSAFDIVIT SLDNNALLIR

253 2 866 868 1 0 387.248 QLK

254 2 869 876 0 0 957.503 DLGLFHTR

255 2 877 881 0 0 576.305 LGMTR

256 2 882 908 0 0 2935.487 AHFDNFATAF FSVAEDIVPN LLTALGR

257 2 909 913 1 0 532.286 ESLGK

258 2 914 916 1 0 350.195 GFK

259 2 917 931 0 0 1711.954 LMVAVIEEGL LQLER

260 2 932 941 0 0 1069.613 IDPITGLSVR

261 2 942 947 1 0 701.396 EVEVVK

262 2 948 963 1 0 1843.943 QTWNLVKPDL MGVGMR

263 2 964 966 1 0 406.258 IFK

264 2 967 971 1 0 622.333 SLFEK

265 2 972 981 1 0 1166.612 FPAYQAVFPK

266 2 982 989 1 0 919.465 FSDVPLDK

267 2 990 998 1 0 940.523 LEDIPAVGK

268 2 999 1006 1 0 855.481 HAISVTTK

269 2 1007 1025 0 0 2107.152 LDELIQTLDE PANLALLAR

270 2 1026 1042 2 0 1924.024 QLGEDHIVLG VNKPMFK

271 2 1043 1050 0 0 905.497 SFGEVLVR

272 2 1051 1059 0 0 1056.556 LLENDLGQR

273 2 1060 1066 1 0 799.386 FSNFASK

274 2 1067 1070 0 0 584.282 SWHR

275 2 1071 1088 1 0 2146.047 AYDVIVEYIE EGLQQSYK

276 2 1089 1100 1 0 1272.620 QDPVTGITDA EK

277 2 1101 1105 0 0 613.391 VLVQR

278 2 1106 1119 1 0 1595.903 SWELLKPDLL GLGR

279 2 1120 1120 1 0 146.106 K

280 2 1121 1128 1 0 923.548 IFGVIFTK

281 2 1129 1138 0 0 1302.672 HPEYQILFTR

282 2 1139 1161 1 0 2482.249 VGFGDTPLTQ LDNNPAFGEH IIK

283 2 1162 1164 0 0 404.221 VMR

284 2 1165 1171 0 0 882.460 AFDYVIR

285 2 1172 1177 2 0 655.402 NLGKPK

286 2 1178 1184 1 0 820.506 TLLAYLK

287 2 1185 1193 0 0 951.489 NVGADHIAR

288 2 1194 1197 0 0 516.266 NVER

289 2 1198 1198 0 0 174.112 R

290 2 1199 1213 0 0 1772.903 HFQAFSEALI PVMQR

291 2 1214 1216 1 0 388.232 ELK

292 2 1217 1228 1 0 1338.741 AQLKPEAVAA WR

293 2 1229 1229 1 0 146.106 K

294 2 1230 1233 0 0 459.244 GLDR

295 2 1234 1246 1 0 1337.828 IIGVIDQGLL GLK

296 2 1247 1264 1 0 1943.000 EVNPQIAFSA ADIEAIQK

297 2 1265 1276 2 0 1329.711 TWALAKPDLM GK

298 2 1277 1282 0 0 635.339 GASVFR

299 2 1283 1316 0 0 3860.864 QLFTDHGYQP LFSNLVEYEV TGLEGSPELN

THAR

300 2 1317 1337 1 0 2229.167 NVMAQLDTLV GSLQNSIELG K

301 2 1338 1344 1 0 758.429 SLNQLGK

302 2 1345 1349 0 0 622.319 DHVPR

303 2 1350 1350 1 0 146.106 K

304 2 1351 1353 1 0 359.217 VNK

305 2 1354 1368 1 0 1830.876 VHFDDFAEHF VPLMK

306 2 1369 1384 1 0 1747.842 ANLGDEFTPL AESAWK

307 2 1385 1385 1 0 146.106 K

308 2 1386 1399 0 0 1562.787 AFNVMVATIE QGQR

309 2 1400 1401 0 0 245.149 AR

310 2 1402 1402 0 0 174.112 R

311 2 1403 1413 0 0 1132.613 SIATFLTNPV A

sorted list of assignments

**************************

legend

d deviation experimentally determined mass - calculated mass

pid peptide id

om number of optional modifications

kl number of known links

ol number of optional links

sp number of subpeptides obtained after removing ALL links

i:m-n linear subpeptide extending from residue m to residue n of chain i

number of peaks assigned 8

number of peptides assigned 101

number of assignments 101

1 peak(s) with 1 peptide(s) assigned:

m(exp) m(calc) d[ppm] pid om kl ol sp residues

-------------------------------------------------------------------------------

927.400 927.446 49.9 101 0 0 1 2 1:914-916 2:914-916

1 peak(s) with 5 peptide(s) assigned:

m(exp) m(calc) d[ppm] pid om kl ol sp residues

-------------------------------------------------------------------------------

983.462 983.509 47.7 97 0 0 1 2 1:914-916 2:964-966

983.509 47.7 98 0 0 1 2 2:914-916 2:964-966

983.509 47.7 99 0 0 1 2 1:914-916 1:964-966

983.509 47.7 100 0 0 1 2 1:964-966 2:914-916

983.593 133.1 74 0 0 0 1 1:1121-1128

1 peak(s) with 6 peptide(s) assigned:

m(exp) m(calc) d[ppm] pid om kl ol sp residues

-------------------------------------------------------------------------------

1251.934 1251.622 249.3 91 0 0 1 2 1:1043-1046 1:1229-1233

1251.622 249.3 92 0 0 1 2 1:1043-1046 2:1229-1233

1251.647 229.2 46 0 0 1 2 1:698-703 1:866-868

1251.647 229.2 47 0 0 1 2 1:698-703 2:866-868

1251.658 220.2 93 0 0 1 2 1:12-16 1:1043-1046

1251.658 220.2 94 0 0 1 2 1:1043-1046 2:12-16

2 peak(s) with 7 peptide(s) assigned:

m(exp) m(calc) d[ppm] pid om kl ol sp residues

-------------------------------------------------------------------------------

1295.914 1295.659 196.5 6 1 0 0 1 1:247-255

1295.673 185.8 48 0 0 1 2 1:698-703 2:12-15

1295.673 185.8 49 0 0 1 2 1:12-15 1:698-703

1295.696 168.4 62 0 0 1 2 1:866-868 2:721-725

1295.696 168.4 63 0 0 1 2 2:721-725 2:866-868

1295.696 168.4 70 0 0 1 2 1:721-725 1:866-868

1295.696 168.4 71 0 0 1 2 1:721-725 2:866-868

1418.573 1418.691 83.5 52 0 0 1 2 1:284-288 1:1229-1233

1418.691 83.5 55 0 0 1 2 1:284-288 2:1229-1233

1418.728 109.1 53 0 0 1 2 1:12-16 1:284-288

1418.728 109.1 54 0 0 1 2 1:284-288 2:12-16

1418.728 109.1 56 0 0 1 2 1:480-486 2:866-868

1418.728 109.1 57 0 0 1 2 1:480-486 1:866-868

1418.732 112.0 61 0 0 1 2 1:1067-1070 1:1120-1124

1 peak(s) with 17 peptide(s) assigned:

m(exp) m(calc) d[ppm] pid om kl ol sp residues

-------------------------------------------------------------------------------

1273.996 1273.602 309.0 50 0 0 1 2 1:500-502 2:967-971

1273.627 289.4 2 0 0 0 1 2:1089-1100

1273.627 289.4 3 0 0 0 1 1:1089-1100

1273.639 280.6 1 0 0 0 1 1:51-62

1273.653 269.6 42 0 0 1 2 2:648-653 2:1214-1216

1273.653 269.6 44 0 0 1 2 1:648-653 2:1214-1216

1273.664 260.8 59 0 0 1 2 1:753-757 2:1350-1353

1273.664 260.8 89 0 0 1 2 1:1229-1233 2:476-479

1273.664 260.8 90 0 0 1 2 2:476-479 2:1229-1233

1273.700 232.2 87 0 0 1 2 1:12-16 2:476-479

1273.700 232.2 88 0 0 1 2 2:12-16 2:476-479

1273.711 223.4 95 0 0 1 2 1:726-730 2:1350-1353

1273.711 223.4 96 0 0 1 2 2:726-730 2:1350-1353

1274.627 494.8 66 0 0 1 2 2:338-342 2:721-724

1274.627 494.8 67 0 0 1 2 1:338-342 2:721-724

1274.627 494.8 72 0 0 1 2 1:721-724 2:338-342

1274.627 494.8 73 0 0 1 2 1:338-342 1:721-724

1 peak(s) with 20 peptide(s) assigned:

m(exp) m(calc) d[ppm] pid om kl ol sp residues

-------------------------------------------------------------------------------

1405.492 1405.664 122.2 51 0 0 1 2 1:1067-1070 2:967-971

1405.721 163.1 40 0 0 1 2 2:648-653 2:668-671

1405.721 163.1 41 0 0 1 2 1:668-671 2:648-653

1405.721 163.1 43 0 0 1 2 1:648-653 1:668-671

1405.721 163.1 45 0 0 1 2 1:648-653 2:668-671

1405.733 171.1 58 0 0 1 2 1:654-658 1:753-757

1405.733 171.1 60 0 0 1 2 1:753-757 2:654-658

1405.740 176.4 27 0 0 1 2 1:87-89 1:1036-1042

1405.758 189.0 80 0 0 1 2 1:655-658 2:474-479

1405.758 189.0 85 0 0 1 2 2:474-479 2:655-658

1405.769 197.0 75 0 0 1 2 1:474-475 1:1043-1050

1405.769 197.0 76 0 0 1 2 1:1043-1050 2:474-475

1405.780 205.0 77 0 0 1 2 1:654-658 1:726-730

1405.780 205.0 78 0 0 1 2 1:654-658 2:726-730

1405.780 205.0 79 0 0 1 2 1:655-658 1:725-730

1405.780 205.0 81 0 0 1 2 1:655-658 2:725-730

1405.780 205.0 82 0 0 1 2 1:726-730 2:654-658

1405.780 205.0 83 0 0 1 2 2:654-658 2:726-730

1405.780 205.0 84 0 0 1 2 1:725-730 2:655-658

1405.780 205.0 86 0 0 1 2 2:655-658 2:725-730

1 peak(s) with 38 peptide(s) assigned:

m(exp) m(calc) d[ppm] pid om kl ol sp residues

-------------------------------------------------------------------------------

1542.509 1541.760 485.9 34 0 0 1 2 1:1350-1353 2:1060-1066

1541.760 485.9 37 0 0 1 2 1:1067-1070 2:1338-1344

1541.806 455.9 28 0 0 1 2 2:476-479 2:999-1006

1541.806 455.9 29 0 0 1 2 1:999-1006 2:476-479

1541.810 453.3 35 0 0 1 2 2:296-302 2:338-342

1541.810 453.3 36 0 0 1 2 1:338-342 2:296-302

1541.833 438.7 64 0 0 1 2 1:655-658 2:718-724

1541.833 438.7 65 0 0 1 2 2:655-658 2:718-724

1541.833 438.7 68 0 0 1 2 1:655-658 1:718-724

1541.833 438.7 69 0 0 1 2 1:718-724 2:655-658

1542.790 182.2 4 0 0 1 2 1:866-868 1:990-998

1542.790 182.2 5 0 0 1 2 1:990-998 2:866-868

1542.813 196.8 30 0 0 1 2 1:717-720 2:330-337

1542.813 196.8 31 0 0 1 2 2:330-337 2:717-720

1542.813 196.8 32 0 0 1 2 1:330-337 2:717-720

1542.813 196.8 33 0 0 1 2 1:330-337 1:717-720

1542.874 236.6 7 0 0 1 2 1:87-97 2:717

1542.874 236.6 8 0 0 1 2 1:87-97 1:1350

1542.874 236.6 9 0 0 1 2 1:87-97 1:730

1542.874 236.6 10 0 0 1 2 1:87-97 1:672

1542.874 236.6 11 0 0 1 2 1:87-97 2:1385

1542.874 236.6 12 0 0 1 2 1:87-97 1:569

1542.874 236.6 13 0 0 1 2 1:87-97 2:569

1542.874 236.6 14 0 0 1 2 1:87-97 2:134

1542.874 236.6 15 0 0 1 2 1:87-97 1:602

1542.874 236.6 16 0 0 1 2 1:87-97 1:1120

1542.874 236.6 17 0 0 1 2 1:87-97 1:1229

1542.874 236.6 18 0 0 1 2 1:87-97 1:1385

1542.874 236.6 19 0 0 1 2 1:87-97 2:1229

1542.874 236.6 20 0 0 1 2 1:87-97 2:90

1542.874 236.6 21 0 0 1 2 1:87-97 1:717

1542.874 236.6 22 0 0 1 2 1:87-97 2:1120

1542.874 236.6 23 0 0 1 2 1:87-97 2:730

1542.874 236.6 24 0 0 1 2 1:87-97 2:1350

1542.874 236.6 25 0 0 1 2 1:87-97 1:247

1542.874 236.6 26 0 0 1 2 1:87-97 2:672

1542.874 236.6 38 0 0 1 2 1:12-15 2:90-97

1542.874 236.6 39 0 0 1 2 2:12-15 2:90-97

peptide isomers

***************

legend

[ n-terminal NH2 group

[[ n-terminal NH2 group (side chain blocked)

] c-terminal COOH group

]] c-terminal COOH group (side chain blocked)

k known cross-link/modification

o optional cross-link/modification

L left side of a cross-linker

R right side of a cross-linker

x cross-linker (left or right side)

~ missed cleavage

= missed cleavage (mandatory)

legend for detailed list of psd assignments

c intensity class of assigned psd peak (i=intense,w=weak)

*******************************************************************************

peptide 1

number of base peptides = 1

number of missed cleavages = 0

number of optional links = 0

number of known links = 0

number of optional modifications = 0

number of known modifications = 0

calculated mass = 1273.6387

...............................................................................

isomer 1 / peptide 1

1N51--1R62

. . . . . . . . . . . . . . . . . . . . . . . . . . . . . . . . . . . . . . . .

psd analysis for psd peak list "7" (selected parent mass = 1273.996)

concise lists of psd assignments

simple chain fragmentation

chain 1 1

----- - -

5 6

1 2

rule N I P A S E L A S S E R score

a 0.0

b 0.0

b' 0.0

b" 0.0

b-NH3 0.0

b-H2O 5 6 11 8.0

b(n-1)+H2O 0.0

y 8 4 4.0

y' 0.0

y" 0.0

y-NH3 10 2.0

y-H2O 10 2.0

detailed list of psd assignments

number of unassigned psd peaks 21

number of psd peaks assigned 6

number of psd fragments assigned 7

number of psd assignments 7

total score 16.0

m(exp) m(calc) d[Da] score psd rule fragmentation site(s)

-------------------------------------------------------------------------------

464.500 465.246 0.7 2.0 b-H2O5 [1S55]-1E56

479.500 478.226 1.3 2.0 y4 1A58-[1S59]

593.200 594.289 1.1 4.0 b-H2O6 [1E56]-1L57

877.800 878.422 0.6 2.0 y8 1A54-[1S55]

1027.500 1028.501 1.0 2.0 y-H2O10 1I52-[1P53]

1027.500 1029.485 2.0 2.0 y-NH310 1I52-[1P53]

1079.933 1081.516 1.6 2.0 b-H2O11 [1E61]-1R62

*** legend ********************************************************************

peptide 2

number of base peptides = 1

number of missed cleavages = 0

number of optional links = 0

number of known links = 0

number of optional modifications = 0

number of known modifications = 0

calculated mass = 1273.6274

...............................................................................

isomer 1 / peptide 2

2Q1089--2K1100

. . . . . . . . . . . . . . . . . . . . . . . . . . . . . . . . . . . . . . . .

psd analysis for psd peak list "7" (selected parent mass = 1273.996)

concise lists of psd assignments

simple chain fragmentation

chain 2 2

----- - -

1 1

0 1

8 0

9 0

rule Q D P V T G I T D A E K score

a 0.0

b 0.0

b' 0.0

b" 0.0

b-NH3 0.0

b-H2O 0.0

b(n-1)+H2O 0.0

y 0.0

y' 0.0

y" 0.0

y-NH3 0.0

y-H2O 8 6 4.0

detailed list of psd assignments

number of unassigned psd peaks 25

number of psd peaks assigned 2

number of psd fragments assigned 2

number of psd assignments 2

total score 4.0

m(exp) m(calc) d[Da] score psd rule fragmentation site(s)

-------------------------------------------------------------------------------

656.667 658.341 1.7 2.0 y-H2O6 2G1094-[2I1095]

815.667 816.410 0.7 2.0 y-H2O8 2V1092-[2T1093]

*** legend ********************************************************************

peptide 3

number of base peptides = 1

number of missed cleavages = 0

number of optional links = 0

number of known links = 0

number of optional modifications = 0

number of known modifications = 0

calculated mass = 1273.6274

...............................................................................

isomer 1 / peptide 3

1Q1089--1K1100

. . . . . . . . . . . . . . . . . . . . . . . . . . . . . . . . . . . . . . . .

psd analysis for psd peak list "7" (selected parent mass = 1273.996)

concise lists of psd assignments

simple chain fragmentation

chain 1 1

----- - -

1 1

0 1

8 0

9 0

rule Q D P V T G I T D A E K score

a 0.0

b 0.0

b' 0.0

b" 0.0

b-NH3 0.0

b-H2O 0.0

b(n-1)+H2O 0.0

y 0.0

y' 0.0

y" 0.0

y-NH3 0.0

y-H2O 8 6 4.0

detailed list of psd assignments

number of unassigned psd peaks 25

number of psd peaks assigned 2

number of psd fragments assigned 2

number of psd assignments 2

total score 4.0

m(exp) m(calc) d[Da] score psd rule fragmentation site(s)

-------------------------------------------------------------------------------

656.667 658.341 1.7 2.0 y-H2O6 1G1094-[1I1095]

815.667 816.410 0.7 2.0 y-H2O8 1V1092-[1T1093]

*** legend ********************************************************************

peptide 4

number of base peptides = 2

number of missed cleavages = 0

number of optional links = 1

number of known links = 0

number of optional modifications = 0

number of known modifications = 0

calculated mass = 1542.7901

...............................................................................

isomer 1 / peptide 4

oooooooooooooooo

1Q866--1K868 1L990--1K998

. . . . . . . . . . . . . . . . . . . . . . . . . . . . . . . . . . . . . . . .

psd analysis for psd peak list "2" (selected parent mass = 1542.509)

concise lists of psd assignments

simple chain fragmentation

chain 1 1 1 1

----- - - - -

8 8 9 9

6 6 9 9

6 8 0 8

rule Q L K L E D T P A V G K score

a 0.0

b 0.0

b' 0.0

b" 0.0

b-NH3 0.0

b-H2O 0.0

b(n-1)+H2O 0.0

y 7 3 4.0

y' 7 1.5

y" 0.0

y-NH3 0.0

y-H2O 2 8 6 6.0

linker fragmentation

link rule link score matching psd rule(s)

-------------------------------------------------------------------------------

egs 1K868-1K998 3.0 regs11,regs15

detailed list of psd assignments

number of unassigned psd peaks 39

number of psd peaks assigned 7

number of psd fragments assigned 8

number of psd assignments 8

total score 14.5

m(exp) m(calc) d[Da] score psd rule fragmentation site(s)

-------------------------------------------------------------------------------

915.000 916.499 1.5 2.0 y3 1A995-[1V996]

1044.500 1042.518 2.0 1.5 regs11 1K868-[1K998]

1113.867 1114.539 0.7 1.5 regs15 1K868-[1K998]

1167.000 1167.626 0.6 2.0 y-H2O6 1D992-[1T993]

1298.667 1300.663 2.0 2.0 y7 1E991-[1D992]

1298.667 1300.663 2.0 1.5 y'7 1E991-[1D992]

1397.467 1396.721 0.7 2.0 y-H2O2 1Q866-[1L867]

1411.400 1411.695 0.3 2.0 y-H2O8 1L990-[1E991]

*** legend ********************************************************************

peptide 5

number of base peptides = 2

number of missed cleavages = 0

number of optional links = 1

number of known links = 0

number of optional modifications = 0

number of known modifications = 0

calculated mass = 1542.7901

...............................................................................

isomer 1 / peptide 5

oooooooooooooooo

1L990--1K998 2Q866--2K868

. . . . . . . . . . . . . . . . . . . . . . . . . . . . . . . . . . . . . . . .

psd analysis for psd peak list "2" (selected parent mass = 1542.509)

concise lists of psd assignments

simple chain fragmentation

chain 1 1 2 2

----- - - - -

9 9 8 8

9 9 6 6

0 8 6 8

rule L E D T P A V G K Q L K score

a 0.0

b 0.0

b' 0.0

b" 0.0

b-NH3 0.0

b-H2O 0.0

b(n-1)+H2O 0.0

y 7 3 4.0

y' 7 1.5

y" 0.0

y-NH3 0.0

y-H2O 8 6 2 6.0

linker fragmentation

link rule link score matching psd rule(s)

-------------------------------------------------------------------------------

egs 1K998-2K868 3.0 legs11,legs15

detailed list of psd assignments

number of unassigned psd peaks 39

number of psd peaks assigned 7

number of psd fragments assigned 8

number of psd assignments 8

total score 14.5

m(exp) m(calc) d[Da] score psd rule fragmentation site(s)

-------------------------------------------------------------------------------

915.000 916.499 1.5 2.0 y3 1A995-[1V996]

1044.500 1042.518 2.0 1.5 legs11 [1K998]-2K868

1113.867 1114.539 0.7 1.5 legs15 [1K998]-2K868

1167.000 1167.626 0.6 2.0 y-H2O6 1D992-[1T993]

1298.667 1300.663 2.0 2.0 y7 1E991-[1D992]

1298.667 1300.663 2.0 1.5 y'7 1E991-[1D992]

1397.467 1396.721 0.7 2.0 y-H2O2 2Q866-[2L867]

1411.400 1411.695 0.3 2.0 y-H2O8 1L990-[1E991]

*** legend ********************************************************************

peptide 6

number of base peptides = 2

number of missed cleavages = 1

number of optional links = 0

number of known links = 0

number of optional modifications = 1

number of known modifications = 0

calculated mass = 1295.6594

...............................................................................

isomer 1 / peptide 6

1K247 ~ 1L248--1R255

o

x

. . . . . . . . . . . . . . . . . . . . . . . . . . . . . . . . . . . . . . . .

psd analysis for psd peak list "5" (selected parent mass = 1295.914)

concise lists of psd assignments

simple chain fragmentation

chain 1 1

----- - -

2 2

4 5

7 5

rule K L G E Q H I A R score

a 0.0

b 3 7 4.0

b' 0.0

b" 0.0

b-NH3 0.0

b-H2O 0.0

b(n-1)+H2O 0.0

y 3 2.0

y' 0.0

y" 0.0

y-NH3 0.0

y-H2O 0.0

detailed list of psd assignments

number of unassigned psd peaks 19

number of psd peaks assigned 4

number of psd fragments assigned 3

number of psd assignments 4

total score 6.0

m(exp) m(calc) d[Da] score psd rule fragmentation site(s)

-------------------------------------------------------------------------------

357.733 359.241 1.5 2.0 y3 1H252-[1I253]

359.733 359.241 0.5 2.0 y3 1H252-[1I253]

542.733 543.267 0.5 2.0 b3 [1G249]-1E250

1049.000 1050.511 1.5 2.0 b7 [1I253]-1A254

*** legend ********************************************************************

peptide 7

number of base peptides = 3

number of missed cleavages = 1

number of optional links = 1

number of known links = 0

number of optional modifications = 0

number of known modifications = 0

calculated mass = 1542.8741

...............................................................................

isomer 1 / peptide 7

ooooooooooooooooooooo

1A87--1K89 ~ 1T90--1R97 2K717

. . . . . . . . . . . . . . . . . . . . . . . . . . . . . . . . . . . . . . . .

psd analysis for psd peak list "2" (selected parent mass = 1542.509)

concise lists of psd assignments

simple chain fragmentation

chain 1 1 2

----- - - -

0 0 7

8 9 1

7 7 7

rule A V K T L L A L G E R K score

a 0.0

b 0.0

b' 0.0

b" 0.0

b-NH3 0.0

b-H2O 0.0

b(n-1)+H2O 0.0

y 0.0

y' 0.0

y" 0.0

y-NH3 9 2.0

y-H2O 9 2.0

linker fragmentation

link rule link score matching psd rule(s)

-------------------------------------------------------------------------------

egs 1K89-2K717 6.0 legs12,legs14,legs15,legs17

detailed list of psd assignments

number of unassigned psd peaks 40

number of psd peaks assigned 6

number of psd fragments assigned 6

number of psd assignments 9

total score 10.0

m(exp) m(calc) d[Da] score psd rule fragmentation site(s)

-------------------------------------------------------------------------------

1298.667 1297.760 0.9 1.5 legs12 [1K89]-2K717

1339.500 1340.742 1.2 1.5 legs14 [1K89]-2K717

1341.000 1340.742 0.3 1.5 legs14 [1K89]-2K717

1355.533 1354.758 0.8 2.0 y-H2O9 1V88-[1K89]

1355.533 1355.742 0.2 2.0 y-NH39 1V88-[1K89]

1355.533 1355.766 0.2 1.5 legs15 [1K89]-2K717

1357.267 1355.742 1.5 2.0 y-NH39 1V88-[1K89]

1357.267 1355.766 1.5 1.5 legs15 [1K89]-2K717

1397.467 1396.769 0.7 1.5 legs17 [1K89]-2K717

*** legend ********************************************************************

peptide 8

number of base peptides = 3

number of missed cleavages = 1

number of optional links = 1

number of known links = 0

number of optional modifications = 0

number of known modifications = 0

calculated mass = 1542.8741

...............................................................................

isomer 1 / peptide 8

oooooooooooooooooooooo

1A87--1K89 ~ 1T90--1R97 1K1350

. . . . . . . . . . . . . . . . . . . . . . . . . . . . . . . . . . . . . . . .

psd analysis for psd peak list "2" (selected parent mass = 1542.509)

concise lists of psd assignments

simple chain fragmentation

chain 1 1 1

----- - - -

0 0 1

0 0 3

8 9 5

7 7 0

rule A V K T L L A L G E R K score

a 0.0

b 0.0

b' 0.0

b" 0.0

b-NH3 0.0

b-H2O 0.0

b(n-1)+H2O 0.0

y 0.0

y' 0.0

y" 0.0

y-NH3 9 2.0

y-H2O 9 2.0

linker fragmentation

link rule link score matching psd rule(s)

-------------------------------------------------------------------------------

egs 1K89-1K1350 6.0 legs12,legs14,legs15,legs17

detailed list of psd assignments

number of unassigned psd peaks 40

number of psd peaks assigned 6

number of psd fragments assigned 6

number of psd assignments 9

total score 10.0

m(exp) m(calc) d[Da] score psd rule fragmentation site(s)

-------------------------------------------------------------------------------

1298.667 1297.760 0.9 1.5 legs12 [1K89]-1K1350

1339.500 1340.742 1.2 1.5 legs14 [1K89]-1K1350

1341.000 1340.742 0.3 1.5 legs14 [1K89]-1K1350

1355.533 1354.758 0.8 2.0 y-H2O9 1V88-[1K89]

1355.533 1355.742 0.2 2.0 y-NH39 1V88-[1K89]

1355.533 1355.766 0.2 1.5 legs15 [1K89]-1K1350

1357.267 1355.742 1.5 2.0 y-NH39 1V88-[1K89]

1357.267 1355.766 1.5 1.5 legs15 [1K89]-1K1350

1397.467 1396.769 0.7 1.5 legs17 [1K89]-1K1350

*** legend ********************************************************************

peptide 9

number of base peptides = 3

number of missed cleavages = 1

number of optional links = 1

number of known links = 0

number of optional modifications = 0

number of known modifications = 0

calculated mass = 1542.8741

...............................................................................

isomer 1 / peptide 9

ooooooooooooooooooooo

1A87--1K89 ~ 1T90--1R97 1K730

. . . . . . . . . . . . . . . . . . . . . . . . . . . . . . . . . . . . . . . .

psd analysis for psd peak list "2" (selected parent mass = 1542.509)

concise lists of psd assignments

simple chain fragmentation

chain 1 1 1

----- - - -

0 0 7

8 9 3

7 7 0

rule A V K T L L A L G E R K score

a 0.0

b 0.0

b' 0.0

b" 0.0

b-NH3 0.0

b-H2O 0.0

b(n-1)+H2O 0.0

y 0.0

y' 0.0

y" 0.0

y-NH3 9 2.0

y-H2O 9 2.0

linker fragmentation

link rule link score matching psd rule(s)

-------------------------------------------------------------------------------

egs 1K89-1K730 6.0 legs12,legs14,legs15,legs17

detailed list of psd assignments

number of unassigned psd peaks 40

number of psd peaks assigned 6

number of psd fragments assigned 6

number of psd assignments 9

total score 10.0

m(exp) m(calc) d[Da] score psd rule fragmentation site(s)

-------------------------------------------------------------------------------

1298.667 1297.760 0.9 1.5 legs12 [1K89]-1K730

1339.500 1340.742 1.2 1.5 legs14 [1K89]-1K730

1341.000 1340.742 0.3 1.5 legs14 [1K89]-1K730

1355.533 1354.758 0.8 2.0 y-H2O9 1V88-[1K89]

1355.533 1355.742 0.2 2.0 y-NH39 1V88-[1K89]

1355.533 1355.766 0.2 1.5 legs15 [1K89]-1K730

1357.267 1355.742 1.5 2.0 y-NH39 1V88-[1K89]

1357.267 1355.766 1.5 1.5 legs15 [1K89]-1K730

1397.467 1396.769 0.7 1.5 legs17 [1K89]-1K730

*** legend ********************************************************************

peptide 10

number of base peptides = 3

number of missed cleavages = 1

number of optional links = 1

number of known links = 0

number of optional modifications = 0

number of known modifications = 0

calculated mass = 1542.8741

...............................................................................

isomer 1 / peptide 10

ooooooooooooooooooooo

1A87--1K89 ~ 1T90--1R97 1K672

. . . . . . . . . . . . . . . . . . . . . . . . . . . . . . . . . . . . . . . .

psd analysis for psd peak list "2" (selected parent mass = 1542.509)

concise lists of psd assignments

simple chain fragmentation

chain 1 1 1

----- - - -

0 0 6

8 9 7

7 7 2

rule A V K T L L A L G E R K score

a 0.0

b 0.0

b' 0.0

b" 0.0

b-NH3 0.0

b-H2O 0.0

b(n-1)+H2O 0.0

y 0.0

y' 0.0

y" 0.0

y-NH3 9 2.0

y-H2O 9 2.0

linker fragmentation

link rule link score matching psd rule(s)

-------------------------------------------------------------------------------

egs 1K89-1K672 6.0 legs12,legs14,legs15,legs17

detailed list of psd assignments

number of unassigned psd peaks 40

number of psd peaks assigned 6

number of psd fragments assigned 6

number of psd assignments 9

total score 10.0

m(exp) m(calc) d[Da] score psd rule fragmentation site(s)

-------------------------------------------------------------------------------

1298.667 1297.760 0.9 1.5 legs12 [1K89]-1K672

1339.500 1340.742 1.2 1.5 legs14 [1K89]-1K672

1341.000 1340.742 0.3 1.5 legs14 [1K89]-1K672

1355.533 1354.758 0.8 2.0 y-H2O9 1V88-[1K89]

1355.533 1355.742 0.2 2.0 y-NH39 1V88-[1K89]

1355.533 1355.766 0.2 1.5 legs15 [1K89]-1K672

1357.267 1355.742 1.5 2.0 y-NH39 1V88-[1K89]

1357.267 1355.766 1.5 1.5 legs15 [1K89]-1K672

1397.467 1396.769 0.7 1.5 legs17 [1K89]-1K672

*** legend ********************************************************************

peptide 11

number of base peptides = 3

number of missed cleavages = 1

number of optional links = 1

number of known links = 0

number of optional modifications = 0

number of known modifications = 0

calculated mass = 1542.8741

...............................................................................

isomer 1 / peptide 11

oooooooooooooooooooooo

1A87--1K89 ~ 1T90--1R97 2K1385

. . . . . . . . . . . . . . . . . . . . . . . . . . . . . . . . . . . . . . . .

psd analysis for psd peak list "2" (selected parent mass = 1542.509)

concise lists of psd assignments

simple chain fragmentation

chain 1 1 2

----- - - -

0 0 1

0 0 3

8 9 8

7 7 5

rule A V K T L L A L G E R K score

a 0.0

b 0.0

b' 0.0

b" 0.0

b-NH3 0.0

b-H2O 0.0

b(n-1)+H2O 0.0

y 0.0

y' 0.0

y" 0.0

y-NH3 9 2.0

y-H2O 9 2.0

linker fragmentation

link rule link score matching psd rule(s)

-------------------------------------------------------------------------------

egs 1K89-2K1385 6.0 legs12,legs14,legs15,legs17

detailed list of psd assignments

number of unassigned psd peaks 40

number of psd peaks assigned 6

number of psd fragments assigned 6

number of psd assignments 9

total score 10.0

m(exp) m(calc) d[Da] score psd rule fragmentation site(s)

-------------------------------------------------------------------------------

1298.667 1297.760 0.9 1.5 legs12 [1K89]-2K1385

1339.500 1340.742 1.2 1.5 legs14 [1K89]-2K1385

1341.000 1340.742 0.3 1.5 legs14 [1K89]-2K1385

1355.533 1354.758 0.8 2.0 y-H2O9 1V88-[1K89]

1355.533 1355.742 0.2 2.0 y-NH39 1V88-[1K89]

1355.533 1355.766 0.2 1.5 legs15 [1K89]-2K1385

1357.267 1355.742 1.5 2.0 y-NH39 1V88-[1K89]

1357.267 1355.766 1.5 1.5 legs15 [1K89]-2K1385

1397.467 1396.769 0.7 1.5 legs17 [1K89]-2K1385

*** legend ********************************************************************

peptide 12

number of base peptides = 3

number of missed cleavages = 1

number of optional links = 1

number of known links = 0

number of optional modifications = 0

number of known modifications = 0

calculated mass = 1542.8741

...............................................................................

isomer 1 / peptide 12

ooooooooooooooooooooo

1A87--1K89 ~ 1T90--1R97 1K569

. . . . . . . . . . . . . . . . . . . . . . . . . . . . . . . . . . . . . . . .

psd analysis for psd peak list "2" (selected parent mass = 1542.509)

concise lists of psd assignments

simple chain fragmentation

chain 1 1 1

----- - - -

0 0 5

8 9 6

7 7 9

rule A V K T L L A L G E R K score

a 0.0

b 0.0

b' 0.0

b" 0.0

b-NH3 0.0

b-H2O 0.0

b(n-1)+H2O 0.0

y 0.0

y' 0.0

y" 0.0

y-NH3 9 2.0

y-H2O 9 2.0

linker fragmentation

link rule link score matching psd rule(s)

-------------------------------------------------------------------------------

egs 1K89-1K569 6.0 legs12,legs14,legs15,legs17

detailed list of psd assignments

number of unassigned psd peaks 40

number of psd peaks assigned 6

number of psd fragments assigned 6

number of psd assignments 9

total score 10.0

m(exp) m(calc) d[Da] score psd rule fragmentation site(s)

-------------------------------------------------------------------------------

1298.667 1297.760 0.9 1.5 legs12 [1K89]-1K569

1339.500 1340.742 1.2 1.5 legs14 [1K89]-1K569

1341.000 1340.742 0.3 1.5 legs14 [1K89]-1K569

1355.533 1354.758 0.8 2.0 y-H2O9 1V88-[1K89]

1355.533 1355.742 0.2 2.0 y-NH39 1V88-[1K89]

1355.533 1355.766 0.2 1.5 legs15 [1K89]-1K569

1357.267 1355.742 1.5 2.0 y-NH39 1V88-[1K89]

1357.267 1355.766 1.5 1.5 legs15 [1K89]-1K569

1397.467 1396.769 0.7 1.5 legs17 [1K89]-1K569

*** legend ********************************************************************

peptide 13

number of base peptides = 3

number of missed cleavages = 1

number of optional links = 1

number of known links = 0

number of optional modifications = 0

number of known modifications = 0

calculated mass = 1542.8741

...............................................................................

isomer 1 / peptide 13

ooooooooooooooooooooo

1A87--1K89 ~ 1T90--1R97 2K569

. . . . . . . . . . . . . . . . . . . . . . . . . . . . . . . . . . . . . . . .

psd analysis for psd peak list "2" (selected parent mass = 1542.509)

concise lists of psd assignments

simple chain fragmentation

chain 1 1 2

----- - - -

0 0 5

8 9 6

7 7 9

rule A V K T L L A L G E R K score

a 0.0

b 0.0

b' 0.0

b" 0.0

b-NH3 0.0

b-H2O 0.0

b(n-1)+H2O 0.0

y 0.0

y' 0.0

y" 0.0

y-NH3 9 2.0

y-H2O 9 2.0

linker fragmentation

link rule link score matching psd rule(s)

-------------------------------------------------------------------------------

egs 1K89-2K569 6.0 legs12,legs14,legs15,legs17

detailed list of psd assignments

number of unassigned psd peaks 40

number of psd peaks assigned 6

number of psd fragments assigned 6

number of psd assignments 9

total score 10.0

m(exp) m(calc) d[Da] score psd rule fragmentation site(s)

-------------------------------------------------------------------------------

1298.667 1297.760 0.9 1.5 legs12 [1K89]-2K569

1339.500 1340.742 1.2 1.5 legs14 [1K89]-2K569

1341.000 1340.742 0.3 1.5 legs14 [1K89]-2K569

1355.533 1354.758 0.8 2.0 y-H2O9 1V88-[1K89]

1355.533 1355.742 0.2 2.0 y-NH39 1V88-[1K89]

1355.533 1355.766 0.2 1.5 legs15 [1K89]-2K569

1357.267 1355.742 1.5 2.0 y-NH39 1V88-[1K89]

1357.267 1355.766 1.5 1.5 legs15 [1K89]-2K569

1397.467 1396.769 0.7 1.5 legs17 [1K89]-2K569

*** legend ********************************************************************

peptide 14

number of base peptides = 3

number of missed cleavages = 1

number of optional links = 1

number of known links = 0

number of optional modifications = 0

number of known modifications = 0

calculated mass = 1542.8741

...............................................................................

isomer 1 / peptide 14

ooooooooooooooooooooo

1A87--1K89 ~ 1T90--1R97 2K134

. . . . . . . . . . . . . . . . . . . . . . . . . . . . . . . . . . . . . . . .

psd analysis for psd peak list "2" (selected parent mass = 1542.509)

concise lists of psd assignments

simple chain fragmentation

chain 1 1 2

----- - - -

0 0 1

8 9 3

7 7 4

rule A V K T L L A L G E R K score

a 0.0

b 0.0

b' 0.0

b" 0.0

b-NH3 0.0

b-H2O 0.0

b(n-1)+H2O 0.0

y 0.0

y' 0.0

y" 0.0

y-NH3 9 2.0

y-H2O 9 2.0

linker fragmentation

link rule link score matching psd rule(s)

-------------------------------------------------------------------------------

egs 1K89-2K134 6.0 legs12,legs14,legs15,legs17

detailed list of psd assignments

number of unassigned psd peaks 40

number of psd peaks assigned 6

number of psd fragments assigned 6

number of psd assignments 9

total score 10.0

m(exp) m(calc) d[Da] score psd rule fragmentation site(s)

-------------------------------------------------------------------------------

1298.667 1297.760 0.9 1.5 legs12 [1K89]-2K134

1339.500 1340.742 1.2 1.5 legs14 [1K89]-2K134

1341.000 1340.742 0.3 1.5 legs14 [1K89]-2K134

1355.533 1354.758 0.8 2.0 y-H2O9 1V88-[1K89]

1355.533 1355.742 0.2 2.0 y-NH39 1V88-[1K89]

1355.533 1355.766 0.2 1.5 legs15 [1K89]-2K134

1357.267 1355.742 1.5 2.0 y-NH39 1V88-[1K89]

1357.267 1355.766 1.5 1.5 legs15 [1K89]-2K134

1397.467 1396.769 0.7 1.5 legs17 [1K89]-2K134

*** legend ********************************************************************

peptide 15

number of base peptides = 3

number of missed cleavages = 1

number of optional links = 1

number of known links = 0

number of optional modifications = 0

number of known modifications = 0

calculated mass = 1542.8741

...............................................................................

isomer 1 / peptide 15

ooooooooooooooooooooo

1A87--1K89 ~ 1T90--1R97 1K602

. . . . . . . . . . . . . . . . . . . . . . . . . . . . . . . . . . . . . . . .

psd analysis for psd peak list "2" (selected parent mass = 1542.509)

concise lists of psd assignments

simple chain fragmentation

chain 1 1 1

----- - - -

0 0 6

8 9 0

7 7 2

rule A V K T L L A L G E R K score

a 0.0

b 0.0

b' 0.0

b" 0.0

b-NH3 0.0

b-H2O 0.0

b(n-1)+H2O 0.0

y 0.0

y' 0.0

y" 0.0

y-NH3 9 2.0

y-H2O 9 2.0

linker fragmentation

link rule link score matching psd rule(s)

-------------------------------------------------------------------------------

egs 1K89-1K602 6.0 legs12,legs14,legs15,legs17

detailed list of psd assignments

number of unassigned psd peaks 40

number of psd peaks assigned 6

number of psd fragments assigned 6

number of psd assignments 9

total score 10.0

m(exp) m(calc) d[Da] score psd rule fragmentation site(s)

-------------------------------------------------------------------------------

1298.667 1297.760 0.9 1.5 legs12 [1K89]-1K602

1339.500 1340.742 1.2 1.5 legs14 [1K89]-1K602

1341.000 1340.742 0.3 1.5 legs14 [1K89]-1K602

1355.533 1354.758 0.8 2.0 y-H2O9 1V88-[1K89]

1355.533 1355.742 0.2 2.0 y-NH39 1V88-[1K89]

1355.533 1355.766 0.2 1.5 legs15 [1K89]-1K602

1357.267 1355.742 1.5 2.0 y-NH39 1V88-[1K89]

1357.267 1355.766 1.5 1.5 legs15 [1K89]-1K602

1397.467 1396.769 0.7 1.5 legs17 [1K89]-1K602

*** legend ********************************************************************

peptide 16

number of base peptides = 3

number of missed cleavages = 1

number of optional links = 1

number of known links = 0

number of optional modifications = 0

number of known modifications = 0

calculated mass = 1542.8741

...............................................................................

isomer 1 / peptide 16

oooooooooooooooooooooo

1A87--1K89 ~ 1T90--1R97 1K1120

. . . . . . . . . . . . . . . . . . . . . . . . . . . . . . . . . . . . . . . .

psd analysis for psd peak list "2" (selected parent mass = 1542.509)

concise lists of psd assignments

simple chain fragmentation

chain 1 1 1

----- - - -

0 0 1

0 0 1

8 9 2

7 7 0

rule A V K T L L A L G E R K score

a 0.0

b 0.0

b' 0.0

b" 0.0

b-NH3 0.0

b-H2O 0.0

b(n-1)+H2O 0.0

y 0.0

y' 0.0

y" 0.0

y-NH3 9 2.0

y-H2O 9 2.0

linker fragmentation

link rule link score matching psd rule(s)

-------------------------------------------------------------------------------

egs 1K89-1K1120 6.0 legs12,legs14,legs15,legs17

detailed list of psd assignments

number of unassigned psd peaks 40

number of psd peaks assigned 6

number of psd fragments assigned 6

number of psd assignments 9

total score 10.0

m(exp) m(calc) d[Da] score psd rule fragmentation site(s)

-------------------------------------------------------------------------------

1298.667 1297.760 0.9 1.5 legs12 [1K89]-1K1120

1339.500 1340.742 1.2 1.5 legs14 [1K89]-1K1120

1341.000 1340.742 0.3 1.5 legs14 [1K89]-1K1120

1355.533 1354.758 0.8 2.0 y-H2O9 1V88-[1K89]

1355.533 1355.742 0.2 2.0 y-NH39 1V88-[1K89]

1355.533 1355.766 0.2 1.5 legs15 [1K89]-1K1120

1357.267 1355.742 1.5 2.0 y-NH39 1V88-[1K89]

1357.267 1355.766 1.5 1.5 legs15 [1K89]-1K1120

1397.467 1396.769 0.7 1.5 legs17 [1K89]-1K1120

*** legend ********************************************************************

peptide 17

number of base peptides = 3

number of missed cleavages = 1

number of optional links = 1

number of known links = 0

number of optional modifications = 0

number of known modifications = 0

calculated mass = 1542.8741

...............................................................................

isomer 1 / peptide 17

oooooooooooooooooooooo

1A87--1K89 ~ 1T90--1R97 1K1229

. . . . . . . . . . . . . . . . . . . . . . . . . . . . . . . . . . . . . . . .

psd analysis for psd peak list "2" (selected parent mass = 1542.509)

concise lists of psd assignments

simple chain fragmentation

chain 1 1 1

----- - - -

0 0 1

0 0 2

8 9 2

7 7 9

rule A V K T L L A L G E R K score

a 0.0

b 0.0

b' 0.0

b" 0.0

b-NH3 0.0

b-H2O 0.0

b(n-1)+H2O 0.0

y 0.0

y' 0.0

y" 0.0

y-NH3 9 2.0

y-H2O 9 2.0

linker fragmentation

link rule link score matching psd rule(s)

-------------------------------------------------------------------------------

egs 1K89-1K1229 6.0 legs12,legs14,legs15,legs17

detailed list of psd assignments

number of unassigned psd peaks 40

number of psd peaks assigned 6

number of psd fragments assigned 6

number of psd assignments 9

total score 10.0

m(exp) m(calc) d[Da] score psd rule fragmentation site(s)

-------------------------------------------------------------------------------

1298.667 1297.760 0.9 1.5 legs12 [1K89]-1K1229

1339.500 1340.742 1.2 1.5 legs14 [1K89]-1K1229

1341.000 1340.742 0.3 1.5 legs14 [1K89]-1K1229

1355.533 1354.758 0.8 2.0 y-H2O9 1V88-[1K89]

1355.533 1355.742 0.2 2.0 y-NH39 1V88-[1K89]

1355.533 1355.766 0.2 1.5 legs15 [1K89]-1K1229

1357.267 1355.742 1.5 2.0 y-NH39 1V88-[1K89]

1357.267 1355.766 1.5 1.5 legs15 [1K89]-1K1229

1397.467 1396.769 0.7 1.5 legs17 [1K89]-1K1229

*** legend ********************************************************************

peptide 18

number of base peptides = 3

number of missed cleavages = 1

number of optional links = 1

number of known links = 0

number of optional modifications = 0

number of known modifications = 0

calculated mass = 1542.8741

...............................................................................

isomer 1 / peptide 18

oooooooooooooooooooooo

1A87--1K89 ~ 1T90--1R97 1K1385

. . . . . . . . . . . . . . . . . . . . . . . . . . . . . . . . . . . . . . . .

psd analysis for psd peak list "2" (selected parent mass = 1542.509)

concise lists of psd assignments

simple chain fragmentation

chain 1 1 1

----- - - -

0 0 1

0 0 3

8 9 8

7 7 5

rule A V K T L L A L G E R K score

a 0.0

b 0.0

b' 0.0

b" 0.0

b-NH3 0.0

b-H2O 0.0

b(n-1)+H2O 0.0

y 0.0

y' 0.0

y" 0.0

y-NH3 9 2.0

y-H2O 9 2.0

linker fragmentation

link rule link score matching psd rule(s)

-------------------------------------------------------------------------------

egs 1K89-1K1385 6.0 legs12,legs14,legs15,legs17

detailed list of psd assignments

number of unassigned psd peaks 40

number of psd peaks assigned 6

number of psd fragments assigned 6

number of psd assignments 9

total score 10.0

m(exp) m(calc) d[Da] score psd rule fragmentation site(s)

-------------------------------------------------------------------------------

1298.667 1297.760 0.9 1.5 legs12 [1K89]-1K1385

1339.500 1340.742 1.2 1.5 legs14 [1K89]-1K1385

1341.000 1340.742 0.3 1.5 legs14 [1K89]-1K1385

1355.533 1354.758 0.8 2.0 y-H2O9 1V88-[1K89]

1355.533 1355.742 0.2 2.0 y-NH39 1V88-[1K89]

1355.533 1355.766 0.2 1.5 legs15 [1K89]-1K1385

1357.267 1355.742 1.5 2.0 y-NH39 1V88-[1K89]

1357.267 1355.766 1.5 1.5 legs15 [1K89]-1K1385

1397.467 1396.769 0.7 1.5 legs17 [1K89]-1K1385

*** legend ********************************************************************

peptide 19

number of base peptides = 3

number of missed cleavages = 1

number of optional links = 1

number of known links = 0

number of optional modifications = 0

number of known modifications = 0

calculated mass = 1542.8741

...............................................................................

isomer 1 / peptide 19

oooooooooooooooooooooo

1A87--1K89 ~ 1T90--1R97 2K1229

. . . . . . . . . . . . . . . . . . . . . . . . . . . . . . . . . . . . . . . .

psd analysis for psd peak list "2" (selected parent mass = 1542.509)

concise lists of psd assignments

simple chain fragmentation

chain 1 1 2

----- - - -

0 0 1

0 0 2

8 9 2

7 7 9

rule A V K T L L A L G E R K score

a 0.0

b 0.0

b' 0.0

b" 0.0

b-NH3 0.0

b-H2O 0.0

b(n-1)+H2O 0.0

y 0.0

y' 0.0

y" 0.0

y-NH3 9 2.0

y-H2O 9 2.0

linker fragmentation

link rule link score matching psd rule(s)

-------------------------------------------------------------------------------

egs 1K89-2K1229 6.0 legs12,legs14,legs15,legs17

detailed list of psd assignments

number of unassigned psd peaks 40

number of psd peaks assigned 6

number of psd fragments assigned 6

number of psd assignments 9

total score 10.0

m(exp) m(calc) d[Da] score psd rule fragmentation site(s)

-------------------------------------------------------------------------------

1298.667 1297.760 0.9 1.5 legs12 [1K89]-2K1229

1339.500 1340.742 1.2 1.5 legs14 [1K89]-2K1229

1341.000 1340.742 0.3 1.5 legs14 [1K89]-2K1229

1355.533 1354.758 0.8 2.0 y-H2O9 1V88-[1K89]

1355.533 1355.742 0.2 2.0 y-NH39 1V88-[1K89]

1355.533 1355.766 0.2 1.5 legs15 [1K89]-2K1229

1357.267 1355.742 1.5 2.0 y-NH39 1V88-[1K89]

1357.267 1355.766 1.5 1.5 legs15 [1K89]-2K1229

1397.467 1396.769 0.7 1.5 legs17 [1K89]-2K1229

*** legend ********************************************************************

peptide 20

number of base peptides = 3

number of missed cleavages = 1

number of optional links = 1

number of known links = 0

number of optional modifications = 0

number of known modifications = 0

calculated mass = 1542.8741

...............................................................................

isomer 1 / peptide 20

ooooooooooooooooooooo

1A87--1K89 ~ 1T90--1R97 2K90

. . . . . . . . . . . . . . . . . . . . . . . . . . . . . . . . . . . . . . . .

psd analysis for psd peak list "2" (selected parent mass = 1542.509)

concise lists of psd assignments

simple chain fragmentation

chain 1 1 2

----- - - -

8 9 9

7 7 0

rule A V K T L L A L G E R K score

a 0.0

b 0.0

b' 0.0

b" 0.0

b-NH3 0.0

b-H2O 0.0

b(n-1)+H2O 0.0

y 0.0

y' 0.0

y" 0.0

y-NH3 9 2.0

y-H2O 9 2.0

linker fragmentation

link rule link score matching psd rule(s)

-------------------------------------------------------------------------------

egs 1K89-2K90 6.0 legs12,legs14,legs15,legs17

detailed list of psd assignments

number of unassigned psd peaks 40

number of psd peaks assigned 6

number of psd fragments assigned 6

number of psd assignments 9

total score 10.0

m(exp) m(calc) d[Da] score psd rule fragmentation site(s)

-------------------------------------------------------------------------------

1298.667 1297.760 0.9 1.5 legs12 [1K89]-2K90

1339.500 1340.742 1.2 1.5 legs14 [1K89]-2K90

1341.000 1340.742 0.3 1.5 legs14 [1K89]-2K90

1355.533 1354.758 0.8 2.0 y-H2O9 1V88-[1K89]

1355.533 1355.742 0.2 2.0 y-NH39 1V88-[1K89]

1355.533 1355.766 0.2 1.5 legs15 [1K89]-2K90

1357.267 1355.742 1.5 2.0 y-NH39 1V88-[1K89]

1357.267 1355.766 1.5 1.5 legs15 [1K89]-2K90

1397.467 1396.769 0.7 1.5 legs17 [1K89]-2K90

*** legend ********************************************************************

peptide 21

number of base peptides = 3

number of missed cleavages = 1

number of optional links = 1

number of known links = 0

number of optional modifications = 0

number of known modifications = 0

calculated mass = 1542.8741

...............................................................................

isomer 1 / peptide 21

ooooooooooooooooooooo

1A87--1K89 ~ 1T90--1R97 1K717

. . . . . . . . . . . . . . . . . . . . . . . . . . . . . . . . . . . . . . . .

psd analysis for psd peak list "2" (selected parent mass = 1542.509)

concise lists of psd assignments

simple chain fragmentation

chain 1 1 1

----- - - -

0 0 7

8 9 1

7 7 7

rule A V K T L L A L G E R K score

a 0.0

b 0.0

b' 0.0

b" 0.0

b-NH3 0.0

b-H2O 0.0

b(n-1)+H2O 0.0

y 0.0

y' 0.0

y" 0.0

y-NH3 9 2.0

y-H2O 9 2.0

linker fragmentation

link rule link score matching psd rule(s)

-------------------------------------------------------------------------------

egs 1K89-1K717 6.0 legs12,legs14,legs15,legs17

detailed list of psd assignments

number of unassigned psd peaks 40

number of psd peaks assigned 6

number of psd fragments assigned 6

number of psd assignments 9

total score 10.0

m(exp) m(calc) d[Da] score psd rule fragmentation site(s)

-------------------------------------------------------------------------------

1298.667 1297.760 0.9 1.5 legs12 [1K89]-1K717

1339.500 1340.742 1.2 1.5 legs14 [1K89]-1K717

1341.000 1340.742 0.3 1.5 legs14 [1K89]-1K717

1355.533 1354.758 0.8 2.0 y-H2O9 1V88-[1K89]

1355.533 1355.742 0.2 2.0 y-NH39 1V88-[1K89]

1355.533 1355.766 0.2 1.5 legs15 [1K89]-1K717

1357.267 1355.742 1.5 2.0 y-NH39 1V88-[1K89]

1357.267 1355.766 1.5 1.5 legs15 [1K89]-1K717

1397.467 1396.769 0.7 1.5 legs17 [1K89]-1K717

*** legend ********************************************************************

peptide 22

number of base peptides = 3

number of missed cleavages = 1

number of optional links = 1

number of known links = 0

number of optional modifications = 0

number of known modifications = 0

calculated mass = 1542.8741

...............................................................................

isomer 1 / peptide 22

oooooooooooooooooooooo

1A87--1K89 ~ 1T90--1R97 2K1120

. . . . . . . . . . . . . . . . . . . . . . . . . . . . . . . . . . . . . . . .

psd analysis for psd peak list "2" (selected parent mass = 1542.509)

concise lists of psd assignments

simple chain fragmentation

chain 1 1 2

----- - - -

0 0 1

0 0 1

8 9 2

7 7 0

rule A V K T L L A L G E R K score

a 0.0

b 0.0

b' 0.0

b" 0.0

b-NH3 0.0

b-H2O 0.0

b(n-1)+H2O 0.0

y 0.0

y' 0.0

y" 0.0

y-NH3 9 2.0

y-H2O 9 2.0

linker fragmentation

link rule link score matching psd rule(s)

-------------------------------------------------------------------------------

egs 1K89-2K1120 6.0 legs12,legs14,legs15,legs17

detailed list of psd assignments

number of unassigned psd peaks 40

number of psd peaks assigned 6

number of psd fragments assigned 6

number of psd assignments 9

total score 10.0

m(exp) m(calc) d[Da] score psd rule fragmentation site(s)

-------------------------------------------------------------------------------

1298.667 1297.760 0.9 1.5 legs12 [1K89]-2K1120

1339.500 1340.742 1.2 1.5 legs14 [1K89]-2K1120

1341.000 1340.742 0.3 1.5 legs14 [1K89]-2K1120

1355.533 1354.758 0.8 2.0 y-H2O9 1V88-[1K89]

1355.533 1355.742 0.2 2.0 y-NH39 1V88-[1K89]

1355.533 1355.766 0.2 1.5 legs15 [1K89]-2K1120

1357.267 1355.742 1.5 2.0 y-NH39 1V88-[1K89]

1357.267 1355.766 1.5 1.5 legs15 [1K89]-2K1120

1397.467 1396.769 0.7 1.5 legs17 [1K89]-2K1120

*** legend ********************************************************************

peptide 23

number of base peptides = 3

number of missed cleavages = 1

number of optional links = 1

number of known links = 0

number of optional modifications = 0

number of known modifications = 0

calculated mass = 1542.8741

...............................................................................

isomer 1 / peptide 23

ooooooooooooooooooooo

1A87--1K89 ~ 1T90--1R97 2K730

. . . . . . . . . . . . . . . . . . . . . . . . . . . . . . . . . . . . . . . .

psd analysis for psd peak list "2" (selected parent mass = 1542.509)

concise lists of psd assignments

simple chain fragmentation

chain 1 1 2

----- - - -

0 0 7

8 9 3

7 7 0

rule A V K T L L A L G E R K score

a 0.0

b 0.0

b' 0.0

b" 0.0

b-NH3 0.0

b-H2O 0.0

b(n-1)+H2O 0.0

y 0.0

y' 0.0

y" 0.0

y-NH3 9 2.0

y-H2O 9 2.0

linker fragmentation

link rule link score matching psd rule(s)

-------------------------------------------------------------------------------

egs 1K89-2K730 6.0 legs12,legs14,legs15,legs17

detailed list of psd assignments

number of unassigned psd peaks 40

number of psd peaks assigned 6

number of psd fragments assigned 6

number of psd assignments 9

total score 10.0

m(exp) m(calc) d[Da] score psd rule fragmentation site(s)

-------------------------------------------------------------------------------

1298.667 1297.760 0.9 1.5 legs12 [1K89]-2K730

1339.500 1340.742 1.2 1.5 legs14 [1K89]-2K730

1341.000 1340.742 0.3 1.5 legs14 [1K89]-2K730

1355.533 1354.758 0.8 2.0 y-H2O9 1V88-[1K89]

1355.533 1355.742 0.2 2.0 y-NH39 1V88-[1K89]

1355.533 1355.766 0.2 1.5 legs15 [1K89]-2K730

1357.267 1355.742 1.5 2.0 y-NH39 1V88-[1K89]

1357.267 1355.766 1.5 1.5 legs15 [1K89]-2K730

1397.467 1396.769 0.7 1.5 legs17 [1K89]-2K730

*** legend ********************************************************************

peptide 24

number of base peptides = 3

number of missed cleavages = 1

number of optional links = 1

number of known links = 0

number of optional modifications = 0

number of known modifications = 0

calculated mass = 1542.8741

...............................................................................

isomer 1 / peptide 24

oooooooooooooooooooooo

1A87--1K89 ~ 1T90--1R97 2K1350

. . . . . . . . . . . . . . . . . . . . . . . . . . . . . . . . . . . . . . . .

psd analysis for psd peak list "2" (selected parent mass = 1542.509)

concise lists of psd assignments

simple chain fragmentation

chain 1 1 2

----- - - -

0 0 1

0 0 3

8 9 5

7 7 0

rule A V K T L L A L G E R K score

a 0.0

b 0.0

b' 0.0

b" 0.0

b-NH3 0.0

b-H2O 0.0

b(n-1)+H2O 0.0

y 0.0

y' 0.0

y" 0.0

y-NH3 9 2.0

y-H2O 9 2.0

linker fragmentation

link rule link score matching psd rule(s)

-------------------------------------------------------------------------------

egs 1K89-2K1350 6.0 legs12,legs14,legs15,legs17

detailed list of psd assignments

number of unassigned psd peaks 40

number of psd peaks assigned 6

number of psd fragments assigned 6

number of psd assignments 9

total score 10.0

m(exp) m(calc) d[Da] score psd rule fragmentation site(s)

-------------------------------------------------------------------------------

1298.667 1297.760 0.9 1.5 legs12 [1K89]-2K1350

1339.500 1340.742 1.2 1.5 legs14 [1K89]-2K1350

1341.000 1340.742 0.3 1.5 legs14 [1K89]-2K1350

1355.533 1354.758 0.8 2.0 y-H2O9 1V88-[1K89]

1355.533 1355.742 0.2 2.0 y-NH39 1V88-[1K89]

1355.533 1355.766 0.2 1.5 legs15 [1K89]-2K1350

1357.267 1355.742 1.5 2.0 y-NH39 1V88-[1K89]

1357.267 1355.766 1.5 1.5 legs15 [1K89]-2K1350

1397.467 1396.769 0.7 1.5 legs17 [1K89]-2K1350

*** legend ********************************************************************

peptide 25

number of base peptides = 3

number of missed cleavages = 1

number of optional links = 1

number of known links = 0

number of optional modifications = 0

number of known modifications = 0

calculated mass = 1542.8741

...............................................................................

isomer 1 / peptide 25

ooooooooooooooooooooo

1A87--1K89 ~ 1T90--1R97 1K247

. . . . . . . . . . . . . . . . . . . . . . . . . . . . . . . . . . . . . . . .

psd analysis for psd peak list "2" (selected parent mass = 1542.509)

concise lists of psd assignments

simple chain fragmentation

chain 1 1 1

----- - - -

0 0 2

8 9 4

7 7 7

rule A V K T L L A L G E R K score

a 0.0

b 0.0

b' 0.0

b" 0.0

b-NH3 0.0

b-H2O 0.0

b(n-1)+H2O 0.0

y 0.0

y' 0.0

y" 0.0

y-NH3 9 2.0

y-H2O 9 2.0

linker fragmentation

link rule link score matching psd rule(s)

-------------------------------------------------------------------------------

egs 1K89-1K247 6.0 legs12,legs14,legs15,legs17

detailed list of psd assignments

number of unassigned psd peaks 40

number of psd peaks assigned 6

number of psd fragments assigned 6

number of psd assignments 9

total score 10.0

m(exp) m(calc) d[Da] score psd rule fragmentation site(s)

-------------------------------------------------------------------------------

1298.667 1297.760 0.9 1.5 legs12 [1K89]-1K247

1339.500 1340.742 1.2 1.5 legs14 [1K89]-1K247

1341.000 1340.742 0.3 1.5 legs14 [1K89]-1K247

1355.533 1354.758 0.8 2.0 y-H2O9 1V88-[1K89]

1355.533 1355.742 0.2 2.0 y-NH39 1V88-[1K89]

1355.533 1355.766 0.2 1.5 legs15 [1K89]-1K247

1357.267 1355.742 1.5 2.0 y-NH39 1V88-[1K89]

1357.267 1355.766 1.5 1.5 legs15 [1K89]-1K247

1397.467 1396.769 0.7 1.5 legs17 [1K89]-1K247

*** legend ********************************************************************

peptide 26

number of base peptides = 3

number of missed cleavages = 1

number of optional links = 1

number of known links = 0

number of optional modifications = 0

number of known modifications = 0

calculated mass = 1542.8741

...............................................................................

isomer 1 / peptide 26

ooooooooooooooooooooo

1A87--1K89 ~ 1T90--1R97 2K672

. . . . . . . . . . . . . . . . . . . . . . . . . . . . . . . . . . . . . . . .

psd analysis for psd peak list "2" (selected parent mass = 1542.509)

concise lists of psd assignments

simple chain fragmentation

chain 1 1 2

----- - - -

0 0 6

8 9 7

7 7 2

rule A V K T L L A L G E R K score

a 0.0

b 0.0

b' 0.0

b" 0.0

b-NH3 0.0

b-H2O 0.0

b(n-1)+H2O 0.0

y 0.0

y' 0.0

y" 0.0

y-NH3 9 2.0

y-H2O 9 2.0

linker fragmentation

link rule link score matching psd rule(s)

-------------------------------------------------------------------------------

egs 1K89-2K672 6.0 legs12,legs14,legs15,legs17

detailed list of psd assignments

number of unassigned psd peaks 40

number of psd peaks assigned 6

number of psd fragments assigned 6

number of psd assignments 9

total score 10.0

m(exp) m(calc) d[Da] score psd rule fragmentation site(s)

-------------------------------------------------------------------------------

1298.667 1297.760 0.9 1.5 legs12 [1K89]-2K672

1339.500 1340.742 1.2 1.5 legs14 [1K89]-2K672

1341.000 1340.742 0.3 1.5 legs14 [1K89]-2K672

1355.533 1354.758 0.8 2.0 y-H2O9 1V88-[1K89]

1355.533 1355.742 0.2 2.0 y-NH39 1V88-[1K89]

1355.533 1355.766 0.2 1.5 legs15 [1K89]-2K672

1357.267 1355.742 1.5 2.0 y-NH39 1V88-[1K89]

1357.267 1355.766 1.5 1.5 legs15 [1K89]-2K672

1397.467 1396.769 0.7 1.5 legs17 [1K89]-2K672

*** legend ********************************************************************

peptide 27

number of base peptides = 2

number of missed cleavages = 0

number of optional links = 1

number of known links = 0

number of optional modifications = 0

number of known modifications = 0

calculated mass = 1405.7400

...............................................................................

isomer 1 / peptide 27

ooooooooooooooooo

1A87--1K89 1V1036--1K1038--1K1042

. . . . . . . . . . . . . . . . . . . . . . . . . . . . . . . . . . . . . . . .

psd analysis for psd peak list "8" (selected parent mass = 1405.492)

concise lists of psd assignments

simple chain fragmentation

chain 1 1 1 1

----- - - - -

0 0 1 1

0 0 0 0

8 8 3 4

7 9 6 2

rule A V K V N K P M F K score

a 0.0

b 6 2.0

b' 0.0

b" 0.0

b-NH3 0.0

b-H2O 0.0

b(n-1)+H2O 0.0

y 0.0

y' 0.0

y" 0.0

y-NH3 0.0

y-H2O 0.0

linker fragmentation

link rule link score matching psd rule(s)

-------------------------------------------------------------------------------

egs 1K89-1K1038 1.5 regs17

detailed list of psd assignments

number of unassigned psd peaks 17

number of psd peaks assigned 2

number of psd fragments assigned 2

number of psd assignments 2

total score 3.5

m(exp) m(calc) d[Da] score psd rule fragmentation site(s)

-------------------------------------------------------------------------------

1088.533 1089.529 1.0 1.5 regs17 1K89-[1K1038]

1260.467 1259.634 0.8 2.0 b6 [1F1041]-1K1042

...............................................................................

isomer 2 / peptide 27

ooooooooooooooooo

1A87--1K89 1V1036--1K1042

. . . . . . . . . . . . . . . . . . . . . . . . . . . . . . . . . . . . . . . .

psd analysis for psd peak list "8" (selected parent mass = 1405.492)

concise lists of psd assignments

simple chain fragmentation

no assignments of simple chain fragments

linker fragmentation

link rule link score matching psd rule(s)

-------------------------------------------------------------------------------

egs 1K89-1K1042 1.5 regs17

detailed list of psd assignments

number of unassigned psd peaks 18

number of psd peaks assigned 1

number of psd fragments assigned 1

number of psd assignments 1

total score 1.5

m(exp) m(calc) d[Da] score psd rule fragmentation site(s)

-------------------------------------------------------------------------------

1088.533 1089.529 1.0 1.5 regs17 1K89-[1K1042]

*** legend ********************************************************************

peptide 28

number of base peptides = 2

number of missed cleavages = 0

number of optional links = 1

number of known links = 0

number of optional modifications = 0

number of known modifications = 0

calculated mass = 1541.8061

...............................................................................

isomer 1 / peptide 28

ooooooooooooooooo

2E476--2K479 2H999--2K1006

. . . . . . . . . . . . . . . . . . . . . . . . . . . . . . . . . . . . . . . .

psd analysis for psd peak list "2" (selected parent mass = 1542.509)

concise lists of psd assignments

simple chain fragmentation

chain 2 2 2 2

----- - - - -

0 0 0 1

4 4 9 0

7 7 9 0

6 9 9 6

rule E A I K H A I S V T T K score

a 0.0

b 0.0

b' 0.0

b" 0.0

b-NH3 0.0

b-H2O 0.0

b(n-1)+H2O 0.0

y 3 2 1 7 6 2 22.0

y' 3 1.5

y" 0.0

y-NH3 0.0

y-H2O 2 7 6 5 4 3 2 128.0

linker fragmentation

link rule link score matching psd rule(s)

-------------------------------------------------------------------------------

egs 2K479-2K1006 1.5 regs6

detailed list of psd assignments

number of unassigned psd peaks 32

number of psd peaks assigned 14

number of psd fragments assigned 15

number of psd assignments 15

total score 153.0

m(exp) m(calc) d[Da] score psd rule fragmentation site(s)

-------------------------------------------------------------------------------

881.400 882.468 1.1 1.5 regs6 2K479-[2K1006]

915.000 915.467 0.5 64.0 y-H2O2 2T1004-[2T1005]

931.500 933.478 2.0 2.0 y2 2T1004-[2T1005]

1016.267 1016.515 0.2 32.0 y-H2O3 2V1003-[2T1004]

1113.867 1115.583 1.7 16.0 y-H2O4 2S1002-[2V1003]

1204.000 1202.615 1.4 8.0 y-H2O5 2I1001-[2S1002]

1230.000 1228.642 1.4 8.0 y1 2I478-[2K479]

1315.533 1315.700 0.2 4.0 y-H2O6 2A1000-[2I1001]

1323.500 1323.716 0.2 2.0 y-H2O2 2A477-[2I478]

1334.667 1333.710 1.0 4.0 y6 2A1000-[2I1001]

1341.000 1341.726 0.7 4.0 y2 2A477-[2I478]

1385.200 1386.737 1.5 2.0 y-H2O7 2H999-[2A1000]

1403.500 1404.747 1.2 2.0 y7 2H999-[2A1000]

1411.400 1412.764 1.4 2.0 y3 2E476-[2A477]

1411.400 1412.764 1.4 1.5 y'3 2E476-[2A477]

*** legend ********************************************************************

peptide 29

number of base peptides = 2

number of missed cleavages = 0

number of optional links = 1

number of known links = 0

number of optional modifications = 0

number of known modifications = 0

calculated mass = 1541.8061

...............................................................................

isomer 1 / peptide 29

oooooooooooooooo

1H999--1K1006 2E476--2K479

. . . . . . . . . . . . . . . . . . . . . . . . . . . . . . . . . . . . . . . .

psd analysis for psd peak list "2" (selected parent mass = 1542.509)

concise lists of psd assignments

simple chain fragmentation

chain 1 1 2 2

----- - - - -

0 1 0 0

9 0 4 4

9 0 7 7

9 6 6 9

rule H A I S V T T K E A I K score

a 0.0

b 0.0

b' 0.0

b" 0.0

b-NH3 0.0

b-H2O 0.0

b(n-1)+H2O 0.0

y 7 6 2 3 2 1 22.0

y' 3 1.5

y" 0.0

y-NH3 0.0

y-H2O 7 6 5 4 3 2 2 128.0

linker fragmentation

link rule link score matching psd rule(s)

-------------------------------------------------------------------------------

egs 1K1006-2K479 1.5 legs6

detailed list of psd assignments

number of unassigned psd peaks 32

number of psd peaks assigned 14

number of psd fragments assigned 15

number of psd assignments 15

total score 153.0

m(exp) m(calc) d[Da] score psd rule fragmentation site(s)

-------------------------------------------------------------------------------

881.400 882.468 1.1 1.5 legs6 [1K1006]-2K479

915.000 915.467 0.5 64.0 y-H2O2 1T1004-[1T1005]

931.500 933.478 2.0 2.0 y2 1T1004-[1T1005]

1016.267 1016.515 0.2 32.0 y-H2O3 1V1003-[1T1004]

1113.867 1115.583 1.7 16.0 y-H2O4 1S1002-[1V1003]

1204.000 1202.615 1.4 8.0 y-H2O5 1I1001-[1S1002]

1230.000 1228.642 1.4 8.0 y1 2I478-[2K479]

1315.533 1315.700 0.2 4.0 y-H2O6 1A1000-[1I1001]

1323.500 1323.716 0.2 2.0 y-H2O2 2A477-[2I478]

1334.667 1333.710 1.0 4.0 y6 1A1000-[1I1001]

1341.000 1341.726 0.7 4.0 y2 2A477-[2I478]

1385.200 1386.737 1.5 2.0 y-H2O7 1H999-[1A1000]

1403.500 1404.747 1.2 2.0 y7 1H999-[1A1000]

1411.400 1412.764 1.4 2.0 y3 2E476-[2A477]

1411.400 1412.764 1.4 1.5 y'3 2E476-[2A477]

*** legend ********************************************************************

peptide 30

number of base peptides = 3

number of missed cleavages = 1

number of optional links = 1

number of known links = 0

number of optional modifications = 0

number of known modifications = 0

calculated mass = 1542.8126

...............................................................................

isomer 1 / peptide 30

ooooooooooooooooooooooooooooooo

1K717 ~ 1L718--1R720 2G330--2K337

. . . . . . . . . . . . . . . . . . . . . . . . . . . . . . . . . . . . . . . .

psd analysis for psd peak list "2" (selected parent mass = 1542.509)

concise lists of psd assignments

simple chain fragmentation

chain 1 1 2 2

----- - - - -

7 7 3 3

1 2 3 3

7 0 0 7

rule K L G R G N L Q E V G K score

a 0.0

b 0.0

b' 0.0

b" 0.0

b-NH3 0.0

b-H2O 0.0

b(n-1)+H2O 0.0

y 0.0

y' 0.0

y" 0.0

y-NH3 7 6 4 8.0

y-H2O 0.0

linker fragmentation

link rule link score matching psd rule(s)

-------------------------------------------------------------------------------

egs 1K717-2K337 3.0 regs14,regs16

detailed list of psd assignments

number of unassigned psd peaks 40

number of psd peaks assigned 6

number of psd fragments assigned 5

number of psd assignments 6

total score 11.0

m(exp) m(calc) d[Da] score psd rule fragmentation site(s)

-------------------------------------------------------------------------------

1016.267 1014.474 1.8 1.5 regs14 1K717-[2K337]

1044.500 1043.513 1.0 1.5 regs16 1K717-[2K337]

1113.867 1113.579 0.3 2.0 y-NH34 2Q333-[2E334]

1355.533 1354.722 0.8 4.0 y-NH36 2N331-[2L332]

1468.600 1468.765 0.2 2.0 y-NH37 2G330-[2N331]

1469.500 1468.765 0.7 2.0 y-NH37 2G330-[2N331]

*** legend ********************************************************************

peptide 31

number of base peptides = 3

number of missed cleavages = 1

number of optional links = 1

number of known links = 0

number of optional modifications = 0

number of known modifications = 0

calculated mass = 1542.8126

...............................................................................

isomer 1 / peptide 31

ooooooooo

2G330--2K337 2K717 ~ 2L718--2R720

. . . . . . . . . . . . . . . . . . . . . . . . . . . . . . . . . . . . . . . .

psd analysis for psd peak list "2" (selected parent mass = 1542.509)

concise lists of psd assignments

simple chain fragmentation

chain 2 2 2 2

----- - - - -

3 3 7 7

3 3 1 2

0 7 7 0

rule G N L Q E V G K K L G R score

a 0.0

b 0.0

b' 0.0

b" 0.0

b-NH3 0.0

b-H2O 0.0

b(n-1)+H2O 0.0

y 0.0

y' 0.0

y" 0.0

y-NH3 7 6 4 8.0

y-H2O 0.0

linker fragmentation

link rule link score matching psd rule(s)

-------------------------------------------------------------------------------

egs 2K337-2K717 3.0 legs14,legs16

detailed list of psd assignments

number of unassigned psd peaks 40

number of psd peaks assigned 6

number of psd fragments assigned 5

number of psd assignments 6

total score 11.0

m(exp) m(calc) d[Da] score psd rule fragmentation site(s)

-------------------------------------------------------------------------------

1016.267 1014.474 1.8 1.5 legs14 [2K337]-2K717

1044.500 1043.513 1.0 1.5 legs16 [2K337]-2K717

1113.867 1113.579 0.3 2.0 y-NH34 2Q333-[2E334]

1355.533 1354.722 0.8 4.0 y-NH36 2N331-[2L332]

1468.600 1468.765 0.2 2.0 y-NH37 2G330-[2N331]

1469.500 1468.765 0.7 2.0 y-NH37 2G330-[2N331]

*** legend ********************************************************************

peptide 32

number of base peptides = 3

number of missed cleavages = 1

number of optional links = 1

number of known links = 0

number of optional modifications = 0

number of known modifications = 0

calculated mass = 1542.8126

...............................................................................

isomer 1 / peptide 32

ooooooooo

1G330--1K337 2K717 ~ 2L718--2R720

. . . . . . . . . . . . . . . . . . . . . . . . . . . . . . . . . . . . . . . .

psd analysis for psd peak list "2" (selected parent mass = 1542.509)

concise lists of psd assignments

simple chain fragmentation

chain 1 1 2 2

----- - - - -

3 3 7 7

3 3 1 2

0 7 7 0

rule G N L Q E V G K K L G R score

a 0.0

b 0.0

b' 0.0

b" 0.0

b-NH3 0.0

b-H2O 0.0

b(n-1)+H2O 0.0

y 0.0

y' 0.0

y" 0.0

y-NH3 7 6 4 8.0

y-H2O 0.0

linker fragmentation

link rule link score matching psd rule(s)

-------------------------------------------------------------------------------

egs 1K337-2K717 3.0 legs14,legs16

detailed list of psd assignments

number of unassigned psd peaks 40

number of psd peaks assigned 6

number of psd fragments assigned 5

number of psd assignments 6

total score 11.0

m(exp) m(calc) d[Da] score psd rule fragmentation site(s)

-------------------------------------------------------------------------------

1016.267 1014.474 1.8 1.5 legs14 [1K337]-2K717

1044.500 1043.513 1.0 1.5 legs16 [1K337]-2K717

1113.867 1113.579 0.3 2.0 y-NH34 1Q333-[1E334]

1355.533 1354.722 0.8 4.0 y-NH36 1N331-[1L332]

1468.600 1468.765 0.2 2.0 y-NH37 1G330-[1N331]

1469.500 1468.765 0.7 2.0 y-NH37 1G330-[1N331]

*** legend ********************************************************************

peptide 33

number of base peptides = 3

number of missed cleavages = 1

number of optional links = 1

number of known links = 0

number of optional modifications = 0

number of known modifications = 0

calculated mass = 1542.8126

...............................................................................

isomer 1 / peptide 33

ooooooooo

1G330--1K337 1K717 ~ 1L718--1R720

. . . . . . . . . . . . . . . . . . . . . . . . . . . . . . . . . . . . . . . .

psd analysis for psd peak list "2" (selected parent mass = 1542.509)

concise lists of psd assignments

simple chain fragmentation

chain 1 1 1 1

----- - - - -

3 3 7 7

3 3 1 2

0 7 7 0

rule G N L Q E V G K K L G R score

a 0.0

b 0.0

b' 0.0

b" 0.0

b-NH3 0.0

b-H2O 0.0

b(n-1)+H2O 0.0

y 0.0

y' 0.0

y" 0.0

y-NH3 7 6 4 8.0

y-H2O 0.0

linker fragmentation

link rule link score matching psd rule(s)

-------------------------------------------------------------------------------

egs 1K337-1K717 3.0 legs14,legs16

detailed list of psd assignments

number of unassigned psd peaks 40

number of psd peaks assigned 6

number of psd fragments assigned 5

number of psd assignments 6

total score 11.0

m(exp) m(calc) d[Da] score psd rule fragmentation site(s)

-------------------------------------------------------------------------------

1016.267 1014.474 1.8 1.5 legs14 [1K337]-1K717

1044.500 1043.513 1.0 1.5 legs16 [1K337]-1K717

1113.867 1113.579 0.3 2.0 y-NH34 1Q333-[1E334]

1355.533 1354.722 0.8 4.0 y-NH36 1N331-[1L332]

1468.600 1468.765 0.2 2.0 y-NH37 1G330-[1N331]

1469.500 1468.765 0.7 2.0 y-NH37 1G330-[1N331]

*** legend ********************************************************************

peptide 34

number of base peptides = 3

number of missed cleavages = 1

number of optional links = 1

number of known links = 0

number of optional modifications = 0

number of known modifications = 0

calculated mass = 1541.7598

...............................................................................

isomer 1 / peptide 34

ooooooooooooooooooooooooooooooooooo

1K1350 ~ 1V1351--1R1353 2F1060--2K1066

. . . . . . . . . . . . . . . . . . . . . . . . . . . . . . . . . . . . . . . .

psd analysis for psd peak list "2" (selected parent mass = 1542.509)

concise lists of psd assignments

simple chain fragmentation

no assignments of simple chain fragments

linker fragmentation

link rule link score matching psd rule(s)

-------------------------------------------------------------------------------

egs 1K1350-2K1066 4.5 legs8,regs9,regs11

detailed list of psd assignments

number of unassigned psd peaks 43

number of psd peaks assigned 3

number of psd fragments assigned 3

number of psd assignments 3

total score 4.5

m(exp) m(calc) d[Da] score psd rule fragmentation site(s)

-------------------------------------------------------------------------------

570.533 571.344 0.8 1.5 legs8 [1K1350]-2K1066

881.400 882.400 1.0 1.5 regs9 1K1350-[2K1066]

915.000 913.418 1.6 1.5 regs11 1K1350-[2K1066]

*** legend ********************************************************************

peptide 35

number of base peptides = 2

number of missed cleavages = 0

number of optional links = 1

number of known links = 0

number of optional modifications = 0

number of known modifications = 0

calculated mass = 1541.8101

...............................................................................

isomer 1 / peptide 35

oooooooooooooooo

2Y296--2K302 2A338--2K342

. . . . . . . . . . . . . . . . . . . . . . . . . . . . . . . . . . . . . . . .

psd analysis for psd peak list "2" (selected parent mass = 1542.509)

concise lists of psd assignments

simple chain fragmentation

chain 2 2 2 2

----- - - - -

2 3 3 3

9 0 3 4

6 2 8 2

rule Y I S I G L K A T F G K score

a 0.0

b 5 2.0

b' 0.0

b" 0.0

b-NH3 0.0

b-H2O 0.0

b(n-1)+H2O 0.0

y 4 1 4.0

y' 0.0

y" 0.0

y-NH3 0.0

y-H2O 4 2 4.0

linker fragmentation

link rule link score matching psd rule(s)

-------------------------------------------------------------------------------

egs 2K302-2K342 1.5 legs5

detailed list of psd assignments

number of unassigned psd peaks 40

number of psd peaks assigned 6

number of psd fragments assigned 6

number of psd assignments 6

total score 11.5

m(exp) m(calc) d[Da] score psd rule fragmentation site(s)

-------------------------------------------------------------------------------

536.200 534.293 1.9 2.0 b5 [2G300]-2L301

793.133 793.482 0.3 1.5 legs5 [2K302]-2K342

1167.000 1165.636 1.4 2.0 y1 2G341-[2K342]

1204.000 1204.646 0.6 2.0 y-H2O2 2F340-[2G341]

1451.533 1452.762 1.2 2.0 y-H2O4 2A338-[2T339]

1469.500 1470.773 1.3 2.0 y4 2A338-[2T339]

*** legend ********************************************************************

peptide 36

number of base peptides = 2

number of missed cleavages = 0

number of optional links = 1

number of known links = 0

number of optional modifications = 0

number of known modifications = 0

calculated mass = 1541.8101

...............................................................................

isomer 1 / peptide 36

oooooooooooooooo

1A338--1K342 2Y296--2K302

. . . . . . . . . . . . . . . . . . . . . . . . . . . . . . . . . . . . . . . .

psd analysis for psd peak list "2" (selected parent mass = 1542.509)

concise lists of psd assignments

simple chain fragmentation

chain 1 1 2 2

----- - - - -

3 3 2 3

3 4 9 0

8 2 6 2

rule A T F G K Y I S I G L K score

a 0.0

b 5 2.0

b' 0.0

b" 0.0

b-NH3 0.0

b-H2O 0.0

b(n-1)+H2O 0.0

y 4 1 4.0

y' 0.0

y" 0.0

y-NH3 0.0

y-H2O 4 2 4.0

linker fragmentation

link rule link score matching psd rule(s)

-------------------------------------------------------------------------------

egs 1K342-2K302 1.5 regs5

detailed list of psd assignments

number of unassigned psd peaks 40

number of psd peaks assigned 6

number of psd fragments assigned 6

number of psd assignments 6

total score 11.5

m(exp) m(calc) d[Da] score psd rule fragmentation site(s)

-------------------------------------------------------------------------------

536.200 534.293 1.9 2.0 b5 [2G300]-2L301

793.133 793.482 0.3 1.5 regs5 1K342-[2K302]

1167.000 1165.636 1.4 2.0 y1 1G341-[1K342]

1204.000 1204.646 0.6 2.0 y-H2O2 1F340-[1G341]

1451.533 1452.762 1.2 2.0 y-H2O4 1A338-[1T339]

1469.500 1470.773 1.3 2.0 y4 1A338-[1T339]

*** legend ********************************************************************

peptide 37

number of base peptides = 2

number of missed cleavages = 0

number of optional links = 1

number of known links = 0

number of optional modifications = 0

number of known modifications = 0

calculated mass = 1541.7598

...............................................................................

isomer 1 / peptide 37

oooooooooooooooooo

1S1067--1K1070 2S1338--2K1344

. . . . . . . . . . . . . . . . . . . . . . . . . . . . . . . . . . . . . . . .

psd analysis for psd peak list "2" (selected parent mass = 1542.509)

concise lists of psd assignments

simple chain fragmentation

chain 1 1 2 2

----- - - - -

1 1 1 1

0 0 3 3

6 7 3 4

7 0 8 4

rule S W H K S L N Q L G K score

a 0.0

b 0.0

b' 0.0

b" 0.0

b-NH3 0.0

b-H2O 0.0

b(n-1)+H2O 0.0

y 5 2.0

y' 0.0

y" 0.0

y-NH3 0.0

y-H2O 1 5 4.0

linker fragmentation

no assignments of linker fragments

detailed list of psd assignments

number of unassigned psd peaks 43

number of psd peaks assigned 3

number of psd fragments assigned 3

number of psd assignments 3

total score 6.0

m(exp) m(calc) d[Da] score psd rule fragmentation site(s)

-------------------------------------------------------------------------------

1113.867 1113.579 0.3 2.0 y-H2O1 1H1069-[1K1070]

1323.500 1323.633 0.1 2.0 y-H2O5 2L1339-[2N1340]

1341.000 1341.644 0.6 2.0 y5 2L1339-[2N1340]

*** legend ********************************************************************

peptide 38

number of base peptides = 3

number of missed cleavages = 1

number of optional links = 1

number of known links = 0

number of optional modifications = 0

number of known modifications = 0

calculated mass = 1542.8741

...............................................................................

isomer 1 / peptide 38

oooooooo

1A12--1K15 2K90 ~ 2L91--2R97

. . . . . . . . . . . . . . . . . . . . . . . . . . . . . . . . . . . . . . . .

psd analysis for psd peak list "2" (selected parent mass = 1542.509)

concise lists of psd assignments

simple chain fragmentation

chain 1 1 2 2

----- - - - -

1 1 9 9

2 5 0 7

rule A T I K K L V A L G E R score

a 0.0

b 0.0

b' 0.0

b" 0.0

b-NH3 0.0

b-H2O 2 2.0

b(n-1)+H2O 0.0

y 0.0

y' 0.0

y" 0.0

y-NH3 2 2.0

y-H2O 0.0

linker fragmentation

no assignments of linker fragments

detailed list of psd assignments

number of unassigned psd peaks 44

number of psd peaks assigned 2

number of psd fragments assigned 2

number of psd assignments 2

total score 4.0

m(exp) m(calc) d[Da] score psd rule fragmentation site(s)

-------------------------------------------------------------------------------

881.400 881.498 0.1 2.0 b-H2O2 [2L91]-2V92

1355.533 1353.763 1.8 2.0 y-NH32 1T13-[1I14]

*** legend ********************************************************************

peptide 39

number of base peptides = 3

number of missed cleavages = 1

number of optional links = 1

number of known links = 0

number of optional modifications = 0

number of known modifications = 0

calculated mass = 1542.8741

...............................................................................

isomer 1 / peptide 39

oooooooo

2A12--2K15 2K90 ~ 2L91--2R97

. . . . . . . . . . . . . . . . . . . . . . . . . . . . . . . . . . . . . . . .

psd analysis for psd peak list "2" (selected parent mass = 1542.509)

concise lists of psd assignments

simple chain fragmentation

chain 2 2 2 2

----- - - - -

1 1 9 9

2 5 0 7

rule A T I K K L V A L G E R score

a 0.0

b 0.0

b' 0.0

b" 0.0

b-NH3 0.0

b-H2O 2 2.0

b(n-1)+H2O 0.0

y 0.0

y' 0.0

y" 0.0

y-NH3 2 2.0

y-H2O 0.0

linker fragmentation

no assignments of linker fragments

detailed list of psd assignments

number of unassigned psd peaks 44

number of psd peaks assigned 2

number of psd fragments assigned 2

number of psd assignments 2

total score 4.0

m(exp) m(calc) d[Da] score psd rule fragmentation site(s)

-------------------------------------------------------------------------------

881.400 881.498 0.1 2.0 b-H2O2 [2L91]-2V92

1355.533 1353.763 1.8 2.0 y-NH32 2T13-[2I14]

*** legend ********************************************************************

peptide 40

number of base peptides = 2

number of missed cleavages = 0

number of optional links = 1

number of known links = 0

number of optional modifications = 0

number of known modifications = 0

calculated mass = 1405.7213

...............................................................................

isomer 1 / peptide 40

oooooooooooooooo

2N648--2K653 2N668--2K671

. . . . . . . . . . . . . . . . . . . . . . . . . . . . . . . . . . . . . . . .

psd analysis for psd peak list "8" (selected parent mass = 1405.492)

concise lists of psd assignments

simple chain fragmentation

chain 2 2 2 2

----- - - - -

6 6 6 6

4 5 6 7

8 3 8 1

rule N V E I G K N L F K score

a 0.0

b 0.0

b' 0.0

b" 0.0

b-NH3 0.0

b-H2O 0.0

b(n-1)+H2O 0.0

y 5 1 3 6.0

y' 0.0

y" 0.0

y-NH3 0.0

y-H2O 0.0

linker fragmentation

link rule link score matching psd rule(s)

-------------------------------------------------------------------------------

egs 2K653-2K671 1.5 regs11

detailed list of psd assignments

number of unassigned psd peaks 16

number of psd peaks assigned 3

number of psd fragments assigned 4

number of psd assignments 4

total score 7.5

m(exp) m(calc) d[Da] score psd rule fragmentation site(s)

-------------------------------------------------------------------------------

634.200 634.333 0.1 1.5 regs11 2K653-[2K671]

893.267 893.462 0.2 2.0 y1 2G652-[2K653]

1290.533 1291.678 1.1 2.0 y5 2N648-[2V649]

1290.533 1291.678 1.1 2.0 y3 2N668-[2L669]

*** legend ********************************************************************

peptide 41

number of base peptides = 2

number of missed cleavages = 0

number of optional links = 1

number of known links = 0

number of optional modifications = 0

number of known modifications = 0

calculated mass = 1405.7213

...............................................................................

isomer 1 / peptide 41

oooooooooooooooo

1N668--1K671 2N648--2K653

. . . . . . . . . . . . . . . . . . . . . . . . . . . . . . . . . . . . . . . .

psd analysis for psd peak list "8" (selected parent mass = 1405.492)

concise lists of psd assignments

simple chain fragmentation

chain 1 1 2 2

----- - - - -

6 6 6 6

6 7 4 5

8 1 8 3

rule N L F K N V E I G K score

a 0.0

b 0.0

b' 0.0

b" 0.0

b-NH3 0.0

b-H2O 0.0

b(n-1)+H2O 0.0

y 3 5 1 6.0

y' 0.0

y" 0.0

y-NH3 0.0

y-H2O 0.0

linker fragmentation

link rule link score matching psd rule(s)

-------------------------------------------------------------------------------

egs 1K671-2K653 1.5 legs11

detailed list of psd assignments

number of unassigned psd peaks 16

number of psd peaks assigned 3

number of psd fragments assigned 4

number of psd assignments 4

total score 7.5

m(exp) m(calc) d[Da] score psd rule fragmentation site(s)

-------------------------------------------------------------------------------

634.200 634.333 0.1 1.5 legs11 [1K671]-2K653

893.267 893.462 0.2 2.0 y1 2G652-[2K653]

1290.533 1291.678 1.1 2.0 y3 1N668-[1L669]

1290.533 1291.678 1.1 2.0 y5 2N648-[2V649]

*** legend ********************************************************************

peptide 42

number of base peptides = 2

number of missed cleavages = 0

number of optional links = 1

number of known links = 0

number of optional modifications = 0

number of known modifications = 0

calculated mass = 1273.6526

...............................................................................

isomer 1 / peptide 42

oooooooooooooooooo

2N648--2K653 2E1214--2K1216

. . . . . . . . . . . . . . . . . . . . . . . . . . . . . . . . . . . . . . . .

psd analysis for psd peak list "7" (selected parent mass = 1273.996)

concise lists of psd assignments

simple chain fragmentation

no assignments of simple chain fragments

linker fragmentation

link rule link score matching psd rule(s)

-------------------------------------------------------------------------------

egs 2K653-2K1216 1.5 legs6

detailed list of psd assignments

number of unassigned psd peaks 26

number of psd peaks assigned 1

number of psd fragments assigned 1

number of psd assignments 1

total score 1.5

m(exp) m(calc) d[Da] score psd rule fragmentation site(s)

-------------------------------------------------------------------------------

683.733 685.352 1.6 1.5 legs6 [2K653]-2K1216

*** legend ********************************************************************

peptide 43

number of base peptides = 2

number of missed cleavages = 0

number of optional links = 1

number of known links = 0

number of optional modifications = 0

number of known modifications = 0

calculated mass = 1405.7213

...............................................................................

isomer 1 / peptide 43

oooooooooooooooo

1N648--1K653 1N668--1K671

. . . . . . . . . . . . . . . . . . . . . . . . . . . . . . . . . . . . . . . .

psd analysis for psd peak list "8" (selected parent mass = 1405.492)

concise lists of psd assignments

simple chain fragmentation

chain 1 1 1 1

----- - - - -

6 6 6 6

4 5 6 7

8 3 8 1

rule N V E I G K N L F K score

a 0.0

b 0.0

b' 0.0

b" 0.0

b-NH3 0.0

b-H2O 0.0

b(n-1)+H2O 0.0

y 5 1 3 6.0

y' 0.0

y" 0.0

y-NH3 0.0

y-H2O 0.0

linker fragmentation

link rule link score matching psd rule(s)

-------------------------------------------------------------------------------

egs 1K653-1K671 1.5 regs11

detailed list of psd assignments

number of unassigned psd peaks 16

number of psd peaks assigned 3

number of psd fragments assigned 4

number of psd assignments 4

total score 7.5

m(exp) m(calc) d[Da] score psd rule fragmentation site(s)

-------------------------------------------------------------------------------

634.200 634.333 0.1 1.5 regs11 1K653-[1K671]

893.267 893.462 0.2 2.0 y1 1G652-[1K653]

1290.533 1291.678 1.1 2.0 y5 1N648-[1V649]

1290.533 1291.678 1.1 2.0 y3 1N668-[1L669]

*** legend ********************************************************************

peptide 44

number of base peptides = 2

number of missed cleavages = 0

number of optional links = 1

number of known links = 0

number of optional modifications = 0

number of known modifications = 0

calculated mass = 1273.6526

...............................................................................

isomer 1 / peptide 44

oooooooooooooooooo

1N648--1K653 2E1214--2K1216

. . . . . . . . . . . . . . . . . . . . . . . . . . . . . . . . . . . . . . . .

psd analysis for psd peak list "7" (selected parent mass = 1273.996)

concise lists of psd assignments

simple chain fragmentation

no assignments of simple chain fragments

linker fragmentation

link rule link score matching psd rule(s)

-------------------------------------------------------------------------------

egs 1K653-2K1216 1.5 legs6

detailed list of psd assignments

number of unassigned psd peaks 26

number of psd peaks assigned 1

number of psd fragments assigned 1

number of psd assignments 1

total score 1.5

m(exp) m(calc) d[Da] score psd rule fragmentation site(s)

-------------------------------------------------------------------------------

683.733 685.352 1.6 1.5 legs6 [1K653]-2K1216

*** legend ********************************************************************

peptide 45

number of base peptides = 2

number of missed cleavages = 0

number of optional links = 1

number of known links = 0

number of optional modifications = 0

number of known modifications = 0

calculated mass = 1405.7213

...............................................................................

isomer 1 / peptide 45

oooooooooooooooo

1N648--1K653 2N668--2K671

. . . . . . . . . . . . . . . . . . . . . . . . . . . . . . . . . . . . . . . .

psd analysis for psd peak list "8" (selected parent mass = 1405.492)

concise lists of psd assignments

simple chain fragmentation

chain 1 1 2 2

----- - - - -

6 6 6 6

4 5 6 7

8 3 8 1

rule N V E I G K N L F K score

a 0.0

b 0.0

b' 0.0

b" 0.0

b-NH3 0.0

b-H2O 0.0

b(n-1)+H2O 0.0

y 5 1 3 6.0

y' 0.0

y" 0.0

y-NH3 0.0

y-H2O 0.0

linker fragmentation

link rule link score matching psd rule(s)

-------------------------------------------------------------------------------

egs 1K653-2K671 1.5 regs11

detailed list of psd assignments

number of unassigned psd peaks 16

number of psd peaks assigned 3

number of psd fragments assigned 4

number of psd assignments 4

total score 7.5

m(exp) m(calc) d[Da] score psd rule fragmentation site(s)

-------------------------------------------------------------------------------

634.200 634.333 0.1 1.5 regs11 1K653-[2K671]

893.267 893.462 0.2 2.0 y1 1G652-[1K653]

1290.533 1291.678 1.1 2.0 y5 1N648-[1V649]

1290.533 1291.678 1.1 2.0 y3 2N668-[2L669]

*** legend ********************************************************************

peptide 46

number of base peptides = 2

number of missed cleavages = 0

number of optional links = 1

number of known links = 0

number of optional modifications = 0

number of known modifications = 0

calculated mass = 1251.6471

...............................................................................

isomer 1 / peptide 46

oooooooooooooooo

1F698--1K703 1Q866--1K868

. . . . . . . . . . . . . . . . . . . . . . . . . . . . . . . . . . . . . . . .

psd analysis for psd peak list "1" (selected parent mass = 1251.934)

concise lists of psd assignments

simple chain fragmentation

chain 1 1 1 1

----- - - - -

6 7 8 8

9 0 6 6

8 3 6 8

rule F S S I G K Q L K score

a 2 2.0

b 4 2 4.0

b' 0.0

b" 0.0

b-NH3 0.0

b-H2O 0.0

b(n-1)+H2O 0.0

y 2 2.0

y' 0.0

y" 0.0

y-NH3 0.0

y-H2O 1 2.0

linker fragmentation

link rule link score matching psd rule(s)

-------------------------------------------------------------------------------

egs 1K703-1K868 1.5 regs12

detailed list of psd assignments

number of unassigned psd peaks 9

number of psd peaks assigned 6

number of psd fragments assigned 6

number of psd assignments 6

total score 11.5

m(exp) m(calc) d[Da] score psd rule fragmentation site(s)

-------------------------------------------------------------------------------

214.840 214.156 0.7 2.0 a2 [1L867]-1K868

243.000 242.150 0.8 2.0 b2 [1L867]-1K868

436.000 435.224 0.8 2.0 b4 [1I701]-1G702

516.277 515.295 1.0 1.5 regs12 1K703-[1K868]

815.586 817.431 1.8 2.0 y2 1I701-[1G702]

992.283 992.494 0.2 2.0 y-H2O1 1L867-[1K868]

*** legend ********************************************************************

peptide 47

number of base peptides = 2

number of missed cleavages = 0

number of optional links = 1

number of known links = 0

number of optional modifications = 0

number of known modifications = 0

calculated mass = 1251.6471

...............................................................................

isomer 1 / peptide 47

oooooooooooooooo

1F698--1K703 2Q866--2K868

. . . . . . . . . . . . . . . . . . . . . . . . . . . . . . . . . . . . . . . .

psd analysis for psd peak list "1" (selected parent mass = 1251.934)

concise lists of psd assignments

simple chain fragmentation

chain 1 1 2 2

----- - - - -

6 7 8 8

9 0 6 6

8 3 6 8

rule F S S I G K Q L K score

a 2 2.0

b 4 2 4.0

b' 0.0

b" 0.0

b-NH3 0.0

b-H2O 0.0

b(n-1)+H2O 0.0

y 2 2.0

y' 0.0

y" 0.0

y-NH3 0.0

y-H2O 1 2.0

linker fragmentation

link rule link score matching psd rule(s)

-------------------------------------------------------------------------------

egs 1K703-2K868 1.5 regs12

detailed list of psd assignments

number of unassigned psd peaks 9

number of psd peaks assigned 6

number of psd fragments assigned 6

number of psd assignments 6

total score 11.5

m(exp) m(calc) d[Da] score psd rule fragmentation site(s)

-------------------------------------------------------------------------------

214.840 214.156 0.7 2.0 a2 [2L867]-2K868

243.000 242.150 0.8 2.0 b2 [2L867]-2K868

436.000 435.224 0.8 2.0 b4 [1I701]-1G702

516.277 515.295 1.0 1.5 regs12 1K703-[2K868]

815.586 817.431 1.8 2.0 y2 1I701-[1G702]

992.283 992.494 0.2 2.0 y-H2O1 2L867-[2K868]

*** legend ********************************************************************

peptide 48

number of base peptides = 2

number of missed cleavages = 0

number of optional links = 1

number of known links = 0

number of optional modifications = 0

number of known modifications = 0

calculated mass = 1295.6733

...............................................................................

isomer 1 / peptide 48

ooooooooooooooo

1F698--1K703 2A12--2K15

. . . . . . . . . . . . . . . . . . . . . . . . . . . . . . . . . . . . . . . .

psd analysis for psd peak list "5" (selected parent mass = 1295.914)

concise lists of psd assignments

simple chain fragmentation

no assignments of simple chain fragments

linker fragmentation

link rule link score matching psd rule(s)

-------------------------------------------------------------------------------

egs 1K703-2K15 1.5 legs6

detailed list of psd assignments

number of unassigned psd peaks 22

number of psd peaks assigned 1

number of psd fragments assigned 1

number of psd assignments 1

total score 1.5

m(exp) m(calc) d[Da] score psd rule fragmentation site(s)

-------------------------------------------------------------------------------

665.067 664.331 0.7 1.5 legs6 [1K703]-2K15

*** legend ********************************************************************

peptide 49

number of base peptides = 2

number of missed cleavages = 0

number of optional links = 1

number of known links = 0

number of optional modifications = 0

number of known modifications = 0

calculated mass = 1295.6733

...............................................................................

isomer 1 / peptide 49

ooooooooooooooo

1A12--1K15 1F698--1K703

. . . . . . . . . . . . . . . . . . . . . . . . . . . . . . . . . . . . . . . .

psd analysis for psd peak list "5" (selected parent mass = 1295.914)

concise lists of psd assignments

simple chain fragmentation

no assignments of simple chain fragments

linker fragmentation

link rule link score matching psd rule(s)

-------------------------------------------------------------------------------

egs 1K15-1K703 1.5 regs6

detailed list of psd assignments

number of unassigned psd peaks 22

number of psd peaks assigned 1

number of psd fragments assigned 1

number of psd assignments 1

total score 1.5

m(exp) m(calc) d[Da] score psd rule fragmentation site(s)

-------------------------------------------------------------------------------

665.067 664.331 0.7 1.5 regs6 1K15-[1K703]

*** legend ********************************************************************

peptide 50

number of base peptides = 2

number of missed cleavages = 0

number of optional links = 1

number of known links = 0

number of optional modifications = 0

number of known modifications = 0

calculated mass = 1273.6025

...............................................................................

isomer 1 / peptide 50

oooooooooooooooo

1M500--1K502 2S967--2K971

. . . . . . . . . . . . . . . . . . . . . . . . . . . . . . . . . . . . . . . .

psd analysis for psd peak list "7" (selected parent mass = 1273.996)

concise lists of psd assignments

simple chain fragmentation

chain 1 1 2 2

----- - - - -

5 5 9 9

0 0 6 7

0 2 7 1

rule M F K S L F E K score

a 0.0

b 0.0

b' 0.0

b" 0.0

b-NH3 0.0

b-H2O 0.0

b(n-1)+H2O 0.0

y 4 2.0

y' 0.0

y" 0.0

y-NH3 0.0

y-H2O 2 2.0

linker fragmentation

link rule link score matching psd rule(s)

-------------------------------------------------------------------------------

egs 1K502-2K971 4.5 legs7,legs8,legs9

detailed list of psd assignments

number of unassigned psd peaks 22

number of psd peaks assigned 5

number of psd fragments assigned 5

number of psd assignments 5

total score 8.5

m(exp) m(calc) d[Da] score psd rule fragmentation site(s)

-------------------------------------------------------------------------------

464.500 466.225 1.7 1.5 legs7 [1K502]-2K971

479.500 480.241 0.7 1.5 legs8 [1K502]-2K971

507.467 507.228 0.2 1.5 legs9 [1K502]-2K971

1124.500 1124.551 0.1 2.0 y-H2O2 1M500-[1F501]

1186.733 1186.570 0.2 2.0 y4 2S967-[2L968]

*** legend ********************************************************************

peptide 51

number of base peptides = 2

number of missed cleavages = 0

number of optional links = 1

number of known links = 0

number of optional modifications = 0

number of known modifications = 0

calculated mass = 1405.6638

...............................................................................

isomer 1 / peptide 51

oooooooooooooooo

1S1067--1K1070 2S967--2K971

. . . . . . . . . . . . . . . . . . . . . . . . . . . . . . . . . . . . . . . .

psd analysis for psd peak list "8" (selected parent mass = 1405.492)

concise lists of psd assignments

simple chain fragmentation

chain 1 1 2 2

----- - - - -

1 1 0 0

0 0 9 9

6 7 6 7

7 0 7 1

rule S W H K S L F E K score

a 0.0

b 0.0

b' 0.0

b" 0.0

b-NH3 0.0

b-H2O 4 2.0

b(n-1)+H2O 0.0

y 1 2.0

y' 1 1.5

y" 0.0

y-NH3 0.0

y-H2O 1 2.0

linker fragmentation

link rule link score matching psd rule(s)

-------------------------------------------------------------------------------

egs 1K1070-2K971 1.5 regs12

detailed list of psd assignments

number of unassigned psd peaks 15

number of psd peaks assigned 4

number of psd fragments assigned 5

number of psd assignments 5

total score 9.0

m(exp) m(calc) d[Da] score psd rule fragmentation site(s)

-------------------------------------------------------------------------------

461.000 459.224 1.8 2.0 b-H2O4 [2E970]-2K971

750.400 750.380 0.0 1.5 regs12 1K1070-[2K971]

931.400 929.437 2.0 2.0 y1 2E970-[2K971]

931.400 929.437 2.0 1.5 y'1 2E970-[2K971]

979.400 977.483 1.9 2.0 y-H2O1 1H1069-[1K1070]

*** legend ********************************************************************

peptide 52

number of base peptides = 3

number of missed cleavages = 1

number of optional links = 1

number of known links = 0

number of optional modifications = 0

number of known modifications = 0

calculated mass = 1418.6914

...............................................................................

isomer 1 / peptide 52

oooooooooo

1A284--1K288 1K1229 ~ 1G1230--1R1233

. . . . . . . . . . . . . . . . . . . . . . . . . . . . . . . . . . . . . . . .

psd analysis for psd peak list "4" (selected parent mass = 1418.573)

concise lists of psd assignments

simple chain fragmentation

chain 1 1 1 1

----- - - - -

0 0 1 1

2 2 2 2

8 8 2 3

4 8 9 3

rule A S W N K K G L D R score

a 0.0

b 0.0

b' 0.0

b" 0.0

b-NH3 0.0

b-H2O 4 2.0

b(n-1)+H2O 0.0

y 4 3 2 1 30.0

y' 0.0

y" 0.0

y-NH3 4 3 2 1 4 32.0

y-H2O 4 2.0

linker fragmentation

link rule link score matching psd rule(s)

-------------------------------------------------------------------------------

egs 1K288-1K1229 4.5 legs7,legs8,legs12

detailed list of psd assignments

number of unassigned psd peaks 29

number of psd peaks assigned 12

number of psd fragments assigned 14

number of psd assignments 14

total score 70.5

m(exp) m(calc) d[Da] score psd rule fragmentation site(s)

-------------------------------------------------------------------------------

441.892 441.189 0.7 2.0 b-H2O4 [1N287]-1K288

441.892 443.225 1.3 2.0 y-NH34 1K1229-[1G1230]

645.500 646.307 0.8 1.5 legs7 [1K288]-1K1229

659.038 660.323 1.3 1.5 legs8 [1K288]-1K1229

733.500 732.344 1.2 1.5 legs12 [1K288]-1K1229

943.500 943.474 0.0 16.0 y-NH31 1N287-[1K288]

961.500 960.500 1.0 16.0 y1 1N287-[1K288]

1056.000 1057.516 1.5 8.0 y-NH32 1W286-[1N287]

1074.438 1074.543 0.1 8.0 y2 1W286-[1N287]

1242.000 1243.596 1.6 4.0 y-NH33 1S285-[1W286]

1259.651 1260.622 1.0 4.0 y3 1S285-[1W286]

1330.503 1329.644 0.9 2.0 y-H2O4 1A284-[1S285]

1330.503 1330.628 0.1 2.0 y-NH34 1A284-[1S285]

1347.500 1347.654 0.2 2.0 y4 1A284-[1S285]

*** legend ********************************************************************

peptide 53

number of base peptides = 3

number of missed cleavages = 1

number of optional links = 1

number of known links = 0

number of optional modifications = 0

number of known modifications = 0

calculated mass = 1418.7278

...............................................................................

isomer 1 / peptide 53

oooooooooooooooooooooo

1A12--1K15 ~ 1R16 1A284--1K288

. . . . . . . . . . . . . . . . . . . . . . . . . . . . . . . . . . . . . . . .

psd analysis for psd peak list "4" (selected parent mass = 1418.573)

concise lists of psd assignments

simple chain fragmentation

chain 1 1 1 1

----- - - - -

0 0 2 2

1 1 8 8

2 6 4 8

rule A T I K R A S W N K score

a 0.0

b 0.0

b' 0.0

b" 0.0

b-NH3 0.0

b-H2O 4 2.0

b(n-1)+H2O 0.0

y 4 3 2 4 3 2 1 44.0

y' 0.0

y" 0.0

y-NH3 4 3 2 4 3 2 1 44.0

y-H2O 4 3 4 3 2 1 36.0

linker fragmentation

link rule link score matching psd rule(s)

-------------------------------------------------------------------------------

egs 1K15-1K288 4.5 regs7,regs8,regs12

detailed list of psd assignments

number of unassigned psd peaks 25

number of psd peaks assigned 16

number of psd fragments assigned 24

number of psd assignments 24

total score 130.5

m(exp) m(calc) d[Da] score psd rule fragmentation site(s)

-------------------------------------------------------------------------------

441.892 441.189 0.7 2.0 b-H2O4 [1N287]-1K288

645.500 646.307 0.8 1.5 regs7 1K15-[1K288]

659.038 660.323 1.3 1.5 regs8 1K15-[1K288]

733.500 732.344 1.2 1.5 regs12 1K15-[1K288]

943.500 942.526 1.0 16.0 y-H2O1 1N287-[1K288]

943.500 943.510 0.0 16.0 y-NH31 1N287-[1K288]

961.500 960.536 1.0 16.0 y1 1N287-[1K288]

1056.000 1056.569 0.6 8.0 y-H2O2 1W286-[1N287]

1056.000 1057.553 1.6 8.0 y-NH32 1W286-[1N287]

1074.438 1074.579 0.1 8.0 y2 1W286-[1N287]

1118.346 1116.532 1.8 8.0 y-NH32 1I14-[1K15]

1134.500 1133.559 0.9 8.0 y2 1I14-[1K15]

1230.000 1228.632 1.4 4.0 y-H2O3 1T13-[1I14]

1230.000 1229.616 0.4 4.0 y-NH33 1T13-[1I14]

1242.000 1242.648 0.6 4.0 y-H2O3 1S285-[1W286]

1242.000 1243.632 1.6 4.0 y-NH33 1S285-[1W286]

1247.500 1246.643 0.9 4.0 y3 1T13-[1I14]

1259.651 1260.659 1.0 4.0 y3 1S285-[1W286]

1330.503 1329.680 0.8 2.0 y-H2O4 1A12-[1T13]

1330.503 1329.680 0.8 2.0 y-H2O4 1A284-[1S285]

1330.503 1330.664 0.2 2.0 y-NH34 1A12-[1T13]

1330.503 1330.664 0.2 2.0 y-NH34 1A284-[1S285]

1347.500 1347.691 0.2 2.0 y4 1A12-[1T13]

1347.500 1347.691 0.2 2.0 y4 1A284-[1S285]

*** legend ********************************************************************

peptide 54

number of base peptides = 3

number of missed cleavages = 1

number of optional links = 1

number of known links = 0

number of optional modifications = 0

number of known modifications = 0

calculated mass = 1418.7278

...............................................................................

isomer 1 / peptide 54

ooooooooooooooo

1A284--1K288 2A12--2K15 ~ 2R16

. . . . . . . . . . . . . . . . . . . . . . . . . . . . . . . . . . . . . . . .

psd analysis for psd peak list "4" (selected parent mass = 1418.573)

concise lists of psd assignments

simple chain fragmentation

chain 1 1 2 2

----- - - - -

2 2 0 0

8 8 1 1

4 8 2 6

rule A S W N K A T I K R score

a 0.0

b 0.0

b' 0.0

b" 0.0

b-NH3 0.0

b-H2O 4 2.0

b(n-1)+H2O 0.0

y 4 3 2 1 4 3 2 44.0

y' 0.0

y" 0.0

y-NH3 4 3 2 1 4 3 2 44.0

y-H2O 4 3 2 1 4 3 36.0

linker fragmentation

link rule link score matching psd rule(s)

-------------------------------------------------------------------------------

egs 1K288-2K15 4.5 legs7,legs8,legs12

detailed list of psd assignments

number of unassigned psd peaks 25

number of psd peaks assigned 16

number of psd fragments assigned 24

number of psd assignments 24

total score 130.5

m(exp) m(calc) d[Da] score psd rule fragmentation site(s)

-------------------------------------------------------------------------------

441.892 441.189 0.7 2.0 b-H2O4 [1N287]-1K288

645.500 646.307 0.8 1.5 legs7 [1K288]-2K15

659.038 660.323 1.3 1.5 legs8 [1K288]-2K15

733.500 732.344 1.2 1.5 legs12 [1K288]-2K15

943.500 942.526 1.0 16.0 y-H2O1 1N287-[1K288]

943.500 943.510 0.0 16.0 y-NH31 1N287-[1K288]

961.500 960.536 1.0 16.0 y1 1N287-[1K288]

1056.000 1056.569 0.6 8.0 y-H2O2 1W286-[1N287]

1056.000 1057.553 1.6 8.0 y-NH32 1W286-[1N287]

1074.438 1074.579 0.1 8.0 y2 1W286-[1N287]

1118.346 1116.532 1.8 8.0 y-NH32 2I14-[2K15]

1134.500 1133.559 0.9 8.0 y2 2I14-[2K15]

1230.000 1228.632 1.4 4.0 y-H2O3 2T13-[2I14]

1230.000 1229.616 0.4 4.0 y-NH33 2T13-[2I14]

1242.000 1242.648 0.6 4.0 y-H2O3 1S285-[1W286]

1242.000 1243.632 1.6 4.0 y-NH33 1S285-[1W286]

1247.500 1246.643 0.9 4.0 y3 2T13-[2I14]

1259.651 1260.659 1.0 4.0 y3 1S285-[1W286]

1330.503 1329.680 0.8 2.0 y-H2O4 1A284-[1S285]

1330.503 1329.680 0.8 2.0 y-H2O4 2A12-[2T13]

1330.503 1330.664 0.2 2.0 y-NH34 1A284-[1S285]

1330.503 1330.664 0.2 2.0 y-NH34 2A12-[2T13]

1347.500 1347.691 0.2 2.0 y4 1A284-[1S285]

1347.500 1347.691 0.2 2.0 y4 2A12-[2T13]

*** legend ********************************************************************

peptide 55

number of base peptides = 3

number of missed cleavages = 1

number of optional links = 1

number of known links = 0

number of optional modifications = 0

number of known modifications = 0

calculated mass = 1418.6914

...............................................................................

isomer 1 / peptide 55

oooooooooo

1A284--1K288 2K1229 ~ 2G1230--2R1233

. . . . . . . . . . . . . . . . . . . . . . . . . . . . . . . . . . . . . . . .

psd analysis for psd peak list "4" (selected parent mass = 1418.573)

concise lists of psd assignments

simple chain fragmentation

chain 1 1 2 2

----- - - - -

0 0 1 1

2 2 2 2

8 8 2 3

4 8 9 3

rule A S W N K K G L D R score

a 0.0

b 0.0

b' 0.0

b" 0.0

b-NH3 0.0

b-H2O 4 2.0

b(n-1)+H2O 0.0

y 4 3 2 1 30.0

y' 0.0

y" 0.0

y-NH3 4 3 2 1 4 32.0

y-H2O 4 2.0

linker fragmentation

link rule link score matching psd rule(s)

-------------------------------------------------------------------------------

egs 1K288-2K1229 4.5 legs7,legs8,legs12

detailed list of psd assignments

number of unassigned psd peaks 29

number of psd peaks assigned 12

number of psd fragments assigned 14

number of psd assignments 14

total score 70.5

m(exp) m(calc) d[Da] score psd rule fragmentation site(s)

-------------------------------------------------------------------------------

441.892 441.189 0.7 2.0 b-H2O4 [1N287]-1K288

441.892 443.225 1.3 2.0 y-NH34 2K1229-[2G1230]

645.500 646.307 0.8 1.5 legs7 [1K288]-2K1229

659.038 660.323 1.3 1.5 legs8 [1K288]-2K1229

733.500 732.344 1.2 1.5 legs12 [1K288]-2K1229

943.500 943.474 0.0 16.0 y-NH31 1N287-[1K288]

961.500 960.500 1.0 16.0 y1 1N287-[1K288]

1056.000 1057.516 1.5 8.0 y-NH32 1W286-[1N287]

1074.438 1074.543 0.1 8.0 y2 1W286-[1N287]

1242.000 1243.596 1.6 4.0 y-NH33 1S285-[1W286]

1259.651 1260.622 1.0 4.0 y3 1S285-[1W286]

1330.503 1329.644 0.9 2.0 y-H2O4 1A284-[1S285]

1330.503 1330.628 0.1 2.0 y-NH34 1A284-[1S285]

1347.500 1347.654 0.2 2.0 y4 1A284-[1S285]

*** legend ********************************************************************

peptide 56

number of base peptides = 3

number of missed cleavages = 1

number of optional links = 1

number of known links = 0

number of optional modifications = 0

number of known modifications = 0

calculated mass = 1418.7278

...............................................................................

isomer 1 / peptide 56

ooooooooooooooooooooooooooooooo

1A480--1K484 ~ 1A485--1R486 2Q866--2K868

. . . . . . . . . . . . . . . . . . . . . . . . . . . . . . . . . . . . . . . .

psd analysis for psd peak list "4" (selected parent mass = 1418.573)

concise lists of psd assignments

simple chain fragmentation

chain 1 1 2 2

----- - - - -

4 4 8 8

8 8 6 6

0 6 6 8

rule A S W S K A R Q L K score

a 0.0

b 0.0

b' 0.0

b" 0.0

b-NH3 0.0

b-H2O 0.0

b(n-1)+H2O 0.0

y 6 5 4 14.0

y' 0.0

y" 0.0

y-NH3 6 5 4 14.0

y-H2O 6 5 4 14.0

linker fragmentation

link rule link score matching psd rule(s)

-------------------------------------------------------------------------------

egs 1K484-2K868 3.0 legs8,regs8

detailed list of psd assignments

number of unassigned psd peaks 32

number of psd peaks assigned 9

number of psd fragments assigned 11

number of psd assignments 12

total score 45.0

m(exp) m(calc) d[Da] score psd rule fragmentation site(s)

-------------------------------------------------------------------------------

441.892 443.274 1.4 1.5 regs8 1K484-[2K868]

859.087 860.450 1.4 1.5 legs8 [1K484]-2K868

861.083 860.450 0.6 1.5 legs8 [1K484]-2K868

1056.000 1056.569 0.6 8.0 y-H2O4 1W482-[1S483]

1056.000 1057.553 1.6 8.0 y-NH34 1W482-[1S483]

1074.438 1074.579 0.1 8.0 y4 1W482-[1S483]

1242.000 1242.648 0.6 4.0 y-H2O5 1S481-[1W482]

1242.000 1243.632 1.6 4.0 y-NH35 1S481-[1W482]

1259.651 1260.659 1.0 4.0 y5 1S481-[1W482]

1330.503 1329.680 0.8 2.0 y-H2O6 1A480-[1S481]

1330.503 1330.664 0.2 2.0 y-NH36 1A480-[1S481]

1347.500 1347.691 0.2 2.0 y6 1A480-[1S481]

*** legend ********************************************************************

peptide 57

number of base peptides = 3

number of missed cleavages = 1

number of optional links = 1

number of known links = 0

number of optional modifications = 0

number of known modifications = 0

calculated mass = 1418.7278

...............................................................................

isomer 1 / peptide 57

ooooooooooooooooooooooooooooooo

1A480--1K484 ~ 1A485--1R486 1Q866--1K868

. . . . . . . . . . . . . . . . . . . . . . . . . . . . . . . . . . . . . . . .

psd analysis for psd peak list "4" (selected parent mass = 1418.573)

concise lists of psd assignments

simple chain fragmentation

chain 1 1 1 1

----- - - - -

4 4 8 8

8 8 6 6

0 6 6 8

rule A S W S K A R Q L K score

a 0.0

b 0.0

b' 0.0

b" 0.0

b-NH3 0.0

b-H2O 0.0

b(n-1)+H2O 0.0

y 6 5 4 14.0

y' 0.0

y" 0.0

y-NH3 6 5 4 14.0

y-H2O 6 5 4 14.0

linker fragmentation

link rule link score matching psd rule(s)

-------------------------------------------------------------------------------

egs 1K484-1K868 3.0 legs8,regs8

detailed list of psd assignments

number of unassigned psd peaks 32

number of psd peaks assigned 9

number of psd fragments assigned 11

number of psd assignments 12

total score 45.0

m(exp) m(calc) d[Da] score psd rule fragmentation site(s)

-------------------------------------------------------------------------------

441.892 443.274 1.4 1.5 regs8 1K484-[1K868]

859.087 860.450 1.4 1.5 legs8 [1K484]-1K868

861.083 860.450 0.6 1.5 legs8 [1K484]-1K868

1056.000 1056.569 0.6 8.0 y-H2O4 1W482-[1S483]

1056.000 1057.553 1.6 8.0 y-NH34 1W482-[1S483]

1074.438 1074.579 0.1 8.0 y4 1W482-[1S483]

1242.000 1242.648 0.6 4.0 y-H2O5 1S481-[1W482]

1242.000 1243.632 1.6 4.0 y-NH35 1S481-[1W482]

1259.651 1260.659 1.0 4.0 y5 1S481-[1W482]

1330.503 1329.680 0.8 2.0 y-H2O6 1A480-[1S481]

1330.503 1330.664 0.2 2.0 y-NH36 1A480-[1S481]

1347.500 1347.691 0.2 2.0 y6 1A480-[1S481]

*** legend ********************************************************************

peptide 58

number of base peptides = 3

number of missed cleavages = 1

number of optional links = 1

number of known links = 0

number of optional modifications = 0

number of known modifications = 0

calculated mass = 1405.7326

...............................................................................

isomer 1 / peptide 58

oooooooooooooooo

1R654 ~ 1V655--1K658 1N753--1K757

. . . . . . . . . . . . . . . . . . . . . . . . . . . . . . . . . . . . . . . .

psd analysis for psd peak list "8" (selected parent mass = 1405.492)

concise lists of psd assignments

simple chain fragmentation

chain 1 1 1 1

----- - - - -

6 6 7 7

5 5 5 5

4 8 3 7

rule R V F A K N G L E K score

a 0.0

b 0.0

b' 0.0

b" 0.0

b-NH3 0.0

b-H2O 0.0

b(n-1)+H2O 0.0

y 1 4 4.0

y' 0.0

y" 0.0

y-NH3 0.0

y-H2O 0.0

linker fragmentation

link rule link score matching psd rule(s)

-------------------------------------------------------------------------------

egs 1K658-1K757 3.0 legs9,regs5

detailed list of psd assignments

number of unassigned psd peaks 15

number of psd peaks assigned 4

number of psd fragments assigned 4

number of psd assignments 4

total score 7.0

m(exp) m(calc) d[Da] score psd rule fragmentation site(s)

-------------------------------------------------------------------------------

559.400 560.304 0.9 1.5 regs5 1K658-[1K757]

703.333 702.394 0.9 1.5 legs9 [1K658]-1K757

931.400 932.458 1.1 2.0 y1 1A657-[1K658]

1290.533 1291.690 1.2 2.0 y4 1N753-[1G754]

*** legend ********************************************************************

peptide 59

number of base peptides = 3

number of missed cleavages = 1

number of optional links = 1

number of known links = 0

number of optional modifications = 0

number of known modifications = 0

calculated mass = 1273.6638

...............................................................................

isomer 1 / peptide 59

oooooooooo

1N753--1K757 2K1350 ~ 2V1351--2K1353

. . . . . . . . . . . . . . . . . . . . . . . . . . . . . . . . . . . . . . . .

psd analysis for psd peak list "7" (selected parent mass = 1273.996)

concise lists of psd assignments

simple chain fragmentation

no assignments of simple chain fragments

linker fragmentation

link rule link score matching psd rule(s)

-------------------------------------------------------------------------------

egs 1K757-2K1350 3.0 legs15,regs14

detailed list of psd assignments

number of unassigned psd peaks 25

number of psd peaks assigned 2

number of psd fragments assigned 2

number of psd assignments 2

total score 3.0

m(exp) m(calc) d[Da] score psd rule fragmentation site(s)

-------------------------------------------------------------------------------

656.667 658.341 1.7 1.5 regs14 1K757-[2K1350]

744.333 745.349 1.0 1.5 legs15 [1K757]-2K1350

...............................................................................

isomer 2 / peptide 59

ooooooooooooooooooooooooooo

1N753--1K757 2K1350 ~ 2V1351--2K1353

. . . . . . . . . . . . . . . . . . . . . . . . . . . . . . . . . . . . . . . .

psd analysis for psd peak list "7" (selected parent mass = 1273.996)

concise lists of psd assignments

simple chain fragmentation

no assignments of simple chain fragments

linker fragmentation

link rule link score matching psd rule(s)

-------------------------------------------------------------------------------

egs 1K757-2K1353 3.0 legs15,regs14

detailed list of psd assignments

number of unassigned psd peaks 25

number of psd peaks assigned 2

number of psd fragments assigned 2

number of psd assignments 2

total score 3.0

m(exp) m(calc) d[Da] score psd rule fragmentation site(s)

-------------------------------------------------------------------------------

656.667 658.341 1.7 1.5 regs14 1K757-[2K1353]

744.333 745.349 1.0 1.5 legs15 [1K757]-2K1353

*** legend ********************************************************************

peptide 60

number of base peptides = 3

number of missed cleavages = 1

number of optional links = 1

number of known links = 0

number of optional modifications = 0

number of known modifications = 0

calculated mass = 1405.7326

...............................................................................

isomer 1 / peptide 60

oooooooooooooooooooooooo

1N753--1K757 2R654 ~ 2V655--2K658

. . . . . . . . . . . . . . . . . . . . . . . . . . . . . . . . . . . . . . . .

psd analysis for psd peak list "8" (selected parent mass = 1405.492)

concise lists of psd assignments

simple chain fragmentation

chain 1 1 2 2

----- - - - -

7 7 6 6

5 5 5 5

3 7 4 8

rule N G L E K R V F A K score

a 0.0

b 0.0

b' 0.0

b" 0.0

b-NH3 0.0

b-H2O 0.0

b(n-1)+H2O 0.0

y 4 1 4.0

y' 0.0

y" 0.0

y-NH3 0.0

y-H2O 0.0

linker fragmentation

link rule link score matching psd rule(s)

-------------------------------------------------------------------------------

egs 1K757-2K658 3.0 legs5,regs9

detailed list of psd assignments

number of unassigned psd peaks 15

number of psd peaks assigned 4

number of psd fragments assigned 4

number of psd assignments 4

total score 7.0

m(exp) m(calc) d[Da] score psd rule fragmentation site(s)

-------------------------------------------------------------------------------

559.400 560.304 0.9 1.5 legs5 [1K757]-2K658

703.333 702.394 0.9 1.5 regs9 1K757-[2K658]

931.400 932.458 1.1 2.0 y1 2A657-[2K658]

1290.533 1291.690 1.2 2.0 y4 1N753-[1G754]

*** legend ********************************************************************

peptide 61

number of base peptides = 3

number of missed cleavages = 1

number of optional links = 1

number of known links = 0

number of optional modifications = 0

number of known modifications = 0

calculated mass = 1418.7318

...............................................................................

isomer 1 / peptide 61

oooooooooo

1S1067--1K1070 1K1120 ~ 1I1121--1K1124

. . . . . . . . . . . . . . . . . . . . . . . . . . . . . . . . . . . . . . . .

psd analysis for psd peak list "4" (selected parent mass = 1418.573)

concise lists of psd assignments

simple chain fragmentation

chain 1 1 1 1

----- - - - -

1 1 1 1

0 0 1 1

6 7 2 2

7 0 0 4

rule S W H K K I F T K score

a 0.0

b 0.0

b' 0.0

b" 0.0

b-NH3 0.0

b-H2O 0.0

b(n-1)+H2O 0.0

y 3 1 4.0

y' 0.0

y" 0.0

y-NH3 0.0

y-H2O 4 2.0

linker fragmentation

link rule link score matching psd rule(s)

-------------------------------------------------------------------------------

egs 1K1070-1K1120 3.0 legs17,regs17

detailed list of psd assignments

number of unassigned psd peaks 36

number of psd peaks assigned 5

number of psd fragments assigned 5

number of psd assignments 5

total score 9.0

m(exp) m(calc) d[Da] score psd rule fragmentation site(s)

-------------------------------------------------------------------------------

489.193 490.303 1.1 2.0 y-H2O4 1K1120-[1I1121]

781.516 783.331 1.8 1.5 legs17 [1K1070]-1K1120

861.083 862.456 1.4 1.5 regs17 1K1070-[1K1120]

1008.509 1008.562 0.1 2.0 y1 1H1069-[1K1070]

1330.503 1331.700 1.2 2.0 y3 1S1067-[1W1068]

...............................................................................

isomer 2 / peptide 61

ooooooooooooooooooooooooooo

1S1067--1K1070 1K1120 ~ 1I1121--1K1124

. . . . . . . . . . . . . . . . . . . . . . . . . . . . . . . . . . . . . . . .

psd analysis for psd peak list "4" (selected parent mass = 1418.573)

concise lists of psd assignments

simple chain fragmentation

chain 1 1 1 1

----- - - - -

1 1 1 1

0 0 1 1

6 7 2 2

7 0 0 4

rule S W H K K I F T K score

a 0.0

b 4 2.0

b' 0.0

b" 0.0

b-NH3 0.0

b-H2O 0.0

b(n-1)+H2O 0.0

y 3 1 4.0

y' 0.0

y" 0.0

y-NH3 0.0

y-H2O 0.0

linker fragmentation

link rule link score matching psd rule(s)

-------------------------------------------------------------------------------

egs 1K1070-1K1124 3.0 legs17,regs17

detailed list of psd assignments

number of unassigned psd peaks 36

number of psd peaks assigned 5

number of psd fragments assigned 5

number of psd assignments 5

total score 9.0

m(exp) m(calc) d[Da] score psd rule fragmentation site(s)

-------------------------------------------------------------------------------

489.193 490.303 1.1 2.0 b4 [1T1123]-1K1124

781.516 783.331 1.8 1.5 legs17 [1K1070]-1K1124

861.083 862.456 1.4 1.5 regs17 1K1070-[1K1124]

1008.509 1008.562 0.1 2.0 y1 1H1069-[1K1070]

1330.503 1331.700 1.2 2.0 y3 1S1067-[1W1068]

*** legend ********************************************************************

peptide 62

number of base peptides = 3

number of missed cleavages = 1

number of optional links = 1

number of known links = 0

number of optional modifications = 0

number of known modifications = 0

calculated mass = 1295.6958

...............................................................................

isomer 1 / peptide 62

oooooooooooooooo

1Q866--1K868 2E721--2K724 ~ 2R725

. . . . . . . . . . . . . . . . . . . . . . . . . . . . . . . . . . . . . . . .

psd analysis for psd peak list "5" (selected parent mass = 1295.914)

concise lists of psd assignments

simple chain fragmentation

no assignments of simple chain fragments

linker fragmentation

link rule link score matching psd rule(s)

-------------------------------------------------------------------------------

egs 1K868-2K724 6.0 legs7,legs9,legs15,regs5

detailed list of psd assignments

number of unassigned psd peaks 19

number of psd peaks assigned 4

number of psd fragments assigned 4

number of psd assignments 4

total score 6.0

m(exp) m(calc) d[Da] score psd rule fragmentation site(s)

-------------------------------------------------------------------------------

428.600 429.259 0.7 1.5 legs7 [1K868]-2K724

470.067 470.261 0.2 1.5 legs9 [1K868]-2K724

571.500 573.301 1.8 1.5 legs15 [1K868]-2K724

683.467 682.400 1.1 1.5 regs5 1K868-[2K724]

*** legend ********************************************************************

peptide 63

number of base peptides = 3

number of missed cleavages = 1

number of optional links = 1

number of known links = 0

number of optional modifications = 0

number of known modifications = 0

calculated mass = 1295.6958

...............................................................................

isomer 1 / peptide 63

oooooooooooooooooooooooo

2E721--2K724 ~ 2R725 2Q866--2K868

. . . . . . . . . . . . . . . . . . . . . . . . . . . . . . . . . . . . . . . .

psd analysis for psd peak list "5" (selected parent mass = 1295.914)

concise lists of psd assignments

simple chain fragmentation

no assignments of simple chain fragments

linker fragmentation

link rule link score matching psd rule(s)

-------------------------------------------------------------------------------

egs 2K724-2K868 6.0 legs5,regs7,regs9,regs15

detailed list of psd assignments

number of unassigned psd peaks 19

number of psd peaks assigned 4

number of psd fragments assigned 4

number of psd assignments 4

total score 6.0

m(exp) m(calc) d[Da] score psd rule fragmentation site(s)

-------------------------------------------------------------------------------

428.600 429.259 0.7 1.5 regs7 2K724-[2K868]

470.067 470.261 0.2 1.5 regs9 2K724-[2K868]

571.500 573.301 1.8 1.5 regs15 2K724-[2K868]

683.467 682.400 1.1 1.5 legs5 [2K724]-2K868

*** legend ********************************************************************

peptide 64

number of base peptides = 3

number of missed cleavages = 1

number of optional links = 1

number of known links = 0

number of optional modifications = 0

number of known modifications = 0

calculated mass = 1541.8326

...............................................................................

isomer 1 / peptide 64

ooooooooooooooooooooooooooooooo

1V655--1K658 2L718--2R720 ~ 2E721--2K724

. . . . . . . . . . . . . . . . . . . . . . . . . . . . . . . . . . . . . . . .

psd analysis for psd peak list "2" (selected parent mass = 1542.509)

concise lists of psd assignments

simple chain fragmentation

chain 1 1 2 2

----- - - - -

6 6 7 7

5 5 1 2

5 8 8 4

rule V F A K L G R E H I K score

a 0.0

b 0.0

b' 0.0

b" 0.0

b-NH3 0.0

b-H2O 0.0

b(n-1)+H2O 0.0

y 3 6 2 6.0

y' 0.0

y" 0.0

y-NH3 3 6 5 8.0

y-H2O 0.0

linker fragmentation

no assignments of linker fragments

detailed list of psd assignments

number of unassigned psd peaks 41

number of psd peaks assigned 5

number of psd fragments assigned 6

number of psd assignments 6

total score 14.0

m(exp) m(calc) d[Da] score psd rule fragmentation site(s)

-------------------------------------------------------------------------------

951.333 949.525 1.8 2.0 y2 2H722-[2I723]

1355.533 1354.701 0.8 4.0 y-NH35 2G719-[2R720]

1411.400 1411.722 0.3 2.0 y-NH36 2L718-[2G719]

1427.600 1425.738 1.9 2.0 y-NH33 1V655-[1F656]

1427.600 1428.749 1.1 2.0 y6 2L718-[2G719]

1442.600 1442.764 0.2 2.0 y3 1V655-[1F656]

*** legend ********************************************************************

peptide 65

number of base peptides = 3

number of missed cleavages = 1

number of optional links = 1

number of known links = 0

number of optional modifications = 0

number of known modifications = 0

calculated mass = 1541.8326

...............................................................................

isomer 1 / peptide 65

ooooooooooooooooooooooooooooooo

2V655--2K658 2L718--2R720 ~ 2E721--2K724

. . . . . . . . . . . . . . . . . . . . . . . . . . . . . . . . . . . . . . . .

psd analysis for psd peak list "2" (selected parent mass = 1542.509)

concise lists of psd assignments

simple chain fragmentation

chain 2 2 2 2

----- - - - -

6 6 7 7

5 5 1 2

5 8 8 4

rule V F A K L G R E H I K score

a 0.0

b 0.0

b' 0.0

b" 0.0

b-NH3 0.0

b-H2O 0.0

b(n-1)+H2O 0.0

y 3 6 2 6.0

y' 0.0

y" 0.0

y-NH3 3 6 5 8.0

y-H2O 0.0

linker fragmentation

no assignments of linker fragments

detailed list of psd assignments

number of unassigned psd peaks 41

number of psd peaks assigned 5

number of psd fragments assigned 6

number of psd assignments 6

total score 14.0

m(exp) m(calc) d[Da] score psd rule fragmentation site(s)

-------------------------------------------------------------------------------

951.333 949.525 1.8 2.0 y2 2H722-[2I723]

1355.533 1354.701 0.8 4.0 y-NH35 2G719-[2R720]

1411.400 1411.722 0.3 2.0 y-NH36 2L718-[2G719]

1427.600 1425.738 1.9 2.0 y-NH33 2V655-[2F656]

1427.600 1428.749 1.1 2.0 y6 2L718-[2G719]

1442.600 1442.764 0.2 2.0 y3 2V655-[2F656]

*** legend ********************************************************************

peptide 66

number of base peptides = 2

number of missed cleavages = 0

number of optional links = 1

number of known links = 0

number of optional modifications = 0

number of known modifications = 0

calculated mass = 1274.6267

...............................................................................

isomer 1 / peptide 66

oooooooooooooooo

2A338--2K342 2E721--2K724

. . . . . . . . . . . . . . . . . . . . . . . . . . . . . . . . . . . . . . . .

psd analysis for psd peak list "7" (selected parent mass = 1273.996)

concise lists of psd assignments

simple chain fragmentation

chain 2 2 2 2

----- - - - -

3 3 7 7

3 4 2 2

8 2 1 4

rule A T F G K E H I K score

a 0.0

b 0.0

b' 0.0

b" 0.0

b-NH3 0.0

b-H2O 0.0

b(n-1)+H2O 0.0

y 2 2.0

y' 0.0

y" 0.0

y-NH3 0.0

y-H2O 4 2 1 8.0

linker fragmentation

no assignments of linker fragments

detailed list of psd assignments

number of unassigned psd peaks 23

number of psd peaks assigned 4

number of psd fragments assigned 4

number of psd assignments 4

total score 10.0

m(exp) m(calc) d[Da] score psd rule fragmentation site(s)

-------------------------------------------------------------------------------

877.800 877.431 0.4 4.0 y-H2O1 2I723-[2K724]

991.533 990.515 1.0 2.0 y-H2O2 2H722-[2I723]

1009.867 1008.525 1.3 2.0 y2 2H722-[2I723]

1186.733 1185.579 1.2 2.0 y-H2O4 2A338-[2T339]

*** legend ********************************************************************

peptide 67

number of base peptides = 2

number of missed cleavages = 0

number of optional links = 1

number of known links = 0

number of optional modifications = 0

number of known modifications = 0

calculated mass = 1274.6267

...............................................................................

isomer 1 / peptide 67

oooooooooooooooo

1A338--1K342 2E721--2K724

. . . . . . . . . . . . . . . . . . . . . . . . . . . . . . . . . . . . . . . .

psd analysis for psd peak list "7" (selected parent mass = 1273.996)

concise lists of psd assignments

simple chain fragmentation

chain 1 1 2 2

----- - - - -

3 3 7 7

3 4 2 2

8 2 1 4

rule A T F G K E H I K score

a 0.0

b 0.0

b' 0.0

b" 0.0

b-NH3 0.0

b-H2O 0.0

b(n-1)+H2O 0.0

y 2 2.0

y' 0.0

y" 0.0

y-NH3 0.0

y-H2O 4 2 1 8.0

linker fragmentation

no assignments of linker fragments

detailed list of psd assignments

number of unassigned psd peaks 23

number of psd peaks assigned 4

number of psd fragments assigned 4

number of psd assignments 4

total score 10.0

m(exp) m(calc) d[Da] score psd rule fragmentation site(s)

-------------------------------------------------------------------------------

877.800 877.431 0.4 4.0 y-H2O1 2I723-[2K724]

991.533 990.515 1.0 2.0 y-H2O2 2H722-[2I723]

1009.867 1008.525 1.3 2.0 y2 2H722-[2I723]

1186.733 1185.579 1.2 2.0 y-H2O4 1A338-[1T339]

*** legend ********************************************************************

peptide 68

number of base peptides = 3

number of missed cleavages = 1

number of optional links = 1

number of known links = 0

number of optional modifications = 0

number of known modifications = 0

calculated mass = 1541.8326

...............................................................................

isomer 1 / peptide 68

ooooooooooooooooooooooooooooooo

1V655--1K658 1L718--1R720 ~ 1E721--1K724

. . . . . . . . . . . . . . . . . . . . . . . . . . . . . . . . . . . . . . . .

psd analysis for psd peak list "2" (selected parent mass = 1542.509)

concise lists of psd assignments

simple chain fragmentation

chain 1 1 1 1

----- - - - -

6 6 7 7

5 5 1 2

5 8 8 4

rule V F A K L G R E H I K score

a 0.0

b 0.0

b' 0.0

b" 0.0

b-NH3 0.0

b-H2O 0.0

b(n-1)+H2O 0.0

y 3 6 2 6.0

y' 0.0

y" 0.0

y-NH3 3 6 5 8.0

y-H2O 0.0

linker fragmentation

no assignments of linker fragments

detailed list of psd assignments

number of unassigned psd peaks 41

number of psd peaks assigned 5

number of psd fragments assigned 6

number of psd assignments 6

total score 14.0

m(exp) m(calc) d[Da] score psd rule fragmentation site(s)

-------------------------------------------------------------------------------

951.333 949.525 1.8 2.0 y2 1H722-[1I723]

1355.533 1354.701 0.8 4.0 y-NH35 1G719-[1R720]

1411.400 1411.722 0.3 2.0 y-NH36 1L718-[1G719]

1427.600 1425.738 1.9 2.0 y-NH33 1V655-[1F656]

1427.600 1428.749 1.1 2.0 y6 1L718-[1G719]

1442.600 1442.764 0.2 2.0 y3 1V655-[1F656]

*** legend ********************************************************************

peptide 69

number of base peptides = 3

number of missed cleavages = 1

number of optional links = 1

number of known links = 0

number of optional modifications = 0

number of known modifications = 0

calculated mass = 1541.8326

...............................................................................

isomer 1 / peptide 69

oooooooooooooooo

1L718--1R720 ~ 1E721--1K724 2V655--2K658

. . . . . . . . . . . . . . . . . . . . . . . . . . . . . . . . . . . . . . . .

psd analysis for psd peak list "2" (selected parent mass = 1542.509)

concise lists of psd assignments

simple chain fragmentation

chain 1 1 2 2

----- - - - -

7 7 6 6

1 2 5 5

8 4 5 8

rule L G R E H I K V F A K score

a 0.0

b 0.0

b' 0.0

b" 0.0

b-NH3 0.0

b-H2O 0.0

b(n-1)+H2O 0.0

y 6 2 3 6.0

y' 0.0

y" 0.0

y-NH3 6 5 3 8.0

y-H2O 0.0

linker fragmentation

no assignments of linker fragments

detailed list of psd assignments

number of unassigned psd peaks 41

number of psd peaks assigned 5

number of psd fragments assigned 6

number of psd assignments 6

total score 14.0

m(exp) m(calc) d[Da] score psd rule fragmentation site(s)

-------------------------------------------------------------------------------

951.333 949.525 1.8 2.0 y2 1H722-[1I723]

1355.533 1354.701 0.8 4.0 y-NH35 1G719-[1R720]

1411.400 1411.722 0.3 2.0 y-NH36 1L718-[1G719]

1427.600 1425.738 1.9 2.0 y-NH33 2V655-[2F656]

1427.600 1428.749 1.1 2.0 y6 1L718-[1G719]

1442.600 1442.764 0.2 2.0 y3 2V655-[2F656]

*** legend ********************************************************************

peptide 70

number of base peptides = 3

number of missed cleavages = 1

number of optional links = 1

number of known links = 0

number of optional modifications = 0

number of known modifications = 0

calculated mass = 1295.6958

...............................................................................

isomer 1 / peptide 70

oooooooooooooooooooooooo

1E721--1K724 ~ 1R725 1Q866--1K868

. . . . . . . . . . . . . . . . . . . . . . . . . . . . . . . . . . . . . . . .

psd analysis for psd peak list "5" (selected parent mass = 1295.914)

concise lists of psd assignments

simple chain fragmentation

no assignments of simple chain fragments

linker fragmentation

link rule link score matching psd rule(s)

-------------------------------------------------------------------------------

egs 1K724-1K868 6.0 legs5,regs7,regs9,regs15

detailed list of psd assignments

number of unassigned psd peaks 19

number of psd peaks assigned 4

number of psd fragments assigned 4

number of psd assignments 4

total score 6.0

m(exp) m(calc) d[Da] score psd rule fragmentation site(s)

-------------------------------------------------------------------------------

428.600 429.259 0.7 1.5 regs7 1K724-[1K868]

470.067 470.261 0.2 1.5 regs9 1K724-[1K868]

571.500 573.301 1.8 1.5 regs15 1K724-[1K868]

683.467 682.400 1.1 1.5 legs5 [1K724]-1K868

*** legend ********************************************************************

peptide 71

number of base peptides = 3

number of missed cleavages = 1

number of optional links = 1

number of known links = 0

number of optional modifications = 0

number of known modifications = 0

calculated mass = 1295.6958

...............................................................................

isomer 1 / peptide 71

oooooooooooooooooooooooo

1E721--1K724 ~ 1R725 2Q866--2K868

. . . . . . . . . . . . . . . . . . . . . . . . . . . . . . . . . . . . . . . .

psd analysis for psd peak list "5" (selected parent mass = 1295.914)

concise lists of psd assignments

simple chain fragmentation

no assignments of simple chain fragments

linker fragmentation

link rule link score matching psd rule(s)

-------------------------------------------------------------------------------

egs 1K724-2K868 6.0 legs5,regs7,regs9,regs15

detailed list of psd assignments

number of unassigned psd peaks 19

number of psd peaks assigned 4

number of psd fragments assigned 4

number of psd assignments 4

total score 6.0

m(exp) m(calc) d[Da] score psd rule fragmentation site(s)

-------------------------------------------------------------------------------

428.600 429.259 0.7 1.5 regs7 1K724-[2K868]

470.067 470.261 0.2 1.5 regs9 1K724-[2K868]

571.500 573.301 1.8 1.5 regs15 1K724-[2K868]

683.467 682.400 1.1 1.5 legs5 [1K724]-2K868

*** legend ********************************************************************

peptide 72

number of base peptides = 2

number of missed cleavages = 0

number of optional links = 1

number of known links = 0

number of optional modifications = 0

number of known modifications = 0

calculated mass = 1274.6267

...............................................................................

isomer 1 / peptide 72

oooooooooooooooo

1E721--1K724 2A338--2K342

. . . . . . . . . . . . . . . . . . . . . . . . . . . . . . . . . . . . . . . .

psd analysis for psd peak list "7" (selected parent mass = 1273.996)

concise lists of psd assignments

simple chain fragmentation

chain 1 1 2 2

----- - - - -

7 7 3 3

2 2 3 4

1 4 8 2

rule E H I K A T F G K score

a 0.0

b 0.0

b' 0.0

b" 0.0

b-NH3 0.0

b-H2O 0.0

b(n-1)+H2O 0.0

y 2 2.0

y' 0.0

y" 0.0

y-NH3 0.0

y-H2O 2 1 4 8.0

linker fragmentation

no assignments of linker fragments

detailed list of psd assignments

number of unassigned psd peaks 23

number of psd peaks assigned 4

number of psd fragments assigned 4

number of psd assignments 4

total score 10.0

m(exp) m(calc) d[Da] score psd rule fragmentation site(s)

-------------------------------------------------------------------------------

877.800 877.431 0.4 4.0 y-H2O1 1I723-[1K724]

991.533 990.515 1.0 2.0 y-H2O2 1H722-[1I723]

1009.867 1008.525 1.3 2.0 y2 1H722-[1I723]

1186.733 1185.579 1.2 2.0 y-H2O4 2A338-[2T339]

*** legend ********************************************************************

peptide 73

number of base peptides = 2

number of missed cleavages = 0

number of optional links = 1

number of known links = 0

number of optional modifications = 0

number of known modifications = 0

calculated mass = 1274.6267

...............................................................................

isomer 1 / peptide 73

oooooooooooooooo

1A338--1K342 1E721--1K724

. . . . . . . . . . . . . . . . . . . . . . . . . . . . . . . . . . . . . . . .

psd analysis for psd peak list "7" (selected parent mass = 1273.996)

concise lists of psd assignments

simple chain fragmentation

chain 1 1 1 1

----- - - - -

3 3 7 7

3 4 2 2

8 2 1 4

rule A T F G K E H I K score

a 0.0

b 0.0

b' 0.0

b" 0.0

b-NH3 0.0

b-H2O 0.0

b(n-1)+H2O 0.0

y 2 2.0

y' 0.0

y" 0.0

y-NH3 0.0

y-H2O 4 2 1 8.0

linker fragmentation

no assignments of linker fragments

detailed list of psd assignments

number of unassigned psd peaks 23

number of psd peaks assigned 4

number of psd fragments assigned 4

number of psd assignments 4

total score 10.0

m(exp) m(calc) d[Da] score psd rule fragmentation site(s)

-------------------------------------------------------------------------------

877.800 877.431 0.4 4.0 y-H2O1 1I723-[1K724]

991.533 990.515 1.0 2.0 y-H2O2 1H722-[1I723]

1009.867 1008.525 1.3 2.0 y2 1H722-[1I723]

1186.733 1185.579 1.2 2.0 y-H2O4 1A338-[1T339]

*** legend ********************************************************************

peptide 74

number of base peptides = 2

number of missed cleavages = 1

number of optional links = 0

number of known links = 0

number of optional modifications = 0

number of known modifications = 0

calculated mass = 983.5929

...............................................................................

isomer 1 / peptide 74

1I1121--1K1124 ~ 1V1125--1K1128

. . . . . . . . . . . . . . . . . . . . . . . . . . . . . . . . . . . . . . . .

psd analysis for psd peak list "6" (selected parent mass = 983.462)

concise lists of psd assignments

simple chain fragmentation

chain 1 1

----- - -

1 1

1 1

2 2

1 8

rule I F T K V F T K score

a 0.0

b 6 2.0

b' 0.0

b" 0.0

b-NH3 0.0

b-H2O 6 2.0

b(n-1)+H2O 0.0

y 7 5 4.0

y' 0.0

y" 0.0

y-NH3 0.0

y-H2O 7 2.0

detailed list of psd assignments

number of unassigned psd peaks 19

number of psd peaks assigned 5

number of psd fragments assigned 5

number of psd assignments 5

total score 10.0

m(exp) m(calc) d[Da] score psd rule fragmentation site(s)

-------------------------------------------------------------------------------

621.000 622.393 1.4 2.0 y5 1T1123-[1K1124]

720.133 718.429 1.7 2.0 b-H2O6 [1F1126]-1T1127

738.267 736.440 1.8 2.0 b6 [1F1126]-1T1127

851.133 852.498 1.4 2.0 y-H2O7 1I1121-[1F1122]

869.000 870.509 1.5 2.0 y7 1I1121-[1F1122]

*** legend ********************************************************************

peptide 75

number of base peptides = 3

number of missed cleavages = 1

number of optional links = 1

number of known links = 0

number of optional modifications = 0

number of known modifications = 0

calculated mass = 1405.7689

...............................................................................

isomer 1 / peptide 75

oooooooooooooooooo

1Q474--1K475 1S1043--1K1046 ~ 1V1047--1R1050

. . . . . . . . . . . . . . . . . . . . . . . . . . . . . . . . . . . . . . . .

psd analysis for psd peak list "8" (selected parent mass = 1405.492)

concise lists of psd assignments

simple chain fragmentation

chain 1 1 1 1

----- - - - -

0 0 1 1

4 4 0 0

7 7 4 5

4 5 3 0

rule Q K S F G K V L V R score

a 0.0

b 0.0

b' 0.0

b" 0.0

b-NH3 0.0

b-H2O 0.0

b(n-1)+H2O 0.0

y 0.0

y' 0.0

y" 0.0

y-NH3 1 2.0

y-H2O 1 2.0

linker fragmentation

link rule link score matching psd rule(s)

-------------------------------------------------------------------------------

egs 1K475-1K1046 3.0 legs15,regs6

detailed list of psd assignments

number of unassigned psd peaks 16

number of psd peaks assigned 3

number of psd fragments assigned 4

number of psd assignments 4

total score 7.0

m(exp) m(calc) d[Da] score psd rule fragmentation site(s)

-------------------------------------------------------------------------------

461.000 460.217 0.8 1.5 legs15 [1K475]-1K1046

931.400 931.536 0.1 1.5 regs6 1K475-[1K1046]

1260.467 1259.700 0.8 2.0 y-H2O1 1Q474-[1K475]

1260.467 1260.684 0.2 2.0 y-NH31 1Q474-[1K475]

*** legend ********************************************************************

peptide 76

number of base peptides = 3

number of missed cleavages = 1

number of optional links = 1

number of known links = 0

number of optional modifications = 0

number of known modifications = 0

calculated mass = 1405.7689

...............................................................................

isomer 1 / peptide 76

ooooooooooooooooooooooooooooooooo

1S1043--1K1046 ~ 1V1047--1R1050 2Q474--2K475

. . . . . . . . . . . . . . . . . . . . . . . . . . . . . . . . . . . . . . . .

psd analysis for psd peak list "8" (selected parent mass = 1405.492)

concise lists of psd assignments

simple chain fragmentation

chain 1 1 2 2

----- - - - -

1 1 0 0

0 0 4 4

4 5 7 7

3 0 4 5

rule S F G K V L V R Q K score

a 0.0

b 0.0

b' 0.0

b" 0.0

b-NH3 0.0

b-H2O 0.0

b(n-1)+H2O 0.0

y 0.0

y' 0.0

y" 0.0

y-NH3 1 2.0

y-H2O 1 2.0

linker fragmentation

link rule link score matching psd rule(s)

-------------------------------------------------------------------------------

egs 1K1046-2K475 3.0 legs6,regs15

detailed list of psd assignments

number of unassigned psd peaks 16

number of psd peaks assigned 3

number of psd fragments assigned 4

number of psd assignments 4

total score 7.0

m(exp) m(calc) d[Da] score psd rule fragmentation site(s)

-------------------------------------------------------------------------------

461.000 460.217 0.8 1.5 regs15 1K1046-[2K475]

931.400 931.536 0.1 1.5 legs6 [1K1046]-2K475

1260.467 1259.700 0.8 2.0 y-H2O1 2Q474-[2K475]

1260.467 1260.684 0.2 2.0 y-NH31 2Q474-[2K475]

*** legend ********************************************************************

peptide 77

number of base peptides = 4

number of missed cleavages = 2

number of optional links = 1

number of known links = 0

number of optional modifications = 0

number of known modifications = 0

calculated mass = 1405.7802

...............................................................................

isomer 1 / peptide 77

oooooooooooooooooooooooo

1R654 ~ 1V655--1K658 1G726--1R729 ~ 1K730

. . . . . . . . . . . . . . . . . . . . . . . . . . . . . . . . . . . . . . . .

psd analysis for psd peak list "8" (selected parent mass = 1405.492)

concise lists of psd assignments

simple chain fragmentation

chain 1 1 1 1

----- - - - -

6 6 7 7

5 5 2 3

4 8 6 0

rule R V F A K G L S R K score

a 0.0

b 0.0

b' 0.0

b" 0.0

b-NH3 0.0

b-H2O 0.0

b(n-1)+H2O 0.0

y 1 2.0

y' 0.0

y" 0.0

y-NH3 0.0

y-H2O 0.0

linker fragmentation

link rule link score matching psd rule(s)

-------------------------------------------------------------------------------

egs 1K658-1K730 3.0 legs9,regs5

detailed list of psd assignments

number of unassigned psd peaks 16

number of psd peaks assigned 3

number of psd fragments assigned 3

number of psd assignments 3

total score 5.0

m(exp) m(calc) d[Da] score psd rule fragmentation site(s)

-------------------------------------------------------------------------------

559.400 560.352 1.0 1.5 regs5 1K658-[1K730]

703.333 702.394 0.9 1.5 legs9 [1K658]-1K730

931.400 932.505 1.1 2.0 y1 1A657-[1K658]

*** legend ********************************************************************

peptide 78

number of base peptides = 4

number of missed cleavages = 2

number of optional links = 1

number of known links = 0

number of optional modifications = 0

number of known modifications = 0

calculated mass = 1405.7802

...............................................................................

isomer 1 / peptide 78

oooooooooooooooooooooooo

1R654 ~ 1V655--1K658 2G726--2R729 ~ 2K730

. . . . . . . . . . . . . . . . . . . . . . . . . . . . . . . . . . . . . . . .

psd analysis for psd peak list "8" (selected parent mass = 1405.492)

concise lists of psd assignments

simple chain fragmentation

chain 1 1 2 2

----- - - - -

6 6 7 7

5 5 2 3

4 8 6 0

rule R V F A K G L S R K score

a 0.0

b 0.0

b' 0.0

b" 0.0

b-NH3 0.0

b-H2O 0.0

b(n-1)+H2O 0.0

y 1 2.0

y' 0.0

y" 0.0

y-NH3 0.0

y-H2O 0.0

linker fragmentation

link rule link score matching psd rule(s)

-------------------------------------------------------------------------------

egs 1K658-2K730 3.0 legs9,regs5

detailed list of psd assignments

number of unassigned psd peaks 16

number of psd peaks assigned 3

number of psd fragments assigned 3

number of psd assignments 3

total score 5.0

m(exp) m(calc) d[Da] score psd rule fragmentation site(s)

-------------------------------------------------------------------------------

559.400 560.352 1.0 1.5 regs5 1K658-[2K730]

703.333 702.394 0.9 1.5 legs9 [1K658]-2K730

931.400 932.505 1.1 2.0 y1 1A657-[1K658]

*** legend ********************************************************************

peptide 79

number of base peptides = 4

number of missed cleavages = 2

number of optional links = 1

number of known links = 0

number of optional modifications = 0

number of known modifications = 0

calculated mass = 1405.7802

...............................................................................

isomer 1 / peptide 79

oooooooooooooooooooooooooooooooo

1V655--1K658 1R725 ~ 1G726--1R729 ~ 1K730

. . . . . . . . . . . . . . . . . . . . . . . . . . . . . . . . . . . . . . . .

psd analysis for psd peak list "8" (selected parent mass = 1405.492)

concise lists of psd assignments

simple chain fragmentation

chain 1 1 1 1

----- - - - -

6 6 7 7

5 5 2 3

5 8 5 0

rule V F A K R G L S R K score

a 0.0

b 0.0

b' 0.0

b" 0.0

b-NH3 0.0

b-H2O 0.0

b(n-1)+H2O 0.0

y 1 2.0

y' 0.0

y" 0.0

y-NH3 3 4 4.0

y-H2O 3 4 4.0

linker fragmentation

link rule link score matching psd rule(s)

-------------------------------------------------------------------------------

egs 1K658-1K730 1.5 legs14

detailed list of psd assignments

number of unassigned psd peaks 15

number of psd peaks assigned 4

number of psd fragments assigned 6

number of psd assignments 6

total score 11.5

m(exp) m(calc) d[Da] score psd rule fragmentation site(s)

-------------------------------------------------------------------------------

634.200 634.309 0.1 1.5 legs14 [1K658]-1K730

1088.533 1088.606 0.1 2.0 y1 1A657-[1K658]

1175.467 1174.647 0.8 2.0 y-H2O4 1G726-[1L727]

1175.467 1175.631 0.2 2.0 y-NH34 1G726-[1L727]

1290.533 1288.701 1.8 2.0 y-H2O3 1V655-[1F656]

1290.533 1289.685 0.8 2.0 y-NH33 1V655-[1F656]

*** legend ********************************************************************

peptide 80

number of base peptides = 3

number of missed cleavages = 1

number of optional links = 1

number of known links = 0

number of optional modifications = 0

number of known modifications = 0

calculated mass = 1405.7577

...............................................................................

isomer 1 / peptide 80

oooooooooooooooo

1V655--1K658 2Q474--2K475 ~ 2E476--2K479

. . . . . . . . . . . . . . . . . . . . . . . . . . . . . . . . . . . . . . . .

psd analysis for psd peak list "8" (selected parent mass = 1405.492)

concise lists of psd assignments

simple chain fragmentation

chain 1 1 2 2

----- - - - -

6 6 4 4

5 5 7 7

5 8 4 9

rule V F A K Q K E A I K score

a 0.0

b 5 2.0

b' 0.0

b" 0.0

b-NH3 0.0

b-H2O 0.0

b(n-1)+H2O 0.0

y 1 4 4.0

y' 0.0

y" 0.0

y-NH3 0.0

y-H2O 0.0

linker fragmentation

link rule link score matching psd rule(s)

-------------------------------------------------------------------------------

egs 1K658-2K475 1.5 legs14

detailed list of psd assignments

number of unassigned psd peaks 15

number of psd peaks assigned 4

number of psd fragments assigned 4

number of psd assignments 4

total score 7.5

m(exp) m(calc) d[Da] score psd rule fragmentation site(s)

-------------------------------------------------------------------------------

461.000 460.277 0.7 2.0 y4 2K475-[2E476]

634.200 634.309 0.1 1.5 legs14 [1K658]-2K475

1088.533 1088.584 0.1 2.0 y1 1A657-[1K658]

1260.467 1259.652 0.8 2.0 b5 [2I478]-2K479

...............................................................................

isomer 2 / peptide 80

ooooooooooooooooooooooooooooooo

1V655--1K658 2Q474--2K475 ~ 2E476--2K479

. . . . . . . . . . . . . . . . . . . . . . . . . . . . . . . . . . . . . . . .

psd analysis for psd peak list "8" (selected parent mass = 1405.492)

concise lists of psd assignments

simple chain fragmentation

chain 1 1 2 2

----- - - - -

6 6 4 4

5 5 7 7

5 8 4 9

rule V F A K Q K E A I K score

a 0.0

b 0.0

b' 0.0

b" 0.0

b-NH3 0.0

b-H2O 0.0

b(n-1)+H2O 0.0

y 1 2.0

y' 0.0

y" 0.0

y-NH3 0.0

y-H2O 0.0

linker fragmentation

link rule link score matching psd rule(s)

-------------------------------------------------------------------------------

egs 1K658-2K479 1.5 legs14

detailed list of psd assignments

number of unassigned psd peaks 17

number of psd peaks assigned 2

number of psd fragments assigned 2

number of psd assignments 2

total score 3.5

m(exp) m(calc) d[Da] score psd rule fragmentation site(s)

-------------------------------------------------------------------------------

634.200 634.309 0.1 1.5 legs14 [1K658]-2K479

1088.533 1088.584 0.1 2.0 y1 1A657-[1K658]

*** legend ********************************************************************

peptide 81

number of base peptides = 4

number of missed cleavages = 2

number of optional links = 1

number of known links = 0

number of optional modifications = 0

number of known modifications = 0

calculated mass = 1405.7802

...............................................................................

isomer 1 / peptide 81

oooooooooooooooooooooooooooooooo

1V655--1K658 2R725 ~ 2G726--2R729 ~ 2K730

. . . . . . . . . . . . . . . . . . . . . . . . . . . . . . . . . . . . . . . .

psd analysis for psd peak list "8" (selected parent mass = 1405.492)

concise lists of psd assignments

simple chain fragmentation

chain 1 1 2 2

----- - - - -

6 6 7 7

5 5 2 3

5 8 5 0

rule V F A K R G L S R K score

a 0.0

b 0.0

b' 0.0

b" 0.0

b-NH3 0.0

b-H2O 0.0

b(n-1)+H2O 0.0

y 1 2.0

y' 0.0

y" 0.0

y-NH3 3 4 4.0

y-H2O 3 4 4.0

linker fragmentation

link rule link score matching psd rule(s)

-------------------------------------------------------------------------------

egs 1K658-2K730 1.5 legs14

detailed list of psd assignments

number of unassigned psd peaks 15

number of psd peaks assigned 4

number of psd fragments assigned 6

number of psd assignments 6

total score 11.5

m(exp) m(calc) d[Da] score psd rule fragmentation site(s)

-------------------------------------------------------------------------------

634.200 634.309 0.1 1.5 legs14 [1K658]-2K730

1088.533 1088.606 0.1 2.0 y1 1A657-[1K658]

1175.467 1174.647 0.8 2.0 y-H2O4 2G726-[2L727]

1175.467 1175.631 0.2 2.0 y-NH34 2G726-[2L727]

1290.533 1288.701 1.8 2.0 y-H2O3 1V655-[1F656]

1290.533 1289.685 0.8 2.0 y-NH33 1V655-[1F656]

*** legend ********************************************************************

peptide 82

number of base peptides = 4

number of missed cleavages = 2

number of optional links = 1

number of known links = 0

number of optional modifications = 0

number of known modifications = 0

calculated mass = 1405.7802

...............................................................................

isomer 1 / peptide 82

oooooooooooooooooooooooo

1G726--1R729 ~ 1K730 2R654 ~ 2V655--2K658

. . . . . . . . . . . . . . . . . . . . . . . . . . . . . . . . . . . . . . . .

psd analysis for psd peak list "8" (selected parent mass = 1405.492)

concise lists of psd assignments

simple chain fragmentation

chain 1 1 2 2

----- - - - -

7 7 6 6

2 3 5 5

6 0 4 8

rule G L S R K R V F A K score

a 0.0

b 0.0

b' 0.0

b" 0.0

b-NH3 0.0

b-H2O 0.0

b(n-1)+H2O 0.0

y 1 2.0

y' 0.0

y" 0.0

y-NH3 0.0

y-H2O 0.0

linker fragmentation

link rule link score matching psd rule(s)

-------------------------------------------------------------------------------

egs 1K730-2K658 3.0 legs5,regs9

detailed list of psd assignments

number of unassigned psd peaks 16

number of psd peaks assigned 3

number of psd fragments assigned 3

number of psd assignments 3

total score 5.0

m(exp) m(calc) d[Da] score psd rule fragmentation site(s)

-------------------------------------------------------------------------------

559.400 560.352 1.0 1.5 legs5 [1K730]-2K658

703.333 702.394 0.9 1.5 regs9 1K730-[2K658]

931.400 932.505 1.1 2.0 y1 2A657-[2K658]

*** legend ********************************************************************

peptide 83

number of base peptides = 4

number of missed cleavages = 2

number of optional links = 1

number of known links = 0

number of optional modifications = 0

number of known modifications = 0

calculated mass = 1405.7802

...............................................................................

isomer 1 / peptide 83

oooooooooooooooooooooooo

2R654 ~ 2V655--2K658 2G726--2R729 ~ 2K730

. . . . . . . . . . . . . . . . . . . . . . . . . . . . . . . . . . . . . . . .

psd analysis for psd peak list "8" (selected parent mass = 1405.492)

concise lists of psd assignments

simple chain fragmentation

chain 2 2 2 2

----- - - - -

6 6 7 7

5 5 2 3

4 8 6 0

rule R V F A K G L S R K score

a 0.0

b 0.0

b' 0.0

b" 0.0

b-NH3 0.0

b-H2O 0.0

b(n-1)+H2O 0.0

y 1 2.0

y' 0.0

y" 0.0

y-NH3 0.0

y-H2O 0.0

linker fragmentation

link rule link score matching psd rule(s)

-------------------------------------------------------------------------------

egs 2K658-2K730 3.0 legs9,regs5

detailed list of psd assignments

number of unassigned psd peaks 16

number of psd peaks assigned 3

number of psd fragments assigned 3

number of psd assignments 3

total score 5.0

m(exp) m(calc) d[Da] score psd rule fragmentation site(s)

-------------------------------------------------------------------------------

559.400 560.352 1.0 1.5 regs5 2K658-[2K730]

703.333 702.394 0.9 1.5 legs9 [2K658]-2K730

931.400 932.505 1.1 2.0 y1 2A657-[2K658]

*** legend ********************************************************************

peptide 84

number of base peptides = 4

number of missed cleavages = 2

number of optional links = 1

number of known links = 0

number of optional modifications = 0

number of known modifications = 0

calculated mass = 1405.7802

...............................................................................

isomer 1 / peptide 84

oooooooooooooooo

1R725 ~ 1G726--1R729 ~ 1K730 2V655--2K658

. . . . . . . . . . . . . . . . . . . . . . . . . . . . . . . . . . . . . . . .

psd analysis for psd peak list "8" (selected parent mass = 1405.492)

concise lists of psd assignments

simple chain fragmentation

chain 1 1 2 2

----- - - - -

7 7 6 6

2 3 5 5

5 0 5 8

rule R G L S R K V F A K score

a 0.0

b 0.0

b' 0.0

b" 0.0

b-NH3 0.0

b-H2O 0.0

b(n-1)+H2O 0.0

y 1 2.0

y' 0.0

y" 0.0

y-NH3 4 3 4.0

y-H2O 4 3 4.0

linker fragmentation

link rule link score matching psd rule(s)

-------------------------------------------------------------------------------

egs 1K730-2K658 1.5 regs14

detailed list of psd assignments

number of unassigned psd peaks 15

number of psd peaks assigned 4

number of psd fragments assigned 6

number of psd assignments 6

total score 11.5

m(exp) m(calc) d[Da] score psd rule fragmentation site(s)

-------------------------------------------------------------------------------

634.200 634.309 0.1 1.5 regs14 1K730-[2K658]

1088.533 1088.606 0.1 2.0 y1 2A657-[2K658]

1175.467 1174.647 0.8 2.0 y-H2O4 1G726-[1L727]

1175.467 1175.631 0.2 2.0 y-NH34 1G726-[1L727]

1290.533 1288.701 1.8 2.0 y-H2O3 2V655-[2F656]

1290.533 1289.685 0.8 2.0 y-NH33 2V655-[2F656]

*** legend ********************************************************************

peptide 85

number of base peptides = 3

number of missed cleavages = 1

number of optional links = 1

number of known links = 0

number of optional modifications = 0

number of known modifications = 0

calculated mass = 1405.7577

...............................................................................

isomer 1 / peptide 85

ooooooooooooooooooooooooooooooo

2Q474--2K475 ~ 2E476--2K479 2V655--2K658

. . . . . . . . . . . . . . . . . . . . . . . . . . . . . . . . . . . . . . . .

psd analysis for psd peak list "8" (selected parent mass = 1405.492)

concise lists of psd assignments

simple chain fragmentation

chain 2 2 2 2

----- - - - -

4 4 6 6

7 7 5 5

4 9 5 8

rule Q K E A I K V F A K score

a 0.0

b 5 2.0

b' 0.0

b" 0.0

b-NH3 0.0

b-H2O 0.0

b(n-1)+H2O 0.0

y 4 1 4.0

y' 0.0

y" 0.0

y-NH3 0.0

y-H2O 0.0

linker fragmentation

link rule link score matching psd rule(s)

-------------------------------------------------------------------------------

egs 2K475-2K658 1.5 regs14

detailed list of psd assignments

number of unassigned psd peaks 15

number of psd peaks assigned 4

number of psd fragments assigned 4

number of psd assignments 4

total score 7.5

m(exp) m(calc) d[Da] score psd rule fragmentation site(s)

-------------------------------------------------------------------------------

461.000 460.277 0.7 2.0 y4 2K475-[2E476]

634.200 634.309 0.1 1.5 regs14 2K475-[2K658]

1088.533 1088.584 0.1 2.0 y1 2A657-[2K658]

1260.467 1259.652 0.8 2.0 b5 [2I478]-2K479

...............................................................................

isomer 2 / peptide 85

oooooooooooooooo

2Q474--2K475 ~ 2E476--2K479 2V655--2K658

. . . . . . . . . . . . . . . . . . . . . . . . . . . . . . . . . . . . . . . .

psd analysis for psd peak list "8" (selected parent mass = 1405.492)

concise lists of psd assignments

simple chain fragmentation

chain 2 2 2 2

----- - - - -

4 4 6 6

7 7 5 5

4 9 5 8

rule Q K E A I K V F A K score

a 0.0

b 0.0

b' 0.0

b" 0.0

b-NH3 0.0

b-H2O 0.0

b(n-1)+H2O 0.0

y 1 2.0

y' 0.0

y" 0.0

y-NH3 0.0

y-H2O 0.0

linker fragmentation

link rule link score matching psd rule(s)

-------------------------------------------------------------------------------

egs 2K479-2K658 1.5 regs14

detailed list of psd assignments

number of unassigned psd peaks 17

number of psd peaks assigned 2

number of psd fragments assigned 2

number of psd assignments 2

total score 3.5

m(exp) m(calc) d[Da] score psd rule fragmentation site(s)

-------------------------------------------------------------------------------

634.200 634.309 0.1 1.5 regs14 2K479-[2K658]

1088.533 1088.584 0.1 2.0 y1 2A657-[2K658]

*** legend ********************************************************************

peptide 86

number of base peptides = 4

number of missed cleavages = 2

number of optional links = 1

number of known links = 0

number of optional modifications = 0

number of known modifications = 0

calculated mass = 1405.7802

...............................................................................

isomer 1 / peptide 86

oooooooooooooooooooooooooooooooo

2V655--2K658 2R725 ~ 2G726--2R729 ~ 2K730

. . . . . . . . . . . . . . . . . . . . . . . . . . . . . . . . . . . . . . . .

psd analysis for psd peak list "8" (selected parent mass = 1405.492)

concise lists of psd assignments

simple chain fragmentation

chain 2 2 2 2

----- - - - -

6 6 7 7

5 5 2 3

5 8 5 0

rule V F A K R G L S R K score

a 0.0

b 0.0

b' 0.0

b" 0.0

b-NH3 0.0

b-H2O 0.0

b(n-1)+H2O 0.0

y 1 2.0

y' 0.0

y" 0.0

y-NH3 3 4 4.0

y-H2O 3 4 4.0

linker fragmentation

link rule link score matching psd rule(s)

-------------------------------------------------------------------------------

egs 2K658-2K730 1.5 legs14

detailed list of psd assignments

number of unassigned psd peaks 15

number of psd peaks assigned 4

number of psd fragments assigned 6

number of psd assignments 6

total score 11.5

m(exp) m(calc) d[Da] score psd rule fragmentation site(s)

-------------------------------------------------------------------------------

634.200 634.309 0.1 1.5 legs14 [2K658]-2K730

1088.533 1088.606 0.1 2.0 y1 2A657-[2K658]

1175.467 1174.647 0.8 2.0 y-H2O4 2G726-[2L727]

1175.467 1175.631 0.2 2.0 y-NH34 2G726-[2L727]

1290.533 1288.701 1.8 2.0 y-H2O3 2V655-[2F656]

1290.533 1289.685 0.8 2.0 y-NH33 2V655-[2F656]

*** legend ********************************************************************

peptide 87

number of base peptides = 3

number of missed cleavages = 1

number of optional links = 1

number of known links = 0

number of optional modifications = 0

number of known modifications = 0

calculated mass = 1273.7002

...............................................................................

isomer 1 / peptide 87

oooooooooooooooooooooo

1A12--1K15 ~ 1R16 2E476--2K479

. . . . . . . . . . . . . . . . . . . . . . . . . . . . . . . . . . . . . . . .

psd analysis for psd peak list "7" (selected parent mass = 1273.996)

concise lists of psd assignments

simple chain fragmentation

chain 1 1 2 2

----- - - - -

0 0 4 4

1 1 7 7

2 6 6 9

rule A T I K R E A I K score

a 0.0

b 0.0

b' 0.0

b" 0.0

b-NH3 0.0

b-H2O 4 2.0

b(n-1)+H2O 0.0

y 0.0

y' 0.0

y" 0.0

y-NH3 4 2.0

y-H2O 0.0

linker fragmentation

link rule link score matching psd rule(s)

-------------------------------------------------------------------------------

egs 1K15-2K479 1.5 legs17

detailed list of psd assignments

number of unassigned psd peaks 24

number of psd peaks assigned 3

number of psd fragments assigned 3

number of psd assignments 3

total score 5.5

m(exp) m(calc) d[Da] score psd rule fragmentation site(s)

-------------------------------------------------------------------------------

815.667 814.431 1.2 1.5 legs17 [1K15]-2K479

1079.933 1081.578 1.6 2.0 b-H2O4 [1K15]-1R16

1186.733 1185.637 1.1 2.0 y-NH34 1A12-[1T13]

*** legend ********************************************************************

peptide 88

number of base peptides = 3

number of missed cleavages = 1

number of optional links = 1

number of known links = 0

number of optional modifications = 0

number of known modifications = 0

calculated mass = 1273.7002

...............................................................................

isomer 1 / peptide 88

oooooooooooooooooooooo

2A12--2K15 ~ 2R16 2E476--2K479

. . . . . . . . . . . . . . . . . . . . . . . . . . . . . . . . . . . . . . . .

psd analysis for psd peak list "7" (selected parent mass = 1273.996)

concise lists of psd assignments

simple chain fragmentation

chain 2 2 2 2

----- - - - -

0 0 4 4

1 1 7 7

2 6 6 9

rule A T I K R E A I K score

a 0.0

b 0.0

b' 0.0

b" 0.0

b-NH3 0.0

b-H2O 4 2.0

b(n-1)+H2O 0.0

y 0.0

y' 0.0

y" 0.0

y-NH3 4 2.0

y-H2O 0.0

linker fragmentation

link rule link score matching psd rule(s)

-------------------------------------------------------------------------------

egs 2K15-2K479 1.5 legs17

detailed list of psd assignments

number of unassigned psd peaks 24

number of psd peaks assigned 3

number of psd fragments assigned 3

number of psd assignments 3

total score 5.5

m(exp) m(calc) d[Da] score psd rule fragmentation site(s)

-------------------------------------------------------------------------------

815.667 814.431 1.2 1.5 legs17 [2K15]-2K479

1079.933 1081.578 1.6 2.0 b-H2O4 [2K15]-2R16

1186.733 1185.637 1.1 2.0 y-NH34 2A12-[2T13]

*** legend ********************************************************************

peptide 89

number of base peptides = 3

number of missed cleavages = 1

number of optional links = 1

number of known links = 0

number of optional modifications = 0

number of known modifications = 0

calculated mass = 1273.6638

...............................................................................

isomer 1 / peptide 89

ooooooooooooooooooooooooooooooooo

1K1229 ~ 1G1230--1R1233 2E476--2K479

. . . . . . . . . . . . . . . . . . . . . . . . . . . . . . . . . . . . . . . .

psd analysis for psd peak list "7" (selected parent mass = 1273.996)

concise lists of psd assignments

simple chain fragmentation

chain 1 1 2 2

----- - - - -

1 1 0 0

2 2 4 4

2 3 7 7

9 3 6 9

rule K G L D R E A I K score

a 0.0

b 1 2.0

b' 0.0

b" 0.0

b-NH3 0.0

b-H2O 0.0

b(n-1)+H2O 0.0

y 0.0

y' 0.0

y" 0.0

y-NH3 0.0

y-H2O 0.0

linker fragmentation

link rule link score matching psd rule(s)

-------------------------------------------------------------------------------

egs 1K1229-2K479 1.5 legs17

detailed list of psd assignments

number of unassigned psd peaks 26

number of psd peaks assigned 1

number of psd fragments assigned 2

number of psd assignments 2

total score 3.5

m(exp) m(calc) d[Da] score psd rule fragmentation site(s)

-------------------------------------------------------------------------------

815.667 814.395 1.3 1.5 legs17 [1K1229]-2K479

815.667 814.420 1.2 2.0 b1 [1K1229]-1G1230

*** legend ********************************************************************

peptide 90

number of base peptides = 3

number of missed cleavages = 1

number of optional links = 1

number of known links = 0

number of optional modifications = 0

number of known modifications = 0

calculated mass = 1273.6638

...............................................................................

isomer 1 / peptide 90

oooooooooo

2E476--2K479 2K1229 ~ 2G1230--2R1233

. . . . . . . . . . . . . . . . . . . . . . . . . . . . . . . . . . . . . . . .

psd analysis for psd peak list "7" (selected parent mass = 1273.996)

concise lists of psd assignments

simple chain fragmentation

chain 2 2 2 2

----- - - - -

0 0 1 1

4 4 2 2

7 7 2 3

6 9 9 3

rule E A I K K G L D R score

a 0.0

b 1 2.0

b' 0.0

b" 0.0

b-NH3 0.0

b-H2O 0.0

b(n-1)+H2O 0.0

y 0.0

y' 0.0

y" 0.0

y-NH3 0.0

y-H2O 0.0

linker fragmentation

link rule link score matching psd rule(s)

-------------------------------------------------------------------------------

egs 2K479-2K1229 1.5 regs17

detailed list of psd assignments

number of unassigned psd peaks 26

number of psd peaks assigned 1

number of psd fragments assigned 2

number of psd assignments 2

total score 3.5

m(exp) m(calc) d[Da] score psd rule fragmentation site(s)

-------------------------------------------------------------------------------

815.667 814.395 1.3 1.5 regs17 2K479-[2K1229]

815.667 814.420 1.2 2.0 b1 [2K1229]-2G1230

*** legend ********************************************************************

peptide 91

number of base peptides = 3

number of missed cleavages = 1

number of optional links = 1

number of known links = 0

number of optional modifications = 0

number of known modifications = 0

calculated mass = 1251.6220

...............................................................................

isomer 1 / peptide 91

oooooooooo

1S1043--1K1046 1K1229 ~ 1G1230--1R1233

. . . . . . . . . . . . . . . . . . . . . . . . . . . . . . . . . . . . . . . .

psd analysis for psd peak list "1" (selected parent mass = 1251.934)

concise lists of psd assignments

simple chain fragmentation

no assignments of simple chain fragments

linker fragmentation

link rule link score matching psd rule(s)

-------------------------------------------------------------------------------

egs 1K1046-1K1229 4.5 legs11,regs7,regs17

detailed list of psd assignments

number of unassigned psd peaks 12

number of psd peaks assigned 3

number of psd fragments assigned 3

number of psd assignments 3

total score 4.5

m(exp) m(calc) d[Da] score psd rule fragmentation site(s)

-------------------------------------------------------------------------------

551.000 551.259 0.3 1.5 legs11 [1K1046]-1K1229

628.177 629.350 1.2 1.5 regs7 1K1046-[1K1229]

815.586 814.395 1.2 1.5 regs17 1K1046-[1K1229]

*** legend ********************************************************************

peptide 92

number of base peptides = 3

number of missed cleavages = 1

number of optional links = 1

number of known links = 0

number of optional modifications = 0

number of known modifications = 0

calculated mass = 1251.6220

...............................................................................

isomer 1 / peptide 92

oooooooooo

1S1043--1K1046 2K1229 ~ 2G1230--2R1233

. . . . . . . . . . . . . . . . . . . . . . . . . . . . . . . . . . . . . . . .

psd analysis for psd peak list "1" (selected parent mass = 1251.934)

concise lists of psd assignments

simple chain fragmentation

no assignments of simple chain fragments

linker fragmentation

link rule link score matching psd rule(s)

-------------------------------------------------------------------------------

egs 1K1046-2K1229 4.5 legs11,regs7,regs17

detailed list of psd assignments

number of unassigned psd peaks 12

number of psd peaks assigned 3

number of psd fragments assigned 3

number of psd assignments 3

total score 4.5

m(exp) m(calc) d[Da] score psd rule fragmentation site(s)

-------------------------------------------------------------------------------

551.000 551.259 0.3 1.5 legs11 [1K1046]-2K1229

628.177 629.350 1.2 1.5 regs7 1K1046-[2K1229]

815.586 814.395 1.2 1.5 regs17 1K1046-[2K1229]

*** legend ********************************************************************

peptide 93

number of base peptides = 3

number of missed cleavages = 1

number of optional links = 1

number of known links = 0

number of optional modifications = 0

number of known modifications = 0

calculated mass = 1251.6583

...............................................................................

isomer 1 / peptide 93

oooooooooooooooooooooooo

1A12--1K15 ~ 1R16 1S1043--1K1046

. . . . . . . . . . . . . . . . . . . . . . . . . . . . . . . . . . . . . . . .

psd analysis for psd peak list "1" (selected parent mass = 1251.934)

concise lists of psd assignments

simple chain fragmentation

chain 1 1 1 1

----- - - - -

0 0 1 1

0 0 0 0

1 1 4 4

2 6 3 6

rule A T I K R S F G K score

a 0.0

b 0.0

b' 0.0

b" 0.0

b-NH3 0.0

b-H2O 0.0

b(n-1)+H2O 0.0

y 3 2.0

y' 0.0

y" 0.0

y-NH3 0.0

y-H2O 0.0

linker fragmentation

link rule link score matching psd rule(s)

-------------------------------------------------------------------------------

egs 1K15-1K1046 4.5 legs7,legs17,regs11

detailed list of psd assignments

number of unassigned psd peaks 11

number of psd peaks assigned 4

number of psd fragments assigned 4

number of psd assignments 4

total score 6.5

m(exp) m(calc) d[Da] score psd rule fragmentation site(s)

-------------------------------------------------------------------------------

551.000 551.259 0.3 1.5 regs11 1K15-[1K1046]

628.177 629.386 1.2 1.5 legs7 [1K15]-1K1046

815.586 814.431 1.2 1.5 legs17 [1K15]-1K1046

1080.000 1079.574 0.4 2.0 y3 1T13-[1I14]

*** legend ********************************************************************

peptide 94

number of base peptides = 3

number of missed cleavages = 1

number of optional links = 1

number of known links = 0

number of optional modifications = 0

number of known modifications = 0

calculated mass = 1251.6583

...............................................................................

isomer 1 / peptide 94

ooooooooooooooo

1S1043--1K1046 2A12--2K15 ~ 2R16

. . . . . . . . . . . . . . . . . . . . . . . . . . . . . . . . . . . . . . . .

psd analysis for psd peak list "1" (selected parent mass = 1251.934)

concise lists of psd assignments

simple chain fragmentation

chain 1 1 2 2

----- - - - -

1 1 0 0

0 0 0 0

4 4 1 1

3 6 2 6

rule S F G K A T I K R score

a 0.0

b 0.0

b' 0.0

b" 0.0

b-NH3 0.0

b-H2O 0.0

b(n-1)+H2O 0.0

y 3 2.0

y' 0.0

y" 0.0

y-NH3 0.0

y-H2O 0.0

linker fragmentation

link rule link score matching psd rule(s)

-------------------------------------------------------------------------------

egs 1K1046-2K15 4.5 legs11,regs7,regs17

detailed list of psd assignments

number of unassigned psd peaks 11

number of psd peaks assigned 4

number of psd fragments assigned 4

number of psd assignments 4

total score 6.5

m(exp) m(calc) d[Da] score psd rule fragmentation site(s)

-------------------------------------------------------------------------------

551.000 551.259 0.3 1.5 legs11 [1K1046]-2K15

628.177 629.386 1.2 1.5 regs7 1K1046-[2K15]

815.586 814.431 1.2 1.5 regs17 1K1046-[2K15]

1080.000 1079.574 0.4 2.0 y3 2T13-[2I14]

*** legend ********************************************************************

peptide 95

number of base peptides = 4

number of missed cleavages = 2

number of optional links = 1

number of known links = 0

number of optional modifications = 0

number of known modifications = 0

calculated mass = 1273.7114

...............................................................................

isomer 1 / peptide 95

oooooooooo

1G726--1R729 ~ 1K730 2K1350 ~ 2V1351--2K1353

. . . . . . . . . . . . . . . . . . . . . . . . . . . . . . . . . . . . . . . .

psd analysis for psd peak list "7" (selected parent mass = 1273.996)

concise lists of psd assignments

simple chain fragmentation

no assignments of simple chain fragments

linker fragmentation

link rule link score matching psd rule(s)

-------------------------------------------------------------------------------

egs 1K730-2K1350 3.0 legs15,regs14

detailed list of psd assignments

number of unassigned psd peaks 25

number of psd peaks assigned 2

number of psd fragments assigned 2

number of psd assignments 2

total score 3.0

m(exp) m(calc) d[Da] score psd rule fragmentation site(s)

-------------------------------------------------------------------------------

656.667 658.341 1.7 1.5 regs14 1K730-[2K1350]

744.333 745.397 1.1 1.5 legs15 [1K730]-2K1350

...............................................................................

isomer 2 / peptide 95

ooooooooooooooooooooooooooo

1G726--1R729 ~ 1K730 2K1350 ~ 2V1351--2K1353

. . . . . . . . . . . . . . . . . . . . . . . . . . . . . . . . . . . . . . . .

psd analysis for psd peak list "7" (selected parent mass = 1273.996)

concise lists of psd assignments

simple chain fragmentation

chain 1 1 2 2

----- - - - -

0 0 1 1

7 7 3 3

2 3 5 5

6 0 0 3

rule G L S R K K V N K score

a 0.0

b 0.0

b' 0.0

b" 0.0

b-NH3 0.0

b-H2O 0.0

b(n-1)+H2O 0.0

y 0.0

y' 0.0

y" 0.0

y-NH3 0.0

y-H2O 2 2.0

linker fragmentation

link rule link score matching psd rule(s)

-------------------------------------------------------------------------------

egs 1K730-2K1353 3.0 legs15,regs14

detailed list of psd assignments

number of unassigned psd peaks 24

number of psd peaks assigned 3

number of psd fragments assigned 3

number of psd assignments 3

total score 5.0

m(exp) m(calc) d[Da] score psd rule fragmentation site(s)

-------------------------------------------------------------------------------

656.667 658.341 1.7 1.5 regs14 1K730-[2K1353]

744.333 745.397 1.1 1.5 legs15 [1K730]-2K1353

1027.500 1028.538 1.0 2.0 y-H2O2 2V1351-[2N1352]

*** legend ********************************************************************

peptide 96

number of base peptides = 4

number of missed cleavages = 2

number of optional links = 1

number of known links = 0

number of optional modifications = 0

number of known modifications = 0

calculated mass = 1273.7114

...............................................................................

isomer 1 / peptide 96

oooooooooo

2G726--2R729 ~ 2K730 2K1350 ~ 2V1351--2K1353

. . . . . . . . . . . . . . . . . . . . . . . . . . . . . . . . . . . . . . . .

psd analysis for psd peak list "7" (selected parent mass = 1273.996)

concise lists of psd assignments

simple chain fragmentation

no assignments of simple chain fragments

linker fragmentation

link rule link score matching psd rule(s)

-------------------------------------------------------------------------------

egs 2K730-2K1350 3.0 legs15,regs14

detailed list of psd assignments

number of unassigned psd peaks 25

number of psd peaks assigned 2

number of psd fragments assigned 2

number of psd assignments 2

total score 3.0

m(exp) m(calc) d[Da] score psd rule fragmentation site(s)

-------------------------------------------------------------------------------

656.667 658.341 1.7 1.5 regs14 2K730-[2K1350]

744.333 745.397 1.1 1.5 legs15 [2K730]-2K1350

...............................................................................

isomer 2 / peptide 96

ooooooooooooooooooooooooooo

2G726--2R729 ~ 2K730 2K1350 ~ 2V1351--2K1353

. . . . . . . . . . . . . . . . . . . . . . . . . . . . . . . . . . . . . . . .

psd analysis for psd peak list "7" (selected parent mass = 1273.996)

concise lists of psd assignments

simple chain fragmentation

chain 2 2 2 2

----- - - - -

0 0 1 1

7 7 3 3

2 3 5 5

6 0 0 3

rule G L S R K K V N K score

a 0.0

b 0.0

b' 0.0

b" 0.0

b-NH3 0.0

b-H2O 0.0

b(n-1)+H2O 0.0

y 0.0

y' 0.0

y" 0.0

y-NH3 0.0

y-H2O 2 2.0

linker fragmentation

link rule link score matching psd rule(s)

-------------------------------------------------------------------------------

egs 2K730-2K1353 3.0 legs15,regs14

detailed list of psd assignments

number of unassigned psd peaks 24

number of psd peaks assigned 3

number of psd fragments assigned 3

number of psd assignments 3

total score 5.0

m(exp) m(calc) d[Da] score psd rule fragmentation site(s)

-------------------------------------------------------------------------------

656.667 658.341 1.7 1.5 regs14 2K730-[2K1353]

744.333 745.397 1.1 1.5 legs15 [2K730]-2K1353

1027.500 1028.538 1.0 2.0 y-H2O2 2V1351-[2N1352]

*** legend ********************************************************************

peptide 97

number of base peptides = 2

number of missed cleavages = 0

number of optional links = 1

number of known links = 0

number of optional modifications = 0

number of known modifications = 0

calculated mass = 983.5089

...............................................................................

isomer 1 / peptide 97

oooooooooooooooo

1G914--1K916 2I964--2K966

. . . . . . . . . . . . . . . . . . . . . . . . . . . . . . . . . . . . . . . .

psd analysis for psd peak list "6" (selected parent mass = 983.462)

concise lists of psd assignments

simple chain fragmentation

chain 1 1 2 2

----- - - - -

9 9 9 9

1 1 6 6

4 6 4 6

rule G F K I F K score

a 0.0

b 0.0

b' 0.0

b" 0.0

b-NH3 0.0

b-H2O 0.0

b(n-1)+H2O 0.0

y 2 2.0

y' 0.0

y" 0.0

y-NH3 0.0

y-H2O 0.0

linker fragmentation

link rule link score matching psd rule(s)

-------------------------------------------------------------------------------

egs 1K916-2K966 3.0 legs16,regs7

detailed list of psd assignments

number of unassigned psd peaks 21

number of psd peaks assigned 3

number of psd fragments assigned 3

number of psd assignments 3

total score 5.0

m(exp) m(calc) d[Da] score psd rule fragmentation site(s)

-------------------------------------------------------------------------------

449.400 448.269 1.1 1.5 regs7 1K916-[2K966]

548.333 550.264 1.9 1.5 legs16 [1K916]-2K966

869.000 870.425 1.4 2.0 y2 2I964-[2F965]

*** legend ********************************************************************

peptide 98

number of base peptides = 2

number of missed cleavages = 0

number of optional links = 1

number of known links = 0

number of optional modifications = 0

number of known modifications = 0

calculated mass = 983.5089

...............................................................................

isomer 1 / peptide 98

oooooooooooooooo

2G914--2K916 2I964--2K966

. . . . . . . . . . . . . . . . . . . . . . . . . . . . . . . . . . . . . . . .

psd analysis for psd peak list "6" (selected parent mass = 983.462)

concise lists of psd assignments

simple chain fragmentation

chain 2 2 2 2

----- - - - -

9 9 9 9

1 1 6 6

4 6 4 6

rule G F K I F K score

a 0.0

b 0.0

b' 0.0

b" 0.0

b-NH3 0.0

b-H2O 0.0

b(n-1)+H2O 0.0

y 2 2.0

y' 0.0

y" 0.0

y-NH3 0.0

y-H2O 0.0

linker fragmentation

link rule link score matching psd rule(s)

-------------------------------------------------------------------------------

egs 2K916-2K966 3.0 legs16,regs7

detailed list of psd assignments

number of unassigned psd peaks 21

number of psd peaks assigned 3

number of psd fragments assigned 3

number of psd assignments 3

total score 5.0

m(exp) m(calc) d[Da] score psd rule fragmentation site(s)

-------------------------------------------------------------------------------

449.400 448.269 1.1 1.5 regs7 2K916-[2K966]

548.333 550.264 1.9 1.5 legs16 [2K916]-2K966

869.000 870.425 1.4 2.0 y2 2I964-[2F965]

*** legend ********************************************************************

peptide 99

number of base peptides = 2

number of missed cleavages = 0

number of optional links = 1

number of known links = 0

number of optional modifications = 0

number of known modifications = 0

calculated mass = 983.5089

...............................................................................

isomer 1 / peptide 99

oooooooooooooooo

1G914--1K916 1I964--1K966

. . . . . . . . . . . . . . . . . . . . . . . . . . . . . . . . . . . . . . . .

psd analysis for psd peak list "6" (selected parent mass = 983.462)

concise lists of psd assignments

simple chain fragmentation

chain 1 1 1 1

----- - - - -

9 9 9 9

1 1 6 6

4 6 4 6

rule G F K I F K score

a 0.0

b 0.0

b' 0.0

b" 0.0

b-NH3 0.0

b-H2O 0.0

b(n-1)+H2O 0.0

y 2 2.0

y' 0.0

y" 0.0

y-NH3 0.0

y-H2O 0.0

linker fragmentation

link rule link score matching psd rule(s)

-------------------------------------------------------------------------------

egs 1K916-1K966 3.0 legs16,regs7

detailed list of psd assignments

number of unassigned psd peaks 21

number of psd peaks assigned 3

number of psd fragments assigned 3

number of psd assignments 3

total score 5.0

m(exp) m(calc) d[Da] score psd rule fragmentation site(s)

-------------------------------------------------------------------------------

449.400 448.269 1.1 1.5 regs7 1K916-[1K966]

548.333 550.264 1.9 1.5 legs16 [1K916]-1K966

869.000 870.425 1.4 2.0 y2 1I964-[1F965]

*** legend ********************************************************************

peptide 100

number of base peptides = 2

number of missed cleavages = 0

number of optional links = 1

number of known links = 0

number of optional modifications = 0

number of known modifications = 0

calculated mass = 983.5089

...............................................................................

isomer 1 / peptide 100

oooooooooooooooo

1I964--1K966 2G914--2K916

. . . . . . . . . . . . . . . . . . . . . . . . . . . . . . . . . . . . . . . .

psd analysis for psd peak list "6" (selected parent mass = 983.462)

concise lists of psd assignments

simple chain fragmentation

chain 1 1 2 2

----- - - - -

9 9 9 9

6 6 1 1

4 6 4 6

rule I F K G F K score

a 0.0

b 0.0

b' 0.0

b" 0.0

b-NH3 0.0

b-H2O 0.0

b(n-1)+H2O 0.0

y 2 2.0

y' 0.0

y" 0.0

y-NH3 0.0

y-H2O 0.0

linker fragmentation

link rule link score matching psd rule(s)

-------------------------------------------------------------------------------

egs 1K966-2K916 3.0 legs7,regs16

detailed list of psd assignments

number of unassigned psd peaks 21

number of psd peaks assigned 3

number of psd fragments assigned 3

number of psd assignments 3

total score 5.0

m(exp) m(calc) d[Da] score psd rule fragmentation site(s)

-------------------------------------------------------------------------------

449.400 448.269 1.1 1.5 legs7 [1K966]-2K916

548.333 550.264 1.9 1.5 regs16 1K966-[2K916]

869.000 870.425 1.4 2.0 y2 1I964-[1F965]

*** legend ********************************************************************

peptide 101

number of base peptides = 2

number of missed cleavages = 0

number of optional links = 1

number of known links = 0

number of optional modifications = 0

number of known modifications = 0

calculated mass = 927.4463

...............................................................................

isomer 1 / peptide 101

oooooooooooooooo

1G914--1K916 2G914--2K916

. . . . . . . . . . . . . . . . . . . . . . . . . . . . . . . . . . . . . . . .

psd analysis for psd peak list "3" (selected parent mass = 927.400)

concise lists of psd assignments

simple chain fragmentation

chain 1 1 2 2

----- - - - -

9 9 9 9

1 1 1 1

4 6 4 6

rule G F K G F K score

a 0.0

b 0.0

b' 0.0

b" 0.0

b-NH3 0.0

b-H2O 0.0

b(n-1)+H2O 0.0

y 2 1 2 1 12.0

y' 0.0

y" 0.0

y-NH3 0.0

y-H2O 0.0

linker fragmentation

link rule link score matching psd rule(s)

-------------------------------------------------------------------------------

egs 1K916-2K916 9.0 legs5,legs16,legs17,regs5,regs16,regs17

detailed list of psd assignments

number of unassigned psd peaks 22

number of psd peaks assigned 5

number of psd fragments assigned 10

number of psd assignments 10

total score 21.0

m(exp) m(calc) d[Da] score psd rule fragmentation site(s)

-------------------------------------------------------------------------------

351.500 351.203 0.3 1.5 legs5 [1K916]-2K916

351.500 351.203 0.3 1.5 regs5 1K916-[2K916]

550.500 550.264 0.2 1.5 legs16 [1K916]-2K916

550.500 550.264 0.2 1.5 regs16 1K916-[2K916]

578.000 577.251 0.7 1.5 legs17 [1K916]-2K916

578.000 577.251 0.7 1.5 regs17 1K916-[2K916]

723.333 723.356 0.0 4.0 y1 1F915-[1K916]

723.333 723.356 0.0 4.0 y1 2F915-[2K916]

870.000 870.425 0.4 2.0 y2 1G914-[1F915]

870.000 870.425 0.4 2.0 y2 2G914-[2F915]

*** legend ********************************************************************

psd analysis (summary)

**********************

legend

iid isomer id

pid peptide id

d deviation selected psd parent mass - exp. parent peak mass

selected psd parent mass = 927.400

psd peak list label = 3

exp.

assigned parent

rank iid/pid score peaks peak mass d[Da]

-------------------------------------------------------

1 1/101 21.0 5/27 927.400 0.0

...............................................................................

selected psd parent mass = 983.462

psd peak list label = 6

exp.

assigned parent

rank iid/pid score peaks peak mass d[Da]

-------------------------------------------------------

1 1/74 10.0 5/24 983.462 0.0

2 1/100 5.0 3/24 983.462 0.0

3 1/99 5.0 3/24 983.462 0.0

4 1/98 5.0 3/24 983.462 0.0

5 1/97 5.0 3/24 983.462 0.0

...............................................................................

selected psd parent mass = 1251.934

psd peak list label = 1

exp.

assigned parent

rank iid/pid score peaks peak mass d[Da]

-------------------------------------------------------

1 1/47 11.5 6/15 1251.934 0.0

2 1/46 11.5 6/15 1251.934 0.0

3 1/94 6.5 4/15 1251.934 0.0

4 1/93 6.5 4/15 1251.934 0.0

5 1/92 4.5 3/15 1251.934 0.0

6 1/91 4.5 3/15 1251.934 0.0

...............................................................................

selected psd parent mass = 1273.996

psd peak list label = 7

exp.

assigned parent

rank iid/pid score peaks peak mass d[Da]

-------------------------------------------------------

1 1/1 16.0 6/27 1273.996 0.0

2 1/73 10.0 4/27 1273.996 0.0

3 1/72 10.0 4/27 1273.996 0.0

4 1/67 10.0 4/27 1273.996 0.0

5 1/66 10.0 4/27 1273.996 0.0

6 1/50 8.5 5/27 1273.996 0.0

7 1/88 5.5 3/27 1273.996 0.0

8 1/87 5.5 3/27 1273.996 0.0

9 2/96 5.0 3/27 1273.996 0.0

10 2/95 5.0 3/27 1273.996 0.0

11 1/3 4.0 2/27 1273.996 0.0

12 1/2 4.0 2/27 1273.996 0.0

13 1/90 3.5 1/27 1273.996 0.0

14 1/89 3.5 1/27 1273.996 0.0

15 1/96 3.0 2/27 1273.996 0.0

16 1/95 3.0 2/27 1273.996 0.0

17 2/59 3.0 2/27 1273.996 0.0

18 1/59 3.0 2/27 1273.996 0.0

19 1/44 1.5 1/27 1273.996 0.0

20 1/42 1.5 1/27 1273.996 0.0

...............................................................................

selected psd parent mass = 1295.914

psd peak list label = 5

exp.

assigned parent

rank iid/pid score peaks peak mass d[Da]

-------------------------------------------------------

1 1/71 6.0 4/23 1295.914 0.0

2 1/70 6.0 4/23 1295.914 0.0

3 1/63 6.0 4/23 1295.914 0.0

4 1/62 6.0 4/23 1295.914 0.0

5 1/6 6.0 4/23 1295.914 0.0

6 1/49 1.5 1/23 1295.914 0.0

7 1/48 1.5 1/23 1295.914 0.0

...............................................................................

selected psd parent mass = 1405.492

psd peak list label = 8

exp.

assigned parent

rank iid/pid score peaks peak mass d[Da]

-------------------------------------------------------

1 1/86 11.5 4/19 1405.492 0.0

2 1/84 11.5 4/19 1405.492 0.0

3 1/81 11.5 4/19 1405.492 0.0

4 1/79 11.5 4/19 1405.492 0.0

5 1/51 9.0 4/19 1405.492 0.0

6 1/85 7.5 4/19 1405.492 0.0

7 1/80 7.5 4/19 1405.492 0.0

8 1/45 7.5 3/19 1405.492 0.0

9 1/43 7.5 3/19 1405.492 0.0

10 1/41 7.5 3/19 1405.492 0.0

11 1/40 7.5 3/19 1405.492 0.0

12 1/76 7.0 3/19 1405.492 0.0

13 1/75 7.0 3/19 1405.492 0.0

14 1/60 7.0 4/19 1405.492 0.0

15 1/58 7.0 4/19 1405.492 0.0

16 1/83 5.0 3/19 1405.492 0.0

17 1/82 5.0 3/19 1405.492 0.0

18 1/78 5.0 3/19 1405.492 0.0

19 1/77 5.0 3/19 1405.492 0.0

20 2/85 3.5 2/19 1405.492 0.0

21 2/80 3.5 2/19 1405.492 0.0

22 1/27 3.5 2/19 1405.492 0.0

23 2/27 1.5 1/19 1405.492 0.0

...............................................................................

selected psd parent mass = 1418.573

psd peak list label = 4

exp.

assigned parent

rank iid/pid score peaks peak mass d[Da]

-------------------------------------------------------

1 1/54 130.5 16/41 1418.573 0.0

2 1/53 130.5 16/41 1418.573 0.0

3 1/55 70.5 12/41 1418.573 0.0

4 1/52 70.5 12/41 1418.573 0.0

5 1/57 45.0 9/41 1418.573 0.0

6 1/56 45.0 9/41 1418.573 0.0

7 2/61 9.0 5/41 1418.573 0.0

8 1/61 9.0 5/41 1418.573 0.0

...............................................................................

selected psd parent mass = 1542.509

psd peak list label = 2

exp.

assigned parent

rank iid/pid score peaks peak mass d[Da]

-------------------------------------------------------

1 1/29 153.0 14/46 1542.509 0.0

2 1/28 153.0 14/46 1542.509 0.0

3 1/5 14.5 7/46 1542.509 0.0

4 1/4 14.5 7/46 1542.509 0.0

5 1/69 14.0 5/46 1542.509 0.0

6 1/68 14.0 5/46 1542.509 0.0

7 1/65 14.0 5/46 1542.509 0.0

8 1/64 14.0 5/46 1542.509 0.0

9 1/36 11.5 6/46 1542.509 0.0

10 1/35 11.5 6/46 1542.509 0.0

11 1/33 11.0 6/46 1542.509 0.0

12 1/32 11.0 6/46 1542.509 0.0

13 1/31 11.0 6/46 1542.509 0.0

14 1/30 11.0 6/46 1542.509 0.0

15 1/26 10.0 6/46 1542.509 0.0

16 1/25 10.0 6/46 1542.509 0.0

17 1/24 10.0 6/46 1542.509 0.0

18 1/23 10.0 6/46 1542.509 0.0

19 1/22 10.0 6/46 1542.509 0.0

20 1/21 10.0 6/46 1542.509 0.0

21 1/20 10.0 6/46 1542.509 0.0

22 1/19 10.0 6/46 1542.509 0.0

23 1/18 10.0 6/46 1542.509 0.0

24 1/17 10.0 6/46 1542.509 0.0

25 1/16 10.0 6/46 1542.509 0.0

26 1/15 10.0 6/46 1542.509 0.0

27 1/14 10.0 6/46 1542.509 0.0

28 1/13 10.0 6/46 1542.509 0.0

29 1/12 10.0 6/46 1542.509 0.0

30 1/11 10.0 6/46 1542.509 0.0

31 1/10 10.0 6/46 1542.509 0.0

32 1/9 10.0 6/46 1542.509 0.0

33 1/8 10.0 6/46 1542.509 0.0

34 1/7 10.0 6/46 1542.509 0.0

35 1/37 6.0 3/46 1542.509 0.0

36 1/34 4.5 3/46 1542.509 0.0

37 1/39 4.0 2/46 1542.509 0.0

38 1/38 4.0 2/46 1542.509 0.0

protein coverage

****************

legend

* covered residue (occurs in at least one peptide)

number of covered residues: 319

total number of residues: 2826

coverage [%]: 11

...............................................................................

chain 1 (chain 1 of protein 1)

number of covered residues: 186

total number of residues: 1413

coverage [%]: 13

10 20 30 40 50

AEISG ILCSD KATIK RTWAT VTDLP SFGRN VFLSV FAAKP EYKNL FVEFR

**** *

60 70 80 90 100

NIPAS ELASS ERLLY HGGRV LSSID EAIAG IDTPD RAVKT LLALG ERHIS

***** ***** ** **** ***** **

110 120 130 140 150

RGTVR RHFEA FSYAF IDELK QRGVE SADLA AWRRG WDNIV NVLEA GLLRR

160 170 180 190 200

QIDLE VTGLS CVDVA NIQES WSKVS GDLKT TGSVV FQRMI NGHPE YQQLF

210 220 230 240 250

RQFRD VDLDK LGESN SFVAH VFRVV AAFDG IIHEL DNNQF IVSTL KKLGE

****

260 270 280 290 300

QHIAR GTDIS HFQNF RVTLL EYLKE NGMNG AQKAS WNKAF DAFEK YISMG

***** ** ***

310 320 330 340 350

LSSLK RVDPI TGLSG LEKNA ILSTW GKVRG NLQEV GKATF GKLFT AHPEY

* ***** ***** **

360 370 380 390 400

QQMFR FSQGM PLASL VESPK FAAHT QRVVS ALDQT LLALN RPSDF VYMIK

410 420 430 440 450

ELGLD HINRG TDRSH FENYQ VVFIE YLKET LGDSL DEFTV KSFNH VFEVI

460 470 480 490 500

ISFLN EGLRQ ADIVD PVTHL TGRQK EMIKA SWSKA RTDLR SLGQE LFMRM

** * ***** * *

510 520 530 540 550

FKAHP EYQTL FVNKG FADVP LVSLR EDERF ISHMA NVLGG FDTLL QNLDE

**

560 570 580 590 600

SSYFI YSLRN LGDAH IQRKA GTQHF RSFEA ILIPY LQESQ GLDAA SVEAW

*

610 620 630 640 650

KKFFD VSIGV IAQGL KVATS EEADP VTGLY GKEIV ALRQA FAAVT PRNVE

* ***

660 670 680 690 700

IGKRV FAKLF AAHPE YKNLF KKFEQ YSVEE LPSTD AFHYH ISLVM NRFSS

***** *** *** ** ***

710 720 730 740 750

IGKVI DDNVS FVYLL KKLGR EHIKR GLSRK QFDQF VELYI AEISS ELSDT

*** **** ***** *****

760 770 780 790 800

GRNGL EKVLT FATGV IEQGL FQLGQ VDSNT LTALE KQSIQ DIWSN LRSTG

*** **

810 820 830 840 850

LQDLA VKIFT RLFSA HPEYK LLFTG RFGNV DNINE NAPFK AHLHR VLSAF

860 870 880 890 900

DIVIS TLDDS EHLIR QLKDL GLFHT RLGMT RSHFD NFATA FLSVA QDIAP

***

910 920 930 940 950

NQLTV LGRES LNKGF KLMHG VIEEG LLQLE RINPI TGLSA REVAV VKQTW

** *

960 970 980 990 1000

NLVKP DLMGV GMRIF KSLFE AFPAY QAVFP KFSDV PLDKL EDTPA VGKHA

** * * ***** *****

1010 1020 1030 1040 1050

ISVTT KLDEL IQTLD EPANL ALLAR QLGED HIVLR VNKPM FKSFG KVLVR

***** * ***** ***** *****

1060 1070 1080 1090 1100

LLEND LGQRF SSFAS RSWHK AYDVI VEYIE EGLQQ SYKQD PVTGI TDAEK

**** ** ***** *****

1110 1120 1130 1140 1150

ALVQE SWDLL KPDLL GLGRK IFTKV FTKHP DYQIL FTRTG FGDTP LTKLD

* ***** ***

1160 1170 1180 1190 1200

DNPAF GTHII KVMRA FDHVI QILGK PKTLM AYLRS VGADH IARNV ERRHF

1210 1220 1230 1240 1250

QAFSN ALIPV MQHEL KAQLR PDAVA AWRKG LDRII GIIDQ GLIGL KEVNP

** ***

1260 1270 1280 1290 1300

QNAFS AYDIQ AVQRT WALAK PDLMG KGAMV FKQLF TDHGY QPLFS NLAQY

1310 1320 1330 1340 1350

EITGL EGSPE LNTHA RNVMA QLDTL VGSLQ NSIEL GQSLA QLGKD HVPRK

*

1360 1370 1380 1390 1400

VNRVH FKDFA EHFIP LMKAD LGDEF TPLAE SAWKK AFDVM IATIE QGQRA

*** *

1410

RRSVA TFLTN PVA

...............................................................................

chain 2 (chain 1 of protein 2)

number of covered residues: 133

total number of residues: 1413

coverage [%]: 9

10 20 30 40 50

AEVRG ILCSD KATIK RTWSI VNDLP SFGRN VFLSV FAAKP EYKNL FVEFR

**** *

60 70 80 90 100

NIPAS ELANS ERLLY HGGRV LASID EVISE IDSPD SAAKK LVALG ERHIT

* ***** **

110 120 130 140 150

RGTVR RHFEA FSYAF IDELK QRGVA SADLA AWRKG WDSIV DILEA GLLKR

*

160 170 180 190 200

QIDLE VTGLS CVDVA NVQES WATVS ANLKN TGSIL FQRLI NDHPE YQQLF

210 220 230 240 250

RQFRD VELAK LGESN GFVAH VFRVV AAFDG IIKEL DNNPF IVSTL KRLGE

260 270 280 290 300

QHIAR GTDIS HFQNF RTTLL VYLNE NGMNQ AQEAS WNKAF DAIEK YISIG

*****

310 320 330 340 350

LKSLG RVDPI TGLSG LEKNA ILNTW GKVRG NLQEV GKATF GKLFA AHPEY

** * ***** ***** **

360 370 380 390 400

QQMFR FFQGV QLAEL VDSPK FAAHT QRVVS ALDQT LLALN RPSDF VYMIK

410 420 430 440 450

ELGLD HINRG TDRSH FENYQ VVFVE YLKET LGDSV DEFTV KSFNH VFEVI

460 470 480 490 500

INFLN EGLRQ ANVVD PVTHL TGRQK EAIKA SWSVA RTDLR FLGQE LFMRM

** ****

510 520 530 540 550

FNLNP EYQSL FVNKG FADVP LVSLR EDERF ISHMA NVLRG FDTLL QNLDD

560 570 580 590 600

TSYFV YALRN LGDAH IQRKA GTEHF RSFEA ILIPY LQESQ GLDAA GVEAW

*

610 620 630 640 650

KIFFD VSIGV IAQGL KVASS EEADP VTGLY GKEVV ALRQA FAAIS PRNVE

***

660 670 680 690 700

IGKRV FAKLF TSHPE YKNLF KKFEQ YSVEE LPSTD AFDYH ISLVM NRFSA

***** *** *** **

710 720 730 740 750

VGKVI DDNVS FVYLL KKLGR EHIKR GLSRK QFDQF VELYI AEISP ELSET

**** ***** *****

760 770 780 790 800

GRSGL EKVLT FATGV IEQGL FQLGQ VDSKA LTALE KQSIQ DIWTS LRPTG

810 820 830 840 850

LEELA VKMFT RLFAD HPEYK LLFTG RLGNV DNINE NAPFR AHLHR VLSAF

860 870 880 890 900

DIVIT SLDNN ALLIR QLKDL GLFHT RLGMT RAHFD NFATA FFSVA EDIVP

***

910 920 930 940 950

NLLTA LGRES LGKGF KLMVA VIEEG LLQLE RIDPI TGLSV REVEV VKQTW

** *

960 970 980 990 1000

NLVKP DLMGV GMRIF KSLFE KFPAY QAVFP KFSDV PLDKL EDIPA VGKHA

** ***** * **

1010 1020 1030 1040 1050

ISVTT KLDEL IQTLD EPANL ALLAR QLGED HIVLG VNKPM FKSFG EVLVR

***** *

1060 1070 1080 1090 1100

LLEND LGQRF SNFAS KSWHR AYDVI VEYIE EGLQQ SYKQD PVTGI TDAEK

* ***** * ** ***** *****

1110 1120 1130 1140 1150

VLVQR SWELL KPDLL GLGRK IFGVI FTKHP EYQIL FTRVG FGDTP LTQLD

*

1160 1170 1180 1190 1200

NNPAF GEHII KVMRA FDYVI RNLGK PKTLL AYLKN VGADH IARNV ERRHF

1210 1220 1230 1240 1250

QAFSE ALIPV MQREL KAQLK PEAVA AWRKG LDRII GVIDQ GLLGL KEVNP

** * ** ***

1260 1270 1280 1290 1300

QIAFS AADIE AIQKT WALAK PDLMG KGASV FRQLF TDHGY QPLFS NLVEY

1310 1320 1330 1340 1350

EVTGL EGSPE LNTHA RNVMA QLDTL VGSLQ NSIEL GKSLN QLGKD HVPRK

*** **** *

1360 1370 1380 1390 1400

VNKVH FDDFA EHFVP LMKAN LGDEF TPLAE SAWKK AFNVM VATIE QGQRA

*** *

1410

RRSIA TFLTN PVA

peak list (sorted)

******************

isd isd matching

number of parent fragment selected

assigned peak peak psd parent

mass peptides (ipp) (ifp) mass(es)

-------------------------------------------------------------------------------

927.400 1 927.400

983.462 5 983.462

1251.934 6 1251.934

1273.996 17 1273.996

1295.914 7 1295.914

1405.492 20 1405.492

1418.573 7 1418.573

1542.509 38 1542.509

psd peak lists (sorted)

***********************

list label parent mass

-----------------------------

3 927.400

6 983.462

1 1251.934

7 1273.996

5 1295.914

8 1405.492

4 1418.573

2 1542.509

list label parent mass

-----------------------------

3 927.400

mass

----------

322.000

351.500

437.067

455.267

468.067

538.333

550.500

553.267

568.333

578.000

581.333

625.467

652.400

680.267

698.333

707.000

723.333

755.600

795.333

813.333

814.467

854.000

870.000

891.467

896.000

908.333

926.467

list label parent mass

-----------------------------

6 983.462

mass

----------

449.400

482.733

527.133

548.333

582.267

621.000

669.467

720.133

738.267

764.667

773.467

782.667

797.000

797.400

808.133

821.533

851.133

869.000

907.400

924.067

964.400

965.333

982.467

982.733

list label parent mass

-----------------------------

1 1251.934

mass

----------

214.840

243.000

376.103

436.000

508.000

516.277

551.000

595.179

628.177

815.586

902.500

992.283

1080.000

1170.844

1229.788

list label parent mass

-----------------------------

7 1273.996

mass

----------

453.400

464.500

479.500

507.467

593.200

633.467

656.667

683.733

744.333

815.667

877.800

903.600

965.000

991.533

1009.867

1027.500

1079.933

1124.500

1135.500

1157.000

1168.000

1170.000

1179.667

1186.733

1214.000

1229.800

1274.000

list label parent mass

-----------------------------

5 1295.914

mass

----------

357.733

359.733

375.267

425.500

428.600

470.067

542.733

550.667

571.500

582.400

626.667

665.067

683.467

727.533

818.667

857.533

903.400

1017.000

1049.000

1109.533

1137.000

1273.867

1277.200

list label parent mass

-----------------------------

8 1405.492

mass

----------

461.000

559.400

634.200

703.333

750.400

816.467

865.533

893.267

931.400

979.400

1088.533

1128.333

1175.467

1203.400

1260.467

1272.533

1290.533

1388.600

1406.000

list label parent mass

-----------------------------

4 1418.573

mass

----------

441.892

489.193

645.500

659.038

725.167

733.500

751.500

781.516

828.485

859.087

861.083

939.386

943.500

961.500

963.000

978.438

1008.509

1052.284

1056.000

1074.438

1100.000

1118.346

1119.667

1134.500

1186.338

1214.678

1230.000

1242.000

1247.500

1259.651

1286.661

1303.500

1330.503

1344.541

1347.500

1356.582

1357.380

1374.545

1401.621

1402.553

1418.320

list label parent mass

-----------------------------

2 1542.509

mass

----------

536.200

570.533

680.000

731.200

793.133

881.400

915.000

931.500

951.333

1016.267

1044.500

1105.533

1113.867

1167.000

1204.000

1230.000

1298.667

1315.533

1323.500

1328.167

1334.667

1339.500

1341.000

1355.533

1357.267

1385.200

1397.467

1403.500

1411.400

1422.500

1427.600

1442.600

1451.533

1468.600

1469.500

1480.467

1481.000

1489.000

1496.467

1498.533

1499.667

1507.500

1513.467

1524.467

1525.467

1542.600
