# Supplementary material for: Quaternary structure of Artemia haemoglobin II: analysis of T and C polymer alignment and interpolymer interface
Source: BMC Struct Biol. 2007 Apr 18;7:26. doi: 10.1186/1472-6807-7-26 (PMC1865544; doi:10.1186/1472-6807-7-26)
Supplement: Additional file 6 — Raw SeachXlinks scoring outputs corresponding to parameter file parameter_TPCP_gmbs.txt (Additional file 3). [file 1472-6807-7-26-S6.doc]

**GMBS MS3D raw SearchXlinks output**

This is the raw SearchXlinks output generated using the mass spectrometry data from the GMBS-crosslinking/trypsinolysis/RP-HPLC/ESI-MS experiment. A total of 123 significantly ionized species were identified from a total of 17 RP-HPLC fractions (rpf1 to rpf 17 as indicated on the parameter file). All of them were subjected to the ESI-MS zoom scan analysis to determine charge state. It was known that ions with masses less than 800 Da cannot be GMBS-crosslinked tryptic fragments of HbII at all, so ions with mass less than 800 Da were silently excluded, and thus only 37 significantly ionized species were subjected to SearchXlinks analysis. Only one peak (the 1381.305 peak of rpf8) was subjected to MS/MS because it is the only peak showing possible correlation to GMBS-crosslinked tryptic fragments of HbII. SearchXlinks scoring of the 1381.305 peak were included.

sxl version 3.1.5 checked in on Mon, 06 Dec 2004 12:50:00 +0100

!! INFO this is an html document to be viewed with a browser. if this document

!! INFO is loaded from disk into the browser, its filename must have the

!! INFO extension .html or .htm. to remove html tags, save the document as a

!! INFO text file from within your browser.

!! INFO this html document provides tooltips, i.e., moving the mouse pointer

!! INFO over a cross-linker or a modification of a peptide isomer outline will

!! INFO make your browser display the type of the cross-link or modification.

!! INFO however, this does not work for all browsers.

contents

********

[list of amino acid chains](http://www.searchxlinks.de/cgi-bin/upload_action.pl#lof_aa_chains%23lof_aa_chains)

[digest](http://www.searchxlinks.de/cgi-bin/upload_action.pl#digest%23digest)

[sorted list of assignments](http://www.searchxlinks.de/cgi-bin/upload_action.pl#sorted_list%23sorted_list)

[peptide isomers](http://www.searchxlinks.de/cgi-bin/upload_action.pl#peptide_isomers%23peptide_isomers)

[psd analysis](http://www.searchxlinks.de/cgi-bin/upload_action.pl#psd_analysis%23psd_analysis)

[protein coverage](http://www.searchxlinks.de/cgi-bin/upload_action.pl#protein_coverage%23protein_coverage)

[peak list](http://www.searchxlinks.de/cgi-bin/upload_action.pl#peak_list%23peak_list)

[list of amino acid chains](http://www.searchxlinks.de/cgi-bin/upload_action.pl#contents%23contents)

*************************

chain id protein

global local label fasta comment

-------------------------------------------------------------------------------

1 1 1 >TP

2 1 2 >CP

[digest](http://www.searchxlinks.de/cgi-bin/upload_action.pl#contents%23contents)

******

legend

| cleavage site after this residue

k cleavage site after this residue prevented by a known link/modification

cleavage rule: trypsin (K,R)-(*\P)

cleavage sites:

number of potential cleavages = 309

number of prevented cleavages = 0

...............................................................................

chain 1 (chain 1 of protein 1)

10 20 30 40 50

AEISG ILCSD KATIK RTWAT VTDLP SFGRN VFLSV FAAKP EYKNL FVEFR

| | | | | |

60 70 80 90 100

NIPAS ELASS ERLLY HGGRV LSSID EAIAG IDTPD RAVKT LLALG ERHIS

| | | | |

110 120 130 140 150

RGTVR RHFEA FSYAF IDELK QRGVE SADLA AWRRG WDNIV NVLEA GLLRR

| | | | | || ||

160 170 180 190 200

QIDLE VTGLS CVDVA NIQES WSKVS GDLKT TGSVV FQRMI NGHPE YQQLF

| | |

210 220 230 240 250

RQFRD VDLDK LGESN SFVAH VFRVV AAFDG IIHEL DNNQF IVSTL KKLGE

| | | | ||

260 270 280 290 300

QHIAR GTDIS HFQNF RVTLL EYLKE NGMNG AQKAS WNKAF DAFEK YISMG

| | | | | |

310 320 330 340 350

LSSLK RVDPI TGLSG LEKNA ILSTW GKVRG NLQEV GKATF GKLFT AHPEY

| | | | | | |

360 370 380 390 400

QQMFR FSQGM PLASL VESPK FAAHT QRVVS ALDQT LLALN RPSDF VYMIK

| | | |

410 420 430 440 450

ELGLD HINRG TDRSH FENYQ VVFIE YLKET LGDSL DEFTV KSFNH VFEVI

| | | |

460 470 480 490 500

ISFLN EGLRQ ADIVD PVTHL TGRQK EMIKA SWSKA RTDLR SLGQE LFMRM

| | | | | | | |

510 520 530 540 550

FKAHP EYQTL FVNKG FADVP LVSLR EDERF ISHMA NVLGG FDTLL QNLDE

| | | |

560 570 580 590 600

SSYFI YSLRN LGDAH IQRKA GTQHF RSFEA ILIPY LQESQ GLDAA SVEAW

| || |

610 620 630 640 650

KKFFD VSIGV IAQGL KVATS EEADP VTGLY GKEIV ALRQA FAAVT PRNVE

|| | | | |

660 670 680 690 700

IGKRV FAKLF AAHPE YKNLF KKFEQ YSVEE LPSTD AFHYH ISLVM NRFSS

|| | | || |

710 720 730 740 750

IGKVI DDNVS FVYLL KKLGR EHIKR GLSRK QFDQF VELYI AEISS ELSDT

| || | || ||

760 770 780 790 800

GRNGL EKVLT FATGV IEQGL FQLGQ VDSNT LTALE KQSIQ DIWSN LRSTG

| | | |

810 820 830 840 850

LQDLA VKIFT RLFSA HPEYK LLFTG RFGNV DNINE NAPFK AHLHR VLSAF

| | | | | |

860 870 880 890 900

DIVIS TLDDS EHLIR QLKDL GLFHT RLGMT RSHFD NFATA FLSVA QDIAP

| | | |

910 920 930 940 950

NQLTV LGRES LNKGF KLMHG VIEEG LLQLE RINPI TGLSA REVAV VKQTW

| | | | | |

960 970 980 990 1000

NLVKP DLMGV GMRIF KSLFE AFPAY QAVFP KFSDV PLDKL EDTPA VGKHA

| | | | |

1010 1020 1030 1040 1050

ISVTT KLDEL IQTLD EPANL ALLAR QLGED HIVLR VNKPM FKSFG KVLVR

| | | | | |

1060 1070 1080 1090 1100

LLEND LGQRF SSFAS RSWHK AYDVI VEYIE EGLQQ SYKQD PVTGI TDAEK

| | | | |

1110 1120 1130 1140 1150

ALVQE SWDLL KPDLL GLGRK IFTKV FTKHP DYQIL FTRTG FGDTP LTKLD

|| | | | |

1160 1170 1180 1190 1200

DNPAF GTHII KVMRA FDHVI QILGK PKTLM AYLRS VGADH IARNV ERRHF

| | | | | ||

1210 1220 1230 1240 1250

QAFSN ALIPV MQHEL KAQLR PDAVA AWRKG LDRII GIIDQ GLIGL KEVNP

| || | |

1260 1270 1280 1290 1300

QNAFS AYDIQ AVQRT WALAK PDLMG KGAMV FKQLF TDHGY QPLFS NLAQY

| | |

1310 1320 1330 1340 1350

EITGL EGSPE LNTHA RNVMA QLDTL VGSLQ NSIEL GQSLA QLGKD HVPRK

| | ||

1360 1370 1380 1390 1400

VNRVH FKDFA EHFIP LMKAD LGDEF TPLAE SAWKK AFDVM IATIE QGQRA

| | | || |

1410

RRSVA TFLTN PVA

||

...............................................................................

chain 2 (chain 1 of protein 2)

10 20 30 40 50

AEVRG ILCSD KATIK RTWSI VNDLP SFGRN VFLSV FAAKP EYKNL FVEFR

| | | | | | |

60 70 80 90 100

NIPAS ELANS ERLLY HGGRV LASID EVISE IDSPD SAAKK LVALG ERHIT

| | || |

110 120 130 140 150

RGTVR RHFEA FSYAF IDELK QRGVA SADLA AWRKG WDSIV DILEA GLLKR

| | | | | || ||

160 170 180 190 200

QIDLE VTGLS CVDVA NVQES WATVS ANLKN TGSIL FQRLI NDHPE YQQLF

| |

210 220 230 240 250

RQFRD VELAK LGESN GFVAH VFRVV AAFDG IIKEL DNNPF IVSTL KRLGE

| | | | | ||

260 270 280 290 300

QHIAR GTDIS HFQNF RTTLL VYLNE NGMNQ AQEAS WNKAF DAIEK YISIG

| | | |

310 320 330 340 350

LKSLG RVDPI TGLSG LEKNA ILNTW GKVRG NLQEV GKATF GKLFA AHPEY

| | | | | | |

360 370 380 390 400

QQMFR FFQGV QLAEL VDSPK FAAHT QRVVS ALDQT LLALN RPSDF VYMIK

| | | |

410 420 430 440 450

ELGLD HINRG TDRSH FENYQ VVFVE YLKET LGDSV DEFTV KSFNH VFEVI

| | | |

460 470 480 490 500

INFLN EGLRQ ANVVD PVTHL TGRQK EAIKA SWSVA RTDLR FLGQE LFMRM

| | | | | | |

510 520 530 540 550

FNLNP EYQSL FVNKG FADVP LVSLR EDERF ISHMA NVLRG FDTLL QNLDD

| | | |

560 570 580 590 600

TSYFV YALRN LGDAH IQRKA GTEHF RSFEA ILIPY LQESQ GLDAA GVEAW

| || |

610 620 630 640 650

KIFFD VSIGV IAQGL KVASS EEADP VTGLY GKEVV ALRQA FAAIS PRNVE

| | | | |

660 670 680 690 700

IGKRV FAKLF TSHPE YKNLF KKFEQ YSVEE LPSTD AFDYH ISLVM NRFSA

|| | | || |

710 720 730 740 750

VGKVI DDNVS FVYLL KKLGR EHIKR GLSRK QFDQF VELYI AEISP ELSET

| || | || ||

760 770 780 790 800

GRSGL EKVLT FATGV IEQGL FQLGQ VDSKA LTALE KQSIQ DIWTS LRPTG

| | | |

810 820 830 840 850

LEELA VKMFT RLFAD HPEYK LLFTG RLGNV DNINE NAPFR AHLHR VLSAF

| | | | | |

860 870 880 890 900

DIVIT SLDNN ALLIR QLKDL GLFHT RLGMT RAHFD NFATA FFSVA EDIVP

| | | |

910 920 930 940 950

NLLTA LGRES LGKGF KLMVA VIEEG LLQLE RIDPI TGLSV REVEV VKQTW

| | | | | |

960 970 980 990 1000

NLVKP DLMGV GMRIF KSLFE KFPAY QAVFP KFSDV PLDKL EDIPA VGKHA

| | | | | |

1010 1020 1030 1040 1050

ISVTT KLDEL IQTLD EPANL ALLAR QLGED HIVLG VNKPM FKSFG EVLVR

| | | |

1060 1070 1080 1090 1100

LLEND LGQRF SNFAS KSWHR AYDVI VEYIE EGLQQ SYKQD PVTGI TDAEK

| | | | |

1110 1120 1130 1140 1150

VLVQR SWELL KPDLL GLGRK IFGVI FTKHP EYQIL FTRVG FGDTP LTQLD

| || | |

1160 1170 1180 1190 1200

NNPAF GEHII KVMRA FDYVI RNLGK PKTLL AYLKN VGADH IARNV ERRHF

| | | | | | ||

1210 1220 1230 1240 1250

QAFSE ALIPV MQREL KAQLK PEAVA AWRKG LDRII GVIDQ GLLGL KEVNP

| | || | |

1260 1270 1280 1290 1300

QIAFS AADIE AIQKT WALAK PDLMG KGASV FRQLF TDHGY QPLFS NLVEY

| | |

1310 1320 1330 1340 1350

EVTGL EGSPE LNTHA RNVMA QLDTL VGSLQ NSIEL GKSLN QLGKD HVPRK

| | | ||

1360 1370 1380 1390 1400

VNKVH FDDFA EHFVP LMKAN LGDEF TPLAE SAWKK AFNVM VATIE QGQRA

| | || |

1410

RRSIA TFLTN PVA

||

array of base peptides:

legend

bpid base peptide id

cid chain id

c number of connectors for optional links

km number of known modifications

!! INFO here, calculated masses refer to unprotonated base peptides and take

!! INFO into account known modifications.

bpid cid nterm cterm c km m(calc) aa-sequence

-------------------------------------------------------------------------------

1 1 1 11 3 0 1134.559 AEISGILCSD K

2 1 12 15 1 0 431.274 ATIK

3 1 16 16 0 0 174.112 R

4 1 17 29 0 0 1449.725 TWATVTDLPS FGR

5 1 30 43 2 0 1611.866 NVFLSVFAAK PEYK

6 1 44 50 0 0 923.486 NLFVEFR

7 1 51 62 0 0 1272.631 NIPASELASS ER

8 1 63 69 0 0 814.445 LLYHGGR

9 1 70 86 0 0 1770.900 VLSSIDEAIA GIDTPDR

10 1 87 89 1 0 316.211 AVK

11 1 90 97 0 0 871.513 TLLALGER

12 1 98 101 0 0 511.287 HISR

13 1 102 105 0 0 431.249 GTVR

14 1 106 106 0 0 174.112 R

15 1 107 120 1 0 1715.819 HFEAFSYAFI DELK

16 1 121 122 0 0 302.170 QR

17 1 123 133 0 0 1173.578 GVESADLAAW R

18 1 134 134 0 0 174.112 R

19 1 135 149 0 0 1667.899 GWDNIVNVLE AGLLR

20 1 150 150 0 0 174.112 R

21 1 151 173 2 0 2533.237 QIDLEVTGLS CVDVANIQES WSK

22 1 174 179 1 0 617.338 VSGDLK

23 1 180 188 0 0 993.524 TTGSVVFQR

24 1 189 201 0 0 1631.788 MINGHPEYQQ LFR

25 1 202 204 0 0 449.239 QFR

26 1 205 210 1 0 703.339 DVDLDK

27 1 211 223 0 0 1461.736 LGESNSFVAH VFR

28 1 224 246 1 0 2542.343 VVAAFDGIIH ELDNNQFIVS TLK

29 1 247 247 1 0 146.106 K

30 1 248 255 0 0 922.498 LGEQHIAR

31 1 256 266 0 0 1320.621 GTDISHFQNF R

32 1 267 274 1 0 977.580 VTLLEYLK

33 1 275 283 1 0 947.413 ENGMNGAQK

34 1 284 288 1 0 604.297 ASWNK

35 1 289 295 1 0 826.386 AFDAFEK

36 1 296 305 1 0 1097.579 YISMGLSSLK

37 1 306 306 0 0 174.112 R

38 1 307 318 1 0 1227.671 VDPITGLSGL EK

39 1 319 327 1 0 988.534 NAILSTWGK

40 1 328 329 0 0 273.180 VR

41 1 330 337 1 0 843.445 GNLQEVGK

42 1 338 342 1 0 522.280 ATFGK

43 1 343 355 0 0 1666.792 LFTAHPEYQQ MFR

44 1 356 370 1 0 1589.812 FSQGMPLASL VESPK

45 1 371 377 0 0 829.419 FAAHTQR

46 1 378 400 1 0 2592.398 VVSALDQTLL ALNRPSDFVY MIK

47 1 401 409 0 0 1065.557 ELGLDHINR

48 1 410 413 0 0 447.208 GTDR

49 1 414 428 1 0 1914.951 SHFENYQVVF IEYLK

50 1 429 441 1 0 1452.698 ETLGDSLDEF TVK

51 1 442 459 0 0 2120.105 SFNHVFEVII SFLNEGLR

52 1 460 473 0 0 1520.795 QADIVDPVTH LTGR

53 1 474 475 1 0 274.164 QK

54 1 476 479 1 0 519.273 EMIK

55 1 480 484 1 0 577.286 ASWSK

56 1 485 486 0 0 245.149 AR

57 1 487 490 0 0 503.270 TDLR

58 1 491 499 0 0 1079.543 SLGQELFMR

59 1 500 502 1 0 424.214 MFK

60 1 503 514 1 0 1445.730 AHPEYQTLFV NK

61 1 515 525 0 0 1172.655 GFADVPLVSL R

62 1 526 529 0 0 547.224 EDER

63 1 530 559 0 0 3449.696 FISHMANVLG GFDTLLQNLD ESSYFIYSLR

64 1 560 568 0 0 1022.526 NLGDAHIQR

65 1 569 569 1 0 146.106 K

66 1 570 576 0 0 815.404 AGTQHFR

67 1 577 601 1 0 2764.396 SFEAILIPYL QESQGLDAAS VEAWK

68 1 602 602 1 0 146.106 K

69 1 603 616 1 0 1492.829 FFDVSIGVIA QGLK

70 1 617 632 1 0 1635.799 VATSEEADPV TGLYGK

71 1 633 638 0 0 699.428 EIVALR

72 1 639 647 0 0 959.519 QAFAAVTPR

73 1 648 653 1 0 658.365 NVEIGK

74 1 654 654 0 0 174.112 R

75 1 655 658 1 0 463.279 VFAK

76 1 659 667 1 0 1074.550 LFAAHPEYK

77 1 668 671 1 0 520.301 NLFK

78 1 672 672 1 0 146.106 K

79 1 673 697 0 0 3011.412 FEQYSVEELP STDAFHYHIS LVMNR

80 1 698 703 1 0 637.343 FSSIGK

81 1 704 716 1 0 1523.823 VIDDNVSFVY LLK

82 1 717 717 1 0 146.106 K

83 1 718 720 0 0 344.217 LGR

84 1 721 724 1 0 525.291 EHIK

85 1 725 725 0 0 174.112 R

86 1 726 729 0 0 431.249 GLSR

87 1 730 730 1 0 146.106 K

88 1 731 752 0 0 2546.217 QFDQFVELYI AEISSELSDT GR

89 1 753 757 1 0 559.297 NGLEK

90 1 758 786 1 0 3091.644 VLTFATGVIE QGLFQLGQVD SNTLTALEK

91 1 787 797 0 0 1358.694 QSIQDIWSNL R

92 1 798 807 1 0 1030.566 STGLQDLAVK

93 1 808 811 0 0 535.312 IFTR

94 1 812 820 1 0 1090.545 LFSAHPEYK

95 1 821 826 0 0 705.417 LLFTGR

96 1 827 840 1 0 1577.747 FGNVDNINEN APFK

97 1 841 845 0 0 632.351 AHLHR

98 1 846 865 0 0 2242.184 VLSAFDIVIS TLDDSEHLIR

99 1 866 868 1 0 387.248 QLK

100 1 869 876 0 0 957.503 DLGLFHTR

101 1 877 881 0 0 576.305 LGMTR

102 1 882 908 0 0 2931.488 SHFDNFATAF LSVAQDIAPN QLTVLGR

103 1 909 913 1 0 589.307 ESLNK

104 1 914 916 1 0 350.195 GFK

105 1 917 931 0 0 1735.929 LMHGVIEEGL LQLER

106 1 932 941 0 0 1040.598 INPITGLSAR

107 1 942 947 1 0 643.390 EVAVVK

108 1 948 963 1 0 1843.943 QTWNLVKPDL MGVGMR

109 1 964 966 1 0 406.258 IFK

110 1 967 981 1 0 1713.876 SLFEAFPAYQ AVFPK

111 1 982 989 1 0 919.465 FSDVPLDK

112 1 990 998 1 0 928.486 LEDTPAVGK

113 1 999 1006 1 0 855.481 HAISVTTK

114 1 1007 1025 0 0 2107.152 LDELIQTLDE PANLALLAR

115 1 1026 1035 0 0 1178.641 QLGEDHIVLR

116 1 1036 1042 2 0 862.473 VNKPMFK

117 1 1043 1046 1 0 437.227 SFGK

118 1 1047 1050 0 0 485.333 VLVR

119 1 1051 1059 0 0 1056.556 LLENDLGQR

120 1 1060 1066 0 0 800.382 FSSFASR

121 1 1067 1070 1 0 556.276 SWHK

122 1 1071 1088 1 0 2146.047 AYDVIVEYIE EGLQQSYK

123 1 1089 1100 1 0 1272.620 QDPVTGITDA EK

124 1 1101 1119 1 0 2122.178 ALVQESWDLL KPDLLGLGR

125 1 1120 1120 1 0 146.106 K

126 1 1121 1124 1 0 507.306 IFTK

127 1 1125 1128 1 0 493.290 VFTK

128 1 1129 1138 0 0 1288.656 HPDYQILFTR

129 1 1139 1148 1 0 1035.524 TGFGDTPLTK

130 1 1149 1161 1 0 1439.741 LDDNPAFGTH IIK

131 1 1162 1164 0 0 404.221 VMR

132 1 1165 1177 2 0 1464.845 AFDHVIQILG KPK

133 1 1178 1184 0 0 866.468 TLMAYLR

134 1 1185 1193 0 0 924.478 SVGADHIAR

135 1 1194 1197 0 0 516.266 NVER

136 1 1198 1198 0 0 174.112 R

137 1 1199 1216 1 0 2109.083 HFQAFSNALI PVMQHELK

138 1 1217 1228 0 0 1352.731 AQLRPDAVAA WR

139 1 1229 1229 1 0 146.106 K

140 1 1230 1233 0 0 459.244 GLDR

141 1 1234 1246 1 0 1351.844 IIGIIDQGLI GLK

142 1 1247 1264 0 0 2048.991 EVNPQNAFSA YDIQAVQR

143 1 1265 1276 2 0 1329.711 TWALAKPDLM GK

144 1 1277 1282 1 0 651.341 GAMVFK

145 1 1283 1316 0 0 3845.864 QLFTDHGYQP LFSNLAQYEI TGLEGSPELN

THAR

146 1 1317 1344 1 0 2926.543 NVMAQLDTLV GSLQNSIELG QSLAQLGK

147 1 1345 1349 0 0 622.319 DHVPR

148 1 1350 1350 1 0 146.106 K

149 1 1351 1353 0 0 387.223 VNR

150 1 1354 1357 1 0 529.301 VHFK

151 1 1358 1368 1 0 1346.669 DFAEHFIPLM K

152 1 1369 1384 1 0 1748.826 ADLGDEFTPL AESAWK

153 1 1385 1385 1 0 146.106 K

154 1 1386 1399 0 0 1577.787 AFDVMIATIE QGQR

155 1 1400 1401 0 0 245.149 AR

156 1 1402 1402 0 0 174.112 R

157 1 1403 1413 0 0 1118.597 SVATFLTNPV A

158 2 1 4 1 0 473.260 AEVR

159 2 5 11 2 0 734.363 GILCSDK

160 2 12 15 1 0 431.274 ATIK

161 2 16 16 0 0 174.112 R

162 2 17 29 0 0 1490.752 TWSIVNDLPS FGR

163 2 30 43 2 0 1611.866 NVFLSVFAAK PEYK

164 2 44 50 0 0 923.486 NLFVEFR

165 2 51 62 0 0 1299.642 NIPASELANS ER

166 2 63 69 0 0 814.445 LLYHGGR

167 2 70 89 1 0 2058.037 VLASIDEVIS EIDSPDSAAK

168 2 90 90 1 0 146.106 K

169 2 91 97 0 0 756.449 LVALGER

170 2 98 101 0 0 525.302 HITR

171 2 102 105 0 0 431.249 GTVR

172 2 106 106 0 0 174.112 R

173 2 107 120 1 0 1715.819 HFEAFSYAFI DELK

174 2 121 122 0 0 302.170 QR

175 2 123 133 0 0 1115.572 GVASADLAAW R

176 2 134 134 1 0 146.106 K

177 2 135 149 1 0 1627.882 GWDSIVDILE AGLLK

178 2 150 150 0 0 174.112 R

179 2 151 179 2 0 3088.538 QIDLEVTGLS CVDVANVQES WATVSANLK

180 2 180 188 0 0 1034.551 NTGSILFQR

181 2 189 201 0 0 1671.837 LINDHPEYQQ LFR

182 2 202 204 0 0 449.239 QFR

183 2 205 210 1 0 673.365 DVELAK

184 2 211 223 0 0 1431.726 LGESNGFVAH VFR

185 2 224 233 1 0 1031.601 VVAAFDGIIK

186 2 234 246 1 0 1488.782 ELDNNPFIVS TLK

187 2 247 247 0 0 174.112 R

188 2 248 255 0 0 922.498 LGEQHIAR

189 2 256 266 0 0 1320.621 GTDISHFQNF R

190 2 267 288 1 0 2523.206 TTLLVYLNEN GMNQAQEASW NK

191 2 289 295 1 0 792.402 AFDAIEK

192 2 296 302 1 0 792.474 YISIGLK

193 2 303 306 0 0 431.249 SLGR

194 2 307 318 1 0 1227.671 VDPITGLSGL EK

195 2 319 327 1 0 1015.545 NAILNTWGK

196 2 328 329 0 0 273.180 VR

197 2 330 337 1 0 843.445 GNLQEVGK

198 2 338 342 1 0 522.280 ATFGK

199 2 343 355 0 0 1636.782 LFAAHPEYQQ MFR

200 2 356 370 1 0 1676.877 FFQGVQLAEL VDSPK

201 2 371 377 0 0 829.419 FAAHTQR

202 2 378 400 1 0 2592.398 VVSALDQTLL ALNRPSDFVY MIK

203 2 401 409 0 0 1065.557 ELGLDHINR

204 2 410 413 0 0 447.208 GTDR

205 2 414 428 1 0 1900.936 SHFENYQVVF VEYLK

206 2 429 441 1 0 1438.683 ETLGDSVDEF TVK

207 2 442 459 0 0 2147.116 SFNHVFEVII NFLNEGLR

208 2 460 473 0 0 1505.795 QANVVDPVTH LTGR

209 2 474 475 1 0 274.164 QK

210 2 476 479 1 0 459.269 EAIK

211 2 480 486 0 0 775.398 ASWSVAR

212 2 487 490 0 0 503.270 TDLR

213 2 491 499 0 0 1139.580 FLGQELFMR

214 2 500 514 1 0 1842.897 MFNLNPEYQS LFVNK

215 2 515 525 0 0 1172.655 GFADVPLVSL R

216 2 526 529 0 0 547.224 EDER

217 2 530 539 0 0 1186.628 FISHMANVLR

218 2 540 559 0 0 2350.148 GFDTLLQNLD DTSYFVYALR

219 2 560 568 0 0 1022.526 NLGDAHIQR

220 2 569 569 1 0 146.106 K

221 2 570 576 0 0 816.388 AGTEHFR

222 2 577 601 1 0 2734.385 SFEAILIPYL QESQGLDAAG VEAWK

223 2 602 616 1 0 1605.913 IFFDVSIGVI AQGLK

224 2 617 632 1 0 1621.783 VASSEEADPV TGLYGK

225 2 633 638 0 0 685.412 EVVALR

226 2 639 647 0 0 959.519 QAFAAISPR

227 2 648 653 1 0 658.365 NVEIGK

228 2 654 654 0 0 174.112 R

229 2 655 658 1 0 463.279 VFAK

230 2 659 667 1 0 1120.555 LFTSHPEYK

231 2 668 671 1 0 520.301 NLFK

232 2 672 672 1 0 146.106 K

233 2 673 697 0 0 2989.380 FEQYSVEELP STDAFDYHIS LVMNR

234 2 698 703 1 0 607.333 FSAVGK

235 2 704 716 1 0 1523.823 VIDDNVSFVY LLK

236 2 717 717 1 0 146.106 K

237 2 718 720 0 0 344.217 LGR

238 2 721 724 1 0 525.291 EHIK

239 2 725 725 0 0 174.112 R

240 2 726 729 0 0 431.249 GLSR

241 2 730 730 1 0 146.106 K

242 2 731 752 0 0 2570.254 QFDQFVELYI AEISPELSET GR

243 2 753 757 1 0 532.286 SGLEK

244 2 758 779 1 0 2349.258 VLTFATGVIE QGLFQLGQVD SK

245 2 780 786 1 0 744.438 ALTALEK

246 2 787 807 1 0 2383.274 QSIQDIWTSL RPTGLEELAV K

247 2 808 811 0 0 553.268 MFTR

248 2 812 820 1 0 1118.540 LFADHPEYK

249 2 821 826 0 0 705.417 LLFTGR

250 2 827 840 0 0 1571.769 LGNVDNINEN APFR

251 2 841 845 0 0 632.351 AHLHR

252 2 846 865 0 0 2186.231 VLSAFDIVIT SLDNNALLIR

253 2 866 868 1 0 387.248 QLK

254 2 869 876 0 0 957.503 DLGLFHTR

255 2 877 881 0 0 576.305 LGMTR

256 2 882 908 0 0 2935.487 AHFDNFATAF FSVAEDIVPN LLTALGR

257 2 909 913 1 0 532.286 ESLGK

258 2 914 916 1 0 350.195 GFK

259 2 917 931 0 0 1711.954 LMVAVIEEGL LQLER

260 2 932 941 0 0 1069.613 IDPITGLSVR

261 2 942 947 1 0 701.396 EVEVVK

262 2 948 963 1 0 1843.943 QTWNLVKPDL MGVGMR

263 2 964 966 1 0 406.258 IFK

264 2 967 971 1 0 622.333 SLFEK

265 2 972 981 1 0 1166.612 FPAYQAVFPK

266 2 982 989 1 0 919.465 FSDVPLDK

267 2 990 998 1 0 940.523 LEDIPAVGK

268 2 999 1006 1 0 855.481 HAISVTTK

269 2 1007 1025 0 0 2107.152 LDELIQTLDE PANLALLAR

270 2 1026 1042 2 0 1924.024 QLGEDHIVLG VNKPMFK

271 2 1043 1050 0 0 905.497 SFGEVLVR

272 2 1051 1059 0 0 1056.556 LLENDLGQR

273 2 1060 1066 1 0 799.386 FSNFASK

274 2 1067 1070 0 0 584.282 SWHR

275 2 1071 1088 1 0 2146.047 AYDVIVEYIE EGLQQSYK

276 2 1089 1100 1 0 1272.620 QDPVTGITDA EK

277 2 1101 1105 0 0 613.391 VLVQR

278 2 1106 1119 1 0 1595.903 SWELLKPDLL GLGR

279 2 1120 1120 1 0 146.106 K

280 2 1121 1128 1 0 923.548 IFGVIFTK

281 2 1129 1138 0 0 1302.672 HPEYQILFTR

282 2 1139 1161 1 0 2482.249 VGFGDTPLTQ LDNNPAFGEH IIK

283 2 1162 1164 0 0 404.221 VMR

284 2 1165 1171 0 0 882.460 AFDYVIR

285 2 1172 1177 2 0 655.402 NLGKPK

286 2 1178 1184 1 0 820.506 TLLAYLK

287 2 1185 1193 0 0 951.489 NVGADHIAR

288 2 1194 1197 0 0 516.266 NVER

289 2 1198 1198 0 0 174.112 R

290 2 1199 1213 0 0 1772.903 HFQAFSEALI PVMQR

291 2 1214 1216 1 0 388.232 ELK

292 2 1217 1228 1 0 1338.741 AQLKPEAVAA WR

293 2 1229 1229 1 0 146.106 K

294 2 1230 1233 0 0 459.244 GLDR

295 2 1234 1246 1 0 1337.828 IIGVIDQGLL GLK

296 2 1247 1264 1 0 1943.000 EVNPQIAFSA ADIEAIQK

297 2 1265 1276 2 0 1329.711 TWALAKPDLM GK

298 2 1277 1282 0 0 635.339 GASVFR

299 2 1283 1316 0 0 3860.864 QLFTDHGYQP LFSNLVEYEV TGLEGSPELN

THAR

300 2 1317 1337 1 0 2229.167 NVMAQLDTLV GSLQNSIELG K

301 2 1338 1344 1 0 758.429 SLNQLGK

302 2 1345 1349 0 0 622.319 DHVPR

303 2 1350 1350 1 0 146.106 K

304 2 1351 1353 1 0 359.217 VNK

305 2 1354 1368 1 0 1830.876 VHFDDFAEHF VPLMK

306 2 1369 1384 1 0 1747.842 ANLGDEFTPL AESAWK

307 2 1385 1385 1 0 146.106 K

308 2 1386 1399 0 0 1562.787 AFNVMVATIE QGQR

309 2 1400 1401 0 0 245.149 AR

310 2 1402 1402 0 0 174.112 R

311 2 1403 1413 0 0 1132.613 SIATFLTNPV A

[sorted list of assignments](http://www.searchxlinks.de/cgi-bin/upload_action.pl#contents%23contents)

**************************

legend

d deviation experimentally determined mass - calculated mass

pid peptide id

om number of optional modifications

kl number of known links

ol number of optional links

sp number of subpeptides obtained after removing ALL links

i:m-n linear subpeptide extending from residue m to residue n of chain i

number of peaks assigned 19

number of peptides assigned 35

number of assignments 35

18 peak(s) with NO peptides assigned:

m(exp)

---------

802.350

826.340

846.390

858.330

861.320

889.385

930.305

938.400

982.450

1012.365

1053.450

1117.440

1139.470

1147.455

1209.445

1243.530

1340.520

1515.545

8 peak(s) with 1 peptide(s) assigned:

m(exp) m(calc) d[ppm] pid om kl ol sp residues

-------------------------------------------------------------------------------

803.360 803.394 42.1 [28](http://www.searchxlinks.de/cgi-bin/upload_action.pl#f28%23f28) 1 0 0 1 1:698-703

899.345 899.521 196.2 [32](http://www.searchxlinks.de/cgi-bin/upload_action.pl#f32%23f32) 0 0 0 1 1:1351-1357

952.330 952.496 174.6 [15](http://www.searchxlinks.de/cgi-bin/upload_action.pl#f15%23f15) 0 0 0 1 2:1185-1193

1031.380 1031.574 187.7 [8](http://www.searchxlinks.de/cgi-bin/upload_action.pl#f8%23f8) 0 0 0 1 1:798-807

1195.400 1195.563 136.7 [31](http://www.searchxlinks.de/cgi-bin/upload_action.pl#f31%23f31) 2 0 0 1 2:909-916

1381.305 1381.667 262.1 [23](http://www.searchxlinks.de/cgi-bin/upload_action.pl#f23%23f23) 1 0 1 2 1:87-89 2:5-11

1413.545 1413.680 95.6 [21](http://www.searchxlinks.de/cgi-bin/upload_action.pl#f21%23f21) 0 0 0 1 1:284-295

1531.465 1531.708 158.7 [22](http://www.searchxlinks.de/cgi-bin/upload_action.pl#f22%23f22) 1 0 0 1 2:1060-1070

8 peak(s) with 2 peptide(s) assigned:

m(exp) m(calc) d[ppm] pid om kl ol sp residues

-------------------------------------------------------------------------------

824.375 824.415 48.9 [26](http://www.searchxlinks.de/cgi-bin/upload_action.pl#f26%23f26) 1 0 0 1 1:648-653

824.415 48.9 [27](http://www.searchxlinks.de/cgi-bin/upload_action.pl#f27%23f27) 1 0 0 1 2:648-653

844.360 844.453 109.8 [19](http://www.searchxlinks.de/cgi-bin/upload_action.pl#f19%23f19) 0 0 0 1 1:330-337

844.453 109.8 [20](http://www.searchxlinks.de/cgi-bin/upload_action.pl#f20%23f20) 0 0 0 1 2:330-337

867.285 867.446 185.9 [25](http://www.searchxlinks.de/cgi-bin/upload_action.pl#f25%23f25) 1 0 0 1 2:942-947

867.476 220.3 [18](http://www.searchxlinks.de/cgi-bin/upload_action.pl#f18%23f18) 0 0 0 1 1:1178-1184

916.370 916.464 102.5 [29](http://www.searchxlinks.de/cgi-bin/upload_action.pl#f29%23f29) 1 0 0 1 1:1345-1350

916.464 102.5 [30](http://www.searchxlinks.de/cgi-bin/upload_action.pl#f30%23f30) 1 0 0 1 2:1345-1350

923.450 923.506 60.8 [16](http://www.searchxlinks.de/cgi-bin/upload_action.pl#f16%23f16) 0 0 0 1 1:248-255

923.506 60.8 [17](http://www.searchxlinks.de/cgi-bin/upload_action.pl#f17%23f17) 0 0 0 1 2:248-255

960.370 960.527 163.0 [11](http://www.searchxlinks.de/cgi-bin/upload_action.pl#f11%23f11) 0 0 0 1 2:639-647

960.527 163.0 [12](http://www.searchxlinks.de/cgi-bin/upload_action.pl#f12%23f12) 0 0 0 1 1:639-647

1023.505 1023.533 27.8 [9](http://www.searchxlinks.de/cgi-bin/upload_action.pl#f9%23f9) 0 0 0 1 2:560-568

1023.533 27.8 [10](http://www.searchxlinks.de/cgi-bin/upload_action.pl#f10%23f10) 0 0 0 1 1:560-568

1070.315 1070.600 266.0 [35](http://www.searchxlinks.de/cgi-bin/upload_action.pl#f35%23f35) 1 0 0 1 1:1043-1050

1070.621 285.7 [7](http://www.searchxlinks.de/cgi-bin/upload_action.pl#f7%23f7) 0 0 0 1 2:932-941

1 peak(s) with 3 peptide(s) assigned:

m(exp) m(calc) d[ppm] pid om kl ol sp residues

-------------------------------------------------------------------------------

1493.535 1492.791 498.3 [13](http://www.searchxlinks.de/cgi-bin/upload_action.pl#f13%23f13) 1 0 0 1 2:866-876

1492.791 498.3 [14](http://www.searchxlinks.de/cgi-bin/upload_action.pl#f14%23f14) 1 0 0 1 1:866-876

1493.837 201.9 [1](http://www.searchxlinks.de/cgi-bin/upload_action.pl#f1%23f1) 0 0 0 1 1:603-616

2 peak(s) with 4 peptide(s) assigned:

m(exp) m(calc) d[ppm] pid om kl ol sp residues

-------------------------------------------------------------------------------

1173.315 1173.649 284.7 [33](http://www.searchxlinks.de/cgi-bin/upload_action.pl#f33%23f33) 1 0 0 1 1:718-725

1173.649 284.7 [34](http://www.searchxlinks.de/cgi-bin/upload_action.pl#f34%23f34) 1 0 0 1 2:718-725

1173.663 296.6 [3](http://www.searchxlinks.de/cgi-bin/upload_action.pl#f3%23f3) 0 0 0 1 2:515-525

1173.663 296.6 [4](http://www.searchxlinks.de/cgi-bin/upload_action.pl#f4%23f4) 0 0 0 1 1:515-525

1300.645 1300.609 27.4 [5](http://www.searchxlinks.de/cgi-bin/upload_action.pl#f5%23f5) 1 0 0 1 1:1-11

1300.609 27.4 [6](http://www.searchxlinks.de/cgi-bin/upload_action.pl#f6%23f6) 0 0 1 1 1:1-11

1300.617 21.4 [24](http://www.searchxlinks.de/cgi-bin/upload_action.pl#f24%23f24) 1 0 0 1 1:202-210

1300.650 3.5 [2](http://www.searchxlinks.de/cgi-bin/upload_action.pl#f2%23f2) 0 0 0 1 2:51-62

[peptide isomers](http://www.searchxlinks.de/cgi-bin/upload_action.pl#contents%23contents)

***************

legend

[ n-terminal NH2 group

[[ n-terminal NH2 group (side chain blocked)

] c-terminal COOH group

]] c-terminal COOH group (side chain blocked)

k known cross-link/modification

o optional cross-link/modification

L left side of a cross-linker

R right side of a cross-linker

x cross-linker (left or right side)

~ missed cleavage

= missed cleavage (mandatory)

legend for detailed list of psd assignments

c intensity class of assigned psd peak (i=intense,w=weak)

*******************************************************************************

peptide 1

number of base peptides = 1

number of missed cleavages = 0

number of optional links = 0

number of known links = 0

number of optional modifications = 0

number of known modifications = 0

calculated mass = 1493.8366

...............................................................................

isomer 1 / peptide 1

1F603--1K616

*** [legend](http://www.searchxlinks.de/cgi-bin/upload_action.pl#peptide_isomers%23peptide_isomers) ********************************************************************

peptide 2

number of base peptides = 1

number of missed cleavages = 0

number of optional links = 0

number of known links = 0

number of optional modifications = 0

number of known modifications = 0

calculated mass = 1300.6496

...............................................................................

isomer 1 / peptide 2

2N51--2R62

*** [legend](http://www.searchxlinks.de/cgi-bin/upload_action.pl#peptide_isomers%23peptide_isomers) ********************************************************************

peptide 3

number of base peptides = 1

number of missed cleavages = 0

number of optional links = 0

number of known links = 0

number of optional modifications = 0

number of known modifications = 0

calculated mass = 1173.6631

...............................................................................

isomer 1 / peptide 3

2G515--2R525

*** [legend](http://www.searchxlinks.de/cgi-bin/upload_action.pl#peptide_isomers%23peptide_isomers) ********************************************************************

peptide 4

number of base peptides = 1

number of missed cleavages = 0

number of optional links = 0

number of known links = 0

number of optional modifications = 0

number of known modifications = 0

calculated mass = 1173.6631

...............................................................................

isomer 1 / peptide 4

1G515--1R525

*** [legend](http://www.searchxlinks.de/cgi-bin/upload_action.pl#peptide_isomers%23peptide_isomers) ********************************************************************

peptide 5

number of base peptides = 1

number of missed cleavages = 0

number of optional links = 0

number of known links = 0

number of optional modifications = 1

number of known modifications = 0

calculated mass = 1300.6093

...............................................................................

isomer 1 / peptide 5

1[1--1K11

o

L

...............................................................................

isomer 2 / peptide 5

1A1--1K11

o

L

*** [legend](http://www.searchxlinks.de/cgi-bin/upload_action.pl#peptide_isomers%23peptide_isomers) ********************************************************************

peptide 6

number of base peptides = 1

number of missed cleavages = 0

number of optional links = 1

number of known links = 0

number of optional modifications = 0

number of known modifications = 0

calculated mass = 1300.6093

...............................................................................

isomer 1 / peptide 6

oooooo

1[1--1C8--1K11

...............................................................................

isomer 2 / peptide 6

ooooooo

1A1--1C8--1K11

*** [legend](http://www.searchxlinks.de/cgi-bin/upload_action.pl#peptide_isomers%23peptide_isomers) ********************************************************************

peptide 7

number of base peptides = 1

number of missed cleavages = 0

number of optional links = 0

number of known links = 0

number of optional modifications = 0

number of known modifications = 0

calculated mass = 1070.6209

...............................................................................

isomer 1 / peptide 7

2I932--2R941

*** [legend](http://www.searchxlinks.de/cgi-bin/upload_action.pl#peptide_isomers%23peptide_isomers) ********************************************************************

peptide 8

number of base peptides = 1

number of missed cleavages = 0

number of optional links = 0

number of known links = 0

number of optional modifications = 0

number of known modifications = 0

calculated mass = 1031.5736

...............................................................................

isomer 1 / peptide 8

1S798--1K807

*** [legend](http://www.searchxlinks.de/cgi-bin/upload_action.pl#peptide_isomers%23peptide_isomers) ********************************************************************

peptide 9

number of base peptides = 1

number of missed cleavages = 0

number of optional links = 0

number of known links = 0

number of optional modifications = 0

number of known modifications = 0

calculated mass = 1023.5334

...............................................................................

isomer 1 / peptide 9

2N560--2R568

*** [legend](http://www.searchxlinks.de/cgi-bin/upload_action.pl#peptide_isomers%23peptide_isomers) ********************************************************************

peptide 10

number of base peptides = 1

number of missed cleavages = 0

number of optional links = 0

number of known links = 0

number of optional modifications = 0

number of known modifications = 0

calculated mass = 1023.5334

...............................................................................

isomer 1 / peptide 10

1N560--1R568

*** [legend](http://www.searchxlinks.de/cgi-bin/upload_action.pl#peptide_isomers%23peptide_isomers) ********************************************************************

peptide 11

number of base peptides = 1

number of missed cleavages = 0

number of optional links = 0

number of known links = 0

number of optional modifications = 0

number of known modifications = 0

calculated mass = 960.5266

...............................................................................

isomer 1 / peptide 11

2Q639--2R647

*** [legend](http://www.searchxlinks.de/cgi-bin/upload_action.pl#peptide_isomers%23peptide_isomers) ********************************************************************

peptide 12

number of base peptides = 1

number of missed cleavages = 0

number of optional links = 0

number of known links = 0

number of optional modifications = 0

number of known modifications = 0

calculated mass = 960.5266

...............................................................................

isomer 1 / peptide 12

1Q639--1R647

*** [legend](http://www.searchxlinks.de/cgi-bin/upload_action.pl#peptide_isomers%23peptide_isomers) ********************************************************************

peptide 13

number of base peptides = 2

number of missed cleavages = 1

number of optional links = 0

number of known links = 0

number of optional modifications = 1

number of known modifications = 0

calculated mass = 1492.7911

...............................................................................

isomer 1 / peptide 13

2Q866--2K868 ~ 2D869--2R876

o

L

*** [legend](http://www.searchxlinks.de/cgi-bin/upload_action.pl#peptide_isomers%23peptide_isomers) ********************************************************************

peptide 14

number of base peptides = 2

number of missed cleavages = 1

number of optional links = 0

number of known links = 0

number of optional modifications = 1

number of known modifications = 0

calculated mass = 1492.7911

...............................................................................

isomer 1 / peptide 14

1Q866--1K868 ~ 1D869--1R876

o

L

*** [legend](http://www.searchxlinks.de/cgi-bin/upload_action.pl#peptide_isomers%23peptide_isomers) ********************************************************************

peptide 15

number of base peptides = 1

number of missed cleavages = 0

number of optional links = 0

number of known links = 0

number of optional modifications = 0

number of known modifications = 0

calculated mass = 952.4963

...............................................................................

isomer 1 / peptide 15

2N1185--2R1193

*** [legend](http://www.searchxlinks.de/cgi-bin/upload_action.pl#peptide_isomers%23peptide_isomers) ********************************************************************

peptide 16

number of base peptides = 1

number of missed cleavages = 0

number of optional links = 0

number of known links = 0

number of optional modifications = 0

number of known modifications = 0

calculated mass = 923.5062

...............................................................................

isomer 1 / peptide 16

1L248--1R255

*** [legend](http://www.searchxlinks.de/cgi-bin/upload_action.pl#peptide_isomers%23peptide_isomers) ********************************************************************

peptide 17

number of base peptides = 1

number of missed cleavages = 0

number of optional links = 0

number of known links = 0

number of optional modifications = 0

number of known modifications = 0

calculated mass = 923.5062

...............................................................................

isomer 1 / peptide 17

2L248--2R255

*** [legend](http://www.searchxlinks.de/cgi-bin/upload_action.pl#peptide_isomers%23peptide_isomers) ********************************************************************

peptide 18

number of base peptides = 1

number of missed cleavages = 0

number of optional links = 0

number of known links = 0

number of optional modifications = 0

number of known modifications = 0

calculated mass = 867.4761

...............................................................................

isomer 1 / peptide 18

1T1178--1R1184

*** [legend](http://www.searchxlinks.de/cgi-bin/upload_action.pl#peptide_isomers%23peptide_isomers) ********************************************************************

peptide 19

number of base peptides = 1

number of missed cleavages = 0

number of optional links = 0

number of known links = 0

number of optional modifications = 0

number of known modifications = 0

calculated mass = 844.4527

...............................................................................

isomer 1 / peptide 19

1G330--1K337

*** [legend](http://www.searchxlinks.de/cgi-bin/upload_action.pl#peptide_isomers%23peptide_isomers) ********************************************************************

peptide 20

number of base peptides = 1

number of missed cleavages = 0

number of optional links = 0

number of known links = 0

number of optional modifications = 0

number of known modifications = 0

calculated mass = 844.4527

...............................................................................

isomer 1 / peptide 20

2G330--2K337

*** [legend](http://www.searchxlinks.de/cgi-bin/upload_action.pl#peptide_isomers%23peptide_isomers) ********************************************************************

peptide 21

number of base peptides = 2

number of missed cleavages = 1

number of optional links = 0

number of known links = 0

number of optional modifications = 0

number of known modifications = 0

calculated mass = 1413.6801

...............................................................................

isomer 1 / peptide 21

1A284--1K288 ~ 1A289--1K295

*** [legend](http://www.searchxlinks.de/cgi-bin/upload_action.pl#peptide_isomers%23peptide_isomers) ********************************************************************

peptide 22

number of base peptides = 2

number of missed cleavages = 1

number of optional links = 0

number of known links = 0

number of optional modifications = 1

number of known modifications = 0

calculated mass = 1531.7081

...............................................................................

isomer 1 / peptide 22

2F1060--2K1066 ~ 2S1067--2R1070

o

L

*** [legend](http://www.searchxlinks.de/cgi-bin/upload_action.pl#peptide_isomers%23peptide_isomers) ********************************************************************

peptide 23

number of base peptides = 2

number of missed cleavages = 0

number of optional links = 1

number of known links = 0

number of optional modifications = 1

number of known modifications = 0

calculated mass = 1381.6672

...............................................................................

isomer 1 / peptide 23

oooooooooooo

1A87--1K89 2G5--2C8--2K11

o

L

. . . . . . . . . . . . . . . . . . . . . . . . . . . . . . . . . . . . . . . .

psd analysis for psd peak list "1" (selected parent mass = 1381.305)

concise lists of psd assignments

simple chain fragmentation

chain 1 1 2 2

----- - - - -

8 8 0 1

7 9 5 1

rule A V K G I L C S D K score

a 0.0

b 0.0

b' 0.0

b" 0.0

b-NH3 0.0

b-H2O 0.0

b(n-1)+H2O 0.0

y [6](http://www.searchxlinks.de/cgi-bin/upload_action.pl#c1f23p1a3%23c1f23p1a3) [4](http://www.searchxlinks.de/cgi-bin/upload_action.pl#c1f23p1a6%23c1f23p1a6) 4.0

y' 0.0

y" 0.0

y-NH3 0.0

y-H2O [2](http://www.searchxlinks.de/cgi-bin/upload_action.pl#c1f23p1a1%23c1f23p1a1) [1](http://www.searchxlinks.de/cgi-bin/upload_action.pl#c1f23p1a2%23c1f23p1a2) [6](http://www.searchxlinks.de/cgi-bin/upload_action.pl#c1f23p1a4%23c1f23p1a4) [5](http://www.searchxlinks.de/cgi-bin/upload_action.pl#c1f23p1a5%23c1f23p1a5) [4](http://www.searchxlinks.de/cgi-bin/upload_action.pl#c1f23p1a7%23c1f23p1a7) 20.0

linker fragmentation

no assignments of linker fragments

detailed list of psd assignments

number of unassigned psd peaks 33

number of psd peaks assigned 6

number of psd fragments assigned 7

number of psd assignments 7

total score 24.0

m(exp) m(calc) d[Da] score psd rule fragmentation site(s)

-------------------------------------------------------------------------------

1080.000 1080.467 0.5 8.0 y-H2O4 2L7-[2C8]

1100.000 1098.478 1.5 2.0 y4 2L7-[2C8]

1193.000 1193.551 0.6 4.0 y-H2O1 1V88-[1K89]

1193.000 1193.551 0.6 4.0 y-H2O5 2I6-[2L7]

1292.000 1292.620 0.6 2.0 y-H2O2 1A87-[1V88]

1306.000 1306.635 0.6 2.0 y-H2O6 2G5-[2I6]

1324.000 1324.646 0.6 2.0 y6 2G5-[2I6]

*** [legend](http://www.searchxlinks.de/cgi-bin/upload_action.pl#peptide_isomers%23peptide_isomers) ********************************************************************

peptide 24

number of base peptides = 2

number of missed cleavages = 1

number of optional links = 0

number of known links = 0

number of optional modifications = 1

number of known modifications = 0

calculated mass = 1300.6172

...............................................................................

isomer 1 / peptide 24

1Q202--1R204 ~ 1D205--1K210

o

L

*** [legend](http://www.searchxlinks.de/cgi-bin/upload_action.pl#peptide_isomers%23peptide_isomers) ********************************************************************

peptide 25

number of base peptides = 1

number of missed cleavages = 0

number of optional links = 0

number of known links = 0

number of optional modifications = 1

number of known modifications = 0

calculated mass = 867.4463

...............................................................................

isomer 1 / peptide 25

2E942--2K947

o

L

*** [legend](http://www.searchxlinks.de/cgi-bin/upload_action.pl#peptide_isomers%23peptide_isomers) ********************************************************************

peptide 26

number of base peptides = 1

number of missed cleavages = 0

number of optional links = 0

number of known links = 0

number of optional modifications = 1

number of known modifications = 0

calculated mass = 824.4153

...............................................................................

isomer 1 / peptide 26

1N648--1K653

o

L

*** [legend](http://www.searchxlinks.de/cgi-bin/upload_action.pl#peptide_isomers%23peptide_isomers) ********************************************************************

peptide 27

number of base peptides = 1

number of missed cleavages = 0

number of optional links = 0

number of known links = 0

number of optional modifications = 1

number of known modifications = 0

calculated mass = 824.4153

...............................................................................

isomer 1 / peptide 27

2N648--2K653

o

L

*** [legend](http://www.searchxlinks.de/cgi-bin/upload_action.pl#peptide_isomers%23peptide_isomers) ********************************************************************

peptide 28

number of base peptides = 1

number of missed cleavages = 0

number of optional links = 0

number of known links = 0

number of optional modifications = 1

number of known modifications = 0

calculated mass = 803.3939

...............................................................................

isomer 1 / peptide 28

1F698--1K703

o

L

*** [legend](http://www.searchxlinks.de/cgi-bin/upload_action.pl#peptide_isomers%23peptide_isomers) ********************************************************************

peptide 29

number of base peptides = 2

number of missed cleavages = 1

number of optional links = 0

number of known links = 0

number of optional modifications = 1

number of known modifications = 0

calculated mass = 916.4640

...............................................................................

isomer 1 / peptide 29

1D1345--1R1349 ~ 1K1350

o

L

*** [legend](http://www.searchxlinks.de/cgi-bin/upload_action.pl#peptide_isomers%23peptide_isomers) ********************************************************************

peptide 30

number of base peptides = 2

number of missed cleavages = 1

number of optional links = 0

number of known links = 0

number of optional modifications = 1

number of known modifications = 0

calculated mass = 916.4640

...............................................................................

isomer 1 / peptide 30

2D1345--2R1349 ~ 2K1350

o

L

*** [legend](http://www.searchxlinks.de/cgi-bin/upload_action.pl#peptide_isomers%23peptide_isomers) ********************************************************************

peptide 31

number of base peptides = 2

number of missed cleavages = 1

number of optional links = 0

number of known links = 0

number of optional modifications = 2

number of known modifications = 0

calculated mass = 1195.5634

...............................................................................

isomer 1 / peptide 31

2E909--2K913 ~ 2G914--2K916

o o

L L

*** [legend](http://www.searchxlinks.de/cgi-bin/upload_action.pl#peptide_isomers%23peptide_isomers) ********************************************************************

peptide 32

number of base peptides = 2

number of missed cleavages = 1

number of optional links = 0

number of known links = 0

number of optional modifications = 0

number of known modifications = 0

calculated mass = 899.5214

...............................................................................

isomer 1 / peptide 32

1V1351--1R1353 ~ 1V1354--1K1357

*** [legend](http://www.searchxlinks.de/cgi-bin/upload_action.pl#peptide_isomers%23peptide_isomers) ********************************************************************

peptide 33

number of base peptides = 3

number of missed cleavages = 2

number of optional links = 0

number of known links = 0

number of optional modifications = 1

number of known modifications = 0

calculated mass = 1173.6491

...............................................................................

isomer 1 / peptide 33

1L718--1R720 ~ 1E721--1K724 ~ 1R725

o

L

*** [legend](http://www.searchxlinks.de/cgi-bin/upload_action.pl#peptide_isomers%23peptide_isomers) ********************************************************************

peptide 34

number of base peptides = 3

number of missed cleavages = 2

number of optional links = 0

number of known links = 0

number of optional modifications = 1

number of known modifications = 0

calculated mass = 1173.6491

...............................................................................

isomer 1 / peptide 34

2L718--2R720 ~ 2E721--2K724 ~ 2R725

o

L

*** [legend](http://www.searchxlinks.de/cgi-bin/upload_action.pl#peptide_isomers%23peptide_isomers) ********************************************************************

peptide 35

number of base peptides = 2

number of missed cleavages = 1

number of optional links = 0

number of known links = 0

number of optional modifications = 1

number of known modifications = 0

calculated mass = 1070.5997

...............................................................................

isomer 1 / peptide 35

1S1043--1K1046 ~ 1V1047--1R1050

o

L

*** [legend](http://www.searchxlinks.de/cgi-bin/upload_action.pl#peptide_isomers%23peptide_isomers) ********************************************************************

[psd analysis (summary)](http://www.searchxlinks.de/cgi-bin/upload_action.pl#contents%23contents)

**********************

legend

iid isomer id

pid peptide id

d deviation selected psd parent mass - exp. parent peak mass

selected psd parent mass = 1381.305

psd peak list label = 1

exp.

assigned parent

rank iid/pid score peaks peak mass d[Da]

-------------------------------------------------------

1 [1/23](http://www.searchxlinks.de/cgi-bin/upload_action.pl#c1f23p1%23c1f23p1) 24.0 6/39 1381.305 0.0

[protein coverage](http://www.searchxlinks.de/cgi-bin/upload_action.pl#contents%23contents)

****************

legend

* covered residue (occurs in at least one peptide)

number of covered residues: 302

total number of residues: 2826

coverage [%]: 11

...............................................................................

chain 1 (chain 1 of protein 1)

number of covered residues: 163

total number of residues: 1413

coverage [%]: 12

10 20 30 40 50

AEISG ILCSD KATIK RTWAT VTDLP SFGRN VFLSV FAAKP EYKNL FVEFR

***** ***** *

60 70 80 90 100

NIPAS ELASS ERLLY HGGRV LSSID EAIAG IDTPD RAVKT LLALG ERHIS

***

110 120 130 140 150

RGTVR RHFEA FSYAF IDELK QRGVE SADLA AWRRG WDNIV NVLEA GLLRR

160 170 180 190 200

QIDLE VTGLS CVDVA NIQES WSKVS GDLKT TGSVV FQRMI NGHPE YQQLF

210 220 230 240 250

RQFRD VDLDK LGESN SFVAH VFRVV AAFDG IIHEL DNNQF IVSTL KKLGE

**** ***** ***

260 270 280 290 300

QHIAR GTDIS HFQNF RVTLL EYLKE NGMNG AQKAS WNKAF DAFEK YISMG

***** ** ***** *****

310 320 330 340 350

LSSLK RVDPI TGLSG LEKNA ILSTW GKVRG NLQEV GKATF GKLFT AHPEY

* ***** **

360 370 380 390 400

QQMFR FSQGM PLASL VESPK FAAHT QRVVS ALDQT LLALN RPSDF VYMIK

410 420 430 440 450

ELGLD HINRG TDRSH FENYQ VVFIE YLKET LGDSL DEFTV KSFNH VFEVI

460 470 480 490 500

ISFLN EGLRQ ADIVD PVTHL TGRQK EMIKA SWSKA RTDLR SLGQE LFMRM

510 520 530 540 550

FKAHP EYQTL FVNKG FADVP LVSLR EDERF ISHMA NVLGG FDTLL QNLDE

* ***** *****

560 570 580 590 600

SSYFI YSLRN LGDAH IQRKA GTQHF RSFEA ILIPY LQESQ GLDAA SVEAW

* ***** ***

610 620 630 640 650

KKFFD VSIGV IAQGL KVATS EEADP VTGLY GKEIV ALRQA FAAVT PRNVE

*** ***** ***** * ** ***** *****

660 670 680 690 700

IGKRV FAKLF AAHPE YKNLF KKFEQ YSVEE LPSTD AFHYH ISLVM NRFSS

*** ***

710 720 730 740 750

IGKVI DDNVS FVYLL KKLGR EHIKR GLSRK QFDQF VELYI AEISS ELSDT

*** *** *****

760 770 780 790 800

GRNGL EKVLT FATGV IEQGL FQLGQ VDSNT LTALE KQSIQ DIWSN LRSTG

***

810 820 830 840 850

LQDLA VKIFT RLFSA HPEYK LLFTG RFGNV DNINE NAPFK AHLHR VLSAF

***** **

860 870 880 890 900

DIVIS TLDDS EHLIR QLKDL GLFHT RLGMT RSHFD NFATA FLSVA QDIAP

***** ***** *

910 920 930 940 950

NQLTV LGRES LNKGF KLMHG VIEEG LLQLE RINPI TGLSA REVAV VKQTW

960 970 980 990 1000

NLVKP DLMGV GMRIF KSLFE AFPAY QAVFP KFSDV PLDKL EDTPA VGKHA

1010 1020 1030 1040 1050

ISVTT KLDEL IQTLD EPANL ALLAR QLGED HIVLR VNKPM FKSFG KVLVR

*** *****

1060 1070 1080 1090 1100

LLEND LGQRF SSFAS RSWHK AYDVI VEYIE EGLQQ SYKQD PVTGI TDAEK

1110 1120 1130 1140 1150

ALVQE SWDLL KPDLL GLGRK IFTKV FTKHP DYQIL FTRTG FGDTP LTKLD

1160 1170 1180 1190 1200

DNPAF GTHII KVMRA FDHVI QILGK PKTLM AYLRS VGADH IARNV ERRHF

*** ****

1210 1220 1230 1240 1250

QAFSN ALIPV MQHEL KAQLR PDAVA AWRKG LDRII GIIDQ GLIGL KEVNP

1260 1270 1280 1290 1300

QNAFS AYDIQ AVQRT WALAK PDLMG KGAMV FKQLF TDHGY QPLFS NLAQY

1310 1320 1330 1340 1350

EITGL EGSPE LNTHA RNVMA QLDTL VGSLQ NSIEL GQSLA QLGKD HVPRK

* *****

1360 1370 1380 1390 1400

VNRVH FKDFA EHFIP LMKAD LGDEF TPLAE SAWKK AFDVM IATIE QGQRA

***** **

1410

RRSVA TFLTN PVA

...............................................................................

chain 2 (chain 1 of protein 2)

number of covered residues: 139

total number of residues: 1413

coverage [%]: 10

10 20 30 40 50

AEVRG ILCSD KATIK RTWSI VNDLP SFGRN VFLSV FAAKP EYKNL FVEFR

* ***** *

60 70 80 90 100

NIPAS ELANS ERLLY HGGRV LASID EVISE IDSPD SAAKK LVALG ERHIT

***** ***** **

110 120 130 140 150

RGTVR RHFEA FSYAF IDELK QRGVA SADLA AWRKG WDSIV DILEA GLLKR

160 170 180 190 200

QIDLE VTGLS CVDVA NVQES WATVS ANLKN TGSIL FQRLI NDHPE YQQLF

210 220 230 240 250

RQFRD VELAK LGESN GFVAH VFRVV AAFDG IIKEL DNNPF IVSTL KRLGE

***

260 270 280 290 300

QHIAR GTDIS HFQNF RTTLL VYLNE NGMNQ AQEAS WNKAF DAIEK YISIG

*****

310 320 330 340 350

LKSLG RVDPI TGLSG LEKNA ILNTW GKVRG NLQEV GKATF GKLFA AHPEY

* ***** **

360 370 380 390 400

QQMFR FFQGV QLAEL VDSPK FAAHT QRVVS ALDQT LLALN RPSDF VYMIK

410 420 430 440 450

ELGLD HINRG TDRSH FENYQ VVFVE YLKET LGDSV DEFTV KSFNH VFEVI

460 470 480 490 500

INFLN EGLRQ ANVVD PVTHL TGRQK EAIKA SWSVA RTDLR FLGQE LFMRM

510 520 530 540 550

FNLNP EYQSL FVNKG FADVP LVSLR EDERF ISHMA NVLRG FDTLL QNLDD

* ***** *****

560 570 580 590 600

TSYFV YALRN LGDAH IQRKA GTEHF RSFEA ILIPY LQESQ GLDAA GVEAW

* ***** ***

610 620 630 640 650

KIFFD VSIGV IAQGL KVASS EEADP VTGLY GKEVV ALRQA FAAIS PRNVE

** ***** *****

660 670 680 690 700

IGKRV FAKLF TSHPE YKNLF KKFEQ YSVEE LPSTD AFDYH ISLVM NRFSA

***

710 720 730 740 750

VGKVI DDNVS FVYLL KKLGR EHIKR GLSRK QFDQF VELYI AEISP ELSET

*** *****

760 770 780 790 800

GRSGL EKVLT FATGV IEQGL FQLGQ VDSKA LTALE KQSIQ DIWTS LRPTG

810 820 830 840 850

LEELA VKMFT RLFAD HPEYK LLFTG RLGNV DNINE NAPFR AHLHR VLSAF

860 870 880 890 900

DIVIT SLDNN ALLIR QLKDL GLFHT RLGMT RAHFD NFATA FFSVA EDIVP

***** ***** *

910 920 930 940 950

NLLTA LGRES LGKGF KLMVA VIEEG LLQLE RIDPI TGLSV REVEV VKQTW

** ***** * **** ***** ***** **

960 970 980 990 1000

NLVKP DLMGV GMRIF KSLFE KFPAY QAVFP KFSDV PLDKL EDIPA VGKHA

1010 1020 1030 1040 1050

ISVTT KLDEL IQTLD EPANL ALLAR QLGED HIVLG VNKPM FKSFG EVLVR

1060 1070 1080 1090 1100

LLEND LGQRF SNFAS KSWHR AYDVI VEYIE EGLQQ SYKQD PVTGI TDAEK

* ***** *****

1110 1120 1130 1140 1150

VLVQR SWELL KPDLL GLGRK IFGVI FTKHP EYQIL FTRVG FGDTP LTQLD

1160 1170 1180 1190 1200

NNPAF GEHII KVMRA FDYVI RNLGK PKTLL AYLKN VGADH IARNV ERRHF

* ***** ***

1210 1220 1230 1240 1250

QAFSE ALIPV MQREL KAQLK PEAVA AWRKG LDRII GVIDQ GLLGL KEVNP

1260 1270 1280 1290 1300

QIAFS AADIE AIQKT WALAK PDLMG KGASV FRQLF TDHGY QPLFS NLVEY

1310 1320 1330 1340 1350

EVTGL EGSPE LNTHA RNVMA QLDTL VGSLQ NSIEL GKSLN QLGKD HVPRK

* *****

1360 1370 1380 1390 1400

VNKVH FDDFA EHFVP LMKAN LGDEF TPLAE SAWKK AFNVM VATIE QGQRA

1410

RRSIA TFLTN PVA

[peak list (sorted)](http://www.searchxlinks.de/cgi-bin/upload_action.pl#contents%23contents)

******************

!! INFO upon analyzing a normal mass spectrum the program does not take peak

!! INFO intensities into account. hence, peak intensities are not printed

!! INFO here.

isd isd matching

number of parent fragment selected

assigned peak peak psd parent

mass peptides (ipp) (ifp) mass(es)

-------------------------------------------------------------------------------

802.350

803.360 [1](http://www.searchxlinks.de/cgi-bin/upload_action.pl#p3%23p3)

824.375 [2](http://www.searchxlinks.de/cgi-bin/upload_action.pl#p32%23p32)

826.340

844.360 [2](http://www.searchxlinks.de/cgi-bin/upload_action.pl#p6%23p6)

846.390

858.330

861.320

867.285 [2](http://www.searchxlinks.de/cgi-bin/upload_action.pl#p28%23p28)

889.385

899.345 [1](http://www.searchxlinks.de/cgi-bin/upload_action.pl#p17%23p17)

916.370 [2](http://www.searchxlinks.de/cgi-bin/upload_action.pl#p24%23p24)

923.450 [2](http://www.searchxlinks.de/cgi-bin/upload_action.pl#p11%23p11)

930.305

938.400

952.330 [1](http://www.searchxlinks.de/cgi-bin/upload_action.pl#p34%23p34)

960.370 [2](http://www.searchxlinks.de/cgi-bin/upload_action.pl#p26%23p26)

982.450

1012.365

1023.505 [2](http://www.searchxlinks.de/cgi-bin/upload_action.pl#p19%23p19)

1031.380 [1](http://www.searchxlinks.de/cgi-bin/upload_action.pl#p22%23p22)

1053.450

1070.315 [2](http://www.searchxlinks.de/cgi-bin/upload_action.pl#p0%23p0)

1117.440

1139.470

1147.455

1173.315 [4](http://www.searchxlinks.de/cgi-bin/upload_action.pl#p35%23p35)

1195.400 [1](http://www.searchxlinks.de/cgi-bin/upload_action.pl#p36%23p36)

1209.445

1243.530

1300.645 [4](http://www.searchxlinks.de/cgi-bin/upload_action.pl#p30%23p30)

1340.520

1381.305 [1](http://www.searchxlinks.de/cgi-bin/upload_action.pl#p9%23p9) [1381.305](http://www.searchxlinks.de/cgi-bin/upload_action.pl#pa1%23pa1)

1413.545 [1](http://www.searchxlinks.de/cgi-bin/upload_action.pl#p10%23p10)

1493.535 [3](http://www.searchxlinks.de/cgi-bin/upload_action.pl#p12%23p12)

1515.545

1531.465 [1](http://www.searchxlinks.de/cgi-bin/upload_action.pl#p14%23p14)
